# Short fiber bundle filtering and test-retest reproducibility of the Superficial White Matter

**DOI:** 10.3389/fnins.2024.1394681

**Published:** 2024-04-26

**Authors:** Cristóbal Mendoza, Claudio Román, Jean-François Mangin, Cecilia Hernández, Pamela Guevara

**Affiliations:** ^1^Department of Electrical Engineering, Faculty of Engineering, Universidad de Concepción, Concepción, Chile; ^2^Centro de Investigación y Desarrollo en Ingeniería en Salud, Universidad de Valparaíso, Valparaíso, Chile; ^3^CEA, CNRS, Neurospin, Baobab, Université Paris-Saclay, Gif-sur-Yvette, France; ^4^Department of Computer Science, Faculty of Engineering, Universidad de Concepción, Concepción, Chile; ^5^Center for Biotechnology and Bioengineering (CeBiB), Santiago, Chile

**Keywords:** Superficial White Matter, diffusion-weighted imaging, tractography, fiber bundle segmentation, spurious fibers

## Abstract

In recent years, there has been a growing interest in studying the Superficial White Matter (SWM). The SWM consists of short association fibers connecting near giry of the cortex, with a complex organization due to their close relationship with the cortical folding patterns. Therefore, their segmentation from dMRI tractography datasets requires dedicated methodologies to identify the main fiber bundle shape and deal with spurious fibers. This paper presents an enhanced short fiber bundle segmentation based on a SWM bundle atlas and the filtering of noisy fibers. The method was tuned and evaluated over HCP test-retest probabilistic tractography datasets (44 subjects). We propose four fiber bundle filters to remove spurious fibers. Furthermore, we include the identification of the main fiber fascicle to obtain well-defined fiber bundles. First, we identified four main bundle shapes in the SWM atlas, and performed a filter tuning in a subset of 28 subjects. The filter based on the Convex Hull provided the highest similarity between corresponding test-retest fiber bundles. Subsequently, we applied the best filter in the 16 remaining subjects for all atlas bundles, showing that filtered fiber bundles significantly improve test-retest reproducibility indices when removing between ten and twenty percent of the fibers. Additionally, we applied the bundle segmentation with and without filtering to the ABIDE-II database. The fiber bundle filtering allowed us to obtain a higher number of bundles with significant differences in fractional anisotropy, mean diffusivity, and radial diffusivity of Autism Spectrum Disorder patients relative to controls.

## 1 Introduction

Diffusion magnetic resonance imaging (dMRI) tractography (Basser et al., 2000) is the only available tool able to reconstruct the brain's White Matter (WM) pathways non-invasively. Tractography algorithms infer the WM anatomy as a set of 3D streamlines, which can be used for *in vivo* virtual dissection of anatomically meaningful tracts, also known as fiber bundle segmentation. The delineation of individual fiber bundles has enabled a quantitative comparison of the WM pathways across different subjects (Zhang et al., 2022).

Most methods for fiber bundle segmentation are designed for the commissural, projection, and long-range association connections within the Deep White Matter (DWM). These tracts are arranged in large and stable bundles with a well-known anatomy and description of their trajectory. Therefore, their automatic extraction has achieved remarkable results over the years (O'Donnell and Westin, 2007; Guevara et al., 2012; Wassermann et al., 2016; Garyfallidis et al., 2018; Wasserthal et al., 2018; Bertò et al., 2021). However, these methods have not been adapted to the Superficial White Matter (SWM); specifically, there is no deep study and performance evaluation on short association fiber bundles.

The SWM refers to the short association fibers just below the brain cortex, connecting close regions of the same brain hemisphere (Guevara et al., 2020). They can run along a gyrus, surround it, or skip one or more convolutions. Their clinical relevance has been assessed in multiple diseases such as Schizophrenia (Nazeri et al., 2013; Kai et al., 2023), Autism Spectrum Disorder (ASD) (d'Albis et al., 2018; Hong et al., 2018), Multiple Sclerosis (Buyukturkoglu et al., 2021), Alzheimer's disease (Reginold et al., 2016) and Parkinson's disease (Zhang et al., 2021). Recently, a study of changes in cognitively normal aging revealed a correlation between age and microstructural features of the SWM (Schilling et al., 2023).

Recent progress in dMRI acquisitions, using techniques such as High Angular Resolution Diffusion Imaging (HARDI), coupled with improved estimations of fiber orientations, has significantly enhanced the capabilities of tractography algorithms in reconstructing fiber bundles within the SWM. Hence, their research is gaining increasing importance in understanding of the WM structure and function (Guevara et al., 2020). In this context, new fiber bundle segmentation methods consider short bundles partially (Bertò et al., 2021) and in a dedicated manner (Vindas et al., 2023; Xue et al., 2023). Also, works based on fiber clustering have constructed several SWM fiber bundle atlases containing a high number of short bundles throughout the whole brain (Guevara et al., 2017; Zhang et al., 2018; Román et al., 2022), enabling the identification of short bundles in new subjects by, for example, labeling the fibers to the closest atlas bundle. Furthermore, methods employing Regions of Interest (ROIs) can extract short bundles connecting specific regions within a brain anatomical parcellation. However, such approaches may also segment a high proportion of spurious fibers (Guevara et al., 2020).

Despite technological and methodological advances, short fiber bundles are difficult to segment because of their smaller size and complex shape due to their proximity to the gyral crowns and sulcal walls. Other difficulties arise from the high inter-subject variability, artifacts produced near the cortex, and the ill-posed nature of the tractography, which implies that the tracking results could be affected by ambiguous voxels or minor changes in acquisition noise (Mangin et al., 2013). Consequently, most segmentation algorithms also label spurious fibers. Furthermore, segmentation of well-defined short bundles becomes more difficult considering that probabilistic tracking is needed to properly reconstruct SWM bundles, which also generates a high proportion of noisy fibers (Guevara et al., 2020).

Some methods remove noisy fibers by using tract probability maps (Yeatman et al., 2012; Sommer et al., 2016) or density maps (Aydogan and Shi, 2015; Yeh et al., 2018) to generate compact bundles. Others use the distance between the fiber points (Jordan et al., 2017; Wang et al., 2018; Xia and Shi, 2020), and fiber clustering to remove outliers (Cousineau et al., 2017; Wasserthal et al., 2018; Schilling et al., 2023). However, most of these methods are not specific for SWM bundles.

This work proposes several filters to improve a well-established segmentation method based on a multi-subject atlas, developed in Guevara et al. (2012), which provides a straightforward way to label subject's fibers to the closest atlas bundle. Thus, we can study many short bundles across the whole brain using new SWM bundle atlases with high cortical coverage (e.g., Román et al., 2022). Because this segmentation method was proposed for the main DWM bundles, it does not work well on SWM bundles. A visual inspection of the segmented SWM bundles suggests two main problems: spurious fibers and the segmentation of fibers whose shape differs from the main atlas bundle shape. Next, we describe our two main contributions.

**Figure 1:**
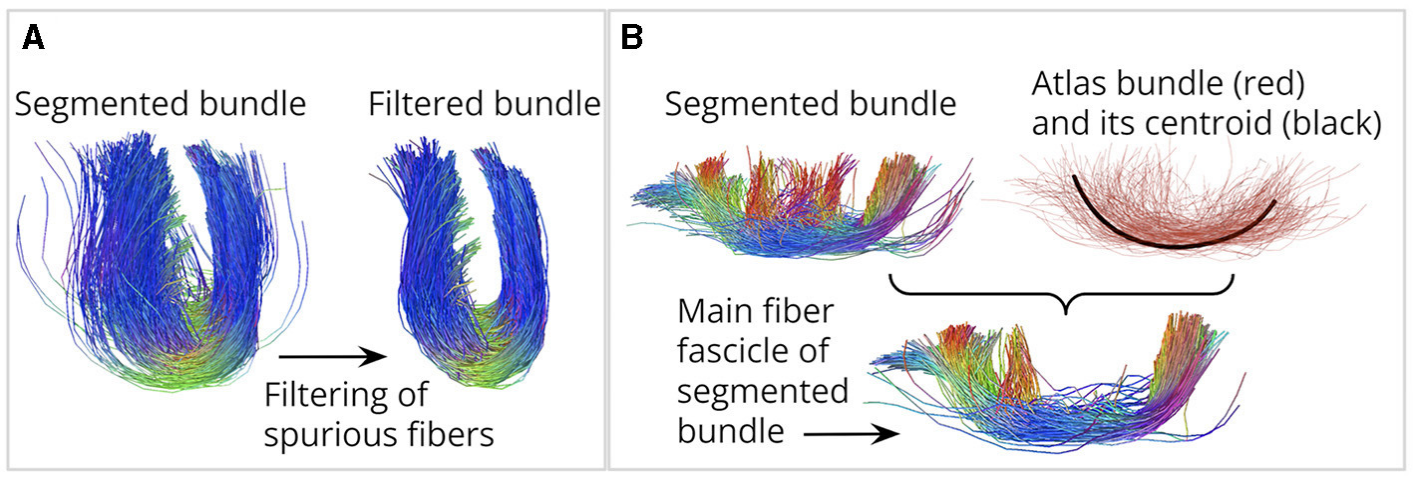
Contributions of this work. **(A)** We designed four fiber bundle filters to remove spurious fibers. **(B)** We propose a method to identify the main fiber fascicle. On the right, an atlas bundle (red) and its centroid (black). The segmented bundle is shown on the left. It can be seen that fibers toward the middle of the segmented bundle deviate from the main atlas shape. Below is the main fiber fascicle, containing fibers following a similar trajectory to the atlas bundle centroid.

i) We propose four different fiber bundle filters (see Figure 1A) to deal with the segmentation of spurious fibers. The filters consider the spatial characteristics of noisy fibers. The filter based on Connectivity Patterns quantifies the endpoint similarity between fibers. The filter based on the Symmetric Segment-Path Distance (SSPD) (Besse et al., 2016) computes the similarity between the fiber trajectories. The filter based on Fiber Consistency assigns a consistency measure to each fiber point (Xia and Shi, 2020), computed from the fiber's proximity to its K-nearest fibers. Finally, the filter based on the Convex Hull represents a fiber bundle as a point cloud. Then, the Convex Hull of the point cloud generates an envelope that enables the detection of fibers far away from the core.

ii) We introduce the *identification of the main fiber fascicle*, aiming to discard the segmented fibers whose shape differs from the main atlas bundle shape (see Figure 1B). These fibers cannot be removed with the filters described above because they are not isolated and can be located at the core of the bundle. The identification of the main fiber fascicle solves this problem by removing segmented fibers that do not follow a trajectory similar to the centroid of the atlas bundle, that is, a single fiber that properly describes the shape of the main atlas bundle.

Test-retest reproducibility indices were calculated to evaluate the effectiveness of fiber bundle filters in removing spurious fibers. The analysis of repeated scans has been previously used to assess the reliability of segmentation methods (Zhang et al., 2019; Schilling et al., 2021a,b). More specifically, it provides insight into whether the same WM structure can be accurately reproduced from two repeated acquisitions of a subject. Nonetheless, results could be less consistent due to false positive connections or spurious fibers (Zhang et al., 2019). Additionally, the identification of the main fiber fascicle and the best fiber bundle filter were used to detect alterations in diffusion-based microstructural indices within subjects with Autism Spectrum Disorder (ASD) from the ABIDE-II database (Martino et al., 2017) relative to controls.

## 2 Materials and methods

### 2.1 Diffusion MRI and tractography datasets

#### 2.1.1 HCP database

We used 44 subjects (13M, 31F; aged 22–35 years old) with retest acquisition from the HCP database (Human Connectome Project, 2017). The database contains two sequences of multi-shell HARDI data for each subject (test-retest interval: 4.6 ± 2 months). The dMRI data was collected for three shells at b-values of 1,000, 2,000, and 3,000 *s*/*mm*^2^ and a total of 270 directions, with an isotropic voxel of 1.25 *mm*. We used HCP preprocessed data (Glasser et al., 2013) with diffusion image distortion correction (Andersson et al., 2003; Andersson and Sotiropoulos, 2015). Furthermore, we used the MRtrix software (Tournier et al., 2019) to compute the whole-brain probabilistic tractography based on Constrained Spherical Deconvolution (CSD) (Tournier et al., 2007) and second order integration over the fiber orientation distribution (iFOD2), with default parameters. For each subject, tractography datasets were computed for the two test-retest dMRI scans with the following parameters: step size of 0.625 *mm*, angle threshold of 90°, maximum length of 250 *mm*, minimum length of 30 *mm* and FOD amplitude threshold of 0.06. We used Anatomically-Constrained Tractography (ACT) (Smith et al., 2012) to obtain 30 million streamlines and applied Spherical-deconvolution Informed Filtering of Tractograms (SIFT) (Smith et al., 2013) to maintain 10% of the fibers. Final tractograms with 3 million streamlines were transformed to MNI space using the non-linear transformation provided by the HCP data and resampled with 21 equidistant points (Guevara et al., 2012).

#### 2.1.2 ABIDE-II database

We used 44 subjects with T1-weighted and single shell HARDI acquisitions from the ABIDE-II database (Martino et al., 2017). Subjects were selected from the NYU Langone Medical Center, comprising 22 controls (21 male, one female; 9.8 ± 3.6 years old) and 22 Autism Spectrum Disorder (ASD) (21 male, one female; 9.8 ± 5.6 years old). The single shell HARDI data were acquired on a scanner Siemens MAGNETOM Allegra syngo MR 2004A (64 directions, b-value of 1,000 *s*/*mm*^2^) with an isotropic voxel of 3 *mm*. Also, T1-weighted images with a voxel size of 1.3 × 1.0 × 1.3 *mm* were available. Each dMRI dataset was preprocessed using MRtrix software (Tournier et al., 2019), including denoising, motion, and distortion correction. Whole-brain probabilistic tractography based on CSD (Tournier et al., 2007) (with default parameters) and iFOD2 tracking were calculated using MRtrix. For each subject, tractography datasets were computed with the following parameters: step size of 1.5 *mm*, angle threshold of 90°, maximum length of 250 *mm*, minimum length of 30 *mm* and FOD amplitude threshold of 0.06. We used ACT (Smith et al., 2012) to obtain 30 million streamlines, applied SIFT (Smith et al., 2013) to maintain 10% of the fibers, and the final 3 million streamlines were transformed into MNI space. For this purpose, the T1 and dMRI images were coregistered using FSL software (Jenkinson et al., 2012). Then, the T1 images were normalized to MNI space using Advance Normalization Tools (ANTs) (Avants et al., 2011). Finally, tractograms were transformed into MNI space and resampled with 21 equidistant points for further analysis.

### 2.2 Automatic segmentation method and SWM bundle atlas used

The automatic segmentation method proposed in Guevara et al. (2012) labels each subject's fiber to the closest atlas bundle based on the *D*_*ME*_ distance and a length penalization term (*NT*). First, the *D*_*ME*_ between a subject's fiber *S* and an atlas bundle fiber *A* is computed in Equation (1):


(1) $$\begin{array}{l} D_{ME}\left(S,A\right)=\min\left(\underset{i}{\max}||s_{i}-a_{i}||,\underset{i}{\max}||s_{i}-a_{N-i}||\right) \end{array}$$

where *s*_*i*_ and *a*_*i*_ are corresponding 3D points of fibers *S* and *A*, respectively. Also, *N* is the number of points of the fibers. Then, the *NT* term is calculated in Equation (2):


(2) $$\begin{array}{l} NT={\left(\frac{abs\left(l_{S}-l_{A}\right)}{max\left(l_{S},l_{A}\right)}+1\right)}^{2}-1 \end{array}$$

where *l*_*S*_ and *l*_*A*_ are the lengths of fibers *S* and *A*, respectively. Finally, the *D*_*NE*_ distance is computed in Equation (3):


(3) $$\begin{array}{l} D_{NE}\left(S,A\right)=d_{ME}\left(S,A\right)+NT \end{array}$$

Each subject's fiber *S* is labeled with the atlas bundle *j* if the distance *D*_*NE*_ to fiber *A* in bundle *j* is less than a threshold in millimeters. We used the latest version of the segmentation algorithm (Vázquez et al., 2019), which exploits thread-level parallelism on multiple CPUs. We leveraged a new SWM multi-subject bundle atlas (Román et al., 2022) constructed from 100 healthy subjects of the HCP database and probabilistic tractography. The atlas is composed of 525 short fiber bundles across the whole brain. Segmentation thresholds use the mean length of the fibers composing the atlas bundle and a linear mapping (between 6 and 8 *mm*).

### 2.3 SWM atlas processing

The main goal was to identify the main bundle shapes present in the SWM bundle atlas. For each atlas bundle, we calculated a single fiber representing the overall shape of the bundle (centroid), considering the fiber length, shape, and position. Then, we applied an alignment to overlap the fiber geometry of every pair of atlas bundle centroids. The alignment allowed us to disregard their spatial position and to focus only on shape differences based on the distance between centroid points. We also used a scaling factor to remove the differences caused by the centroid length. We computed the distance matrix between every pair of aligned centroids. Then, we applied an average-link hierarchical clustering, identifying that four clusters were optimal using the Within Clusters Sum of Squares (WCSS) criterion (Aggarwal and Reddy, 2018).

**Figure 2:**
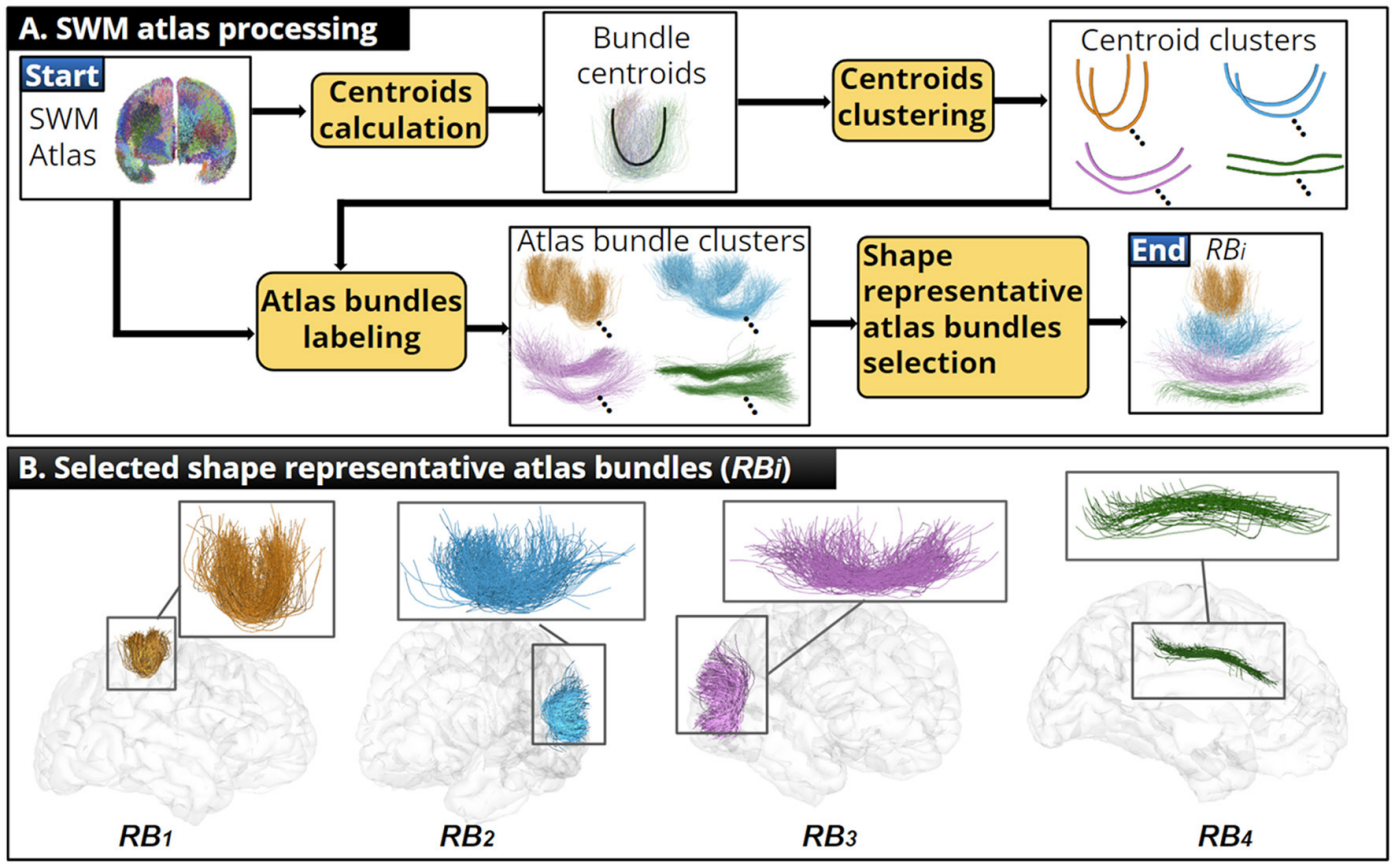
Illustration of the SWM bundle atlas processing. **(A)** Summary of the SWM atlas processing, where an adequate centroid was calculated for each atlas bundle and shape representative atlas bundles were identified. **(B)** We show each *RB*_*i*_ and their position within the human brain.

We identified and grouped the corresponding atlas bundles using the resulting clusters. Finally, for each atlas cluster, we selected a shape representative bundle (*RB*_*i*_). This step helped us to generalize the atlas information into a few bundles and mitigate the risk of overfitting the fiber bundle filters' parameters. We summarize the SWM atlas processing and show the selected *RB*_*i*_ in Figure 2. See Supplementary material S1 for more details about each step performed. Additional results on the quality of the atlas bundle centroids are shown in Supplementary material S2.

### 2.4 Identification of the main fiber fascicle

**Figure 3:**
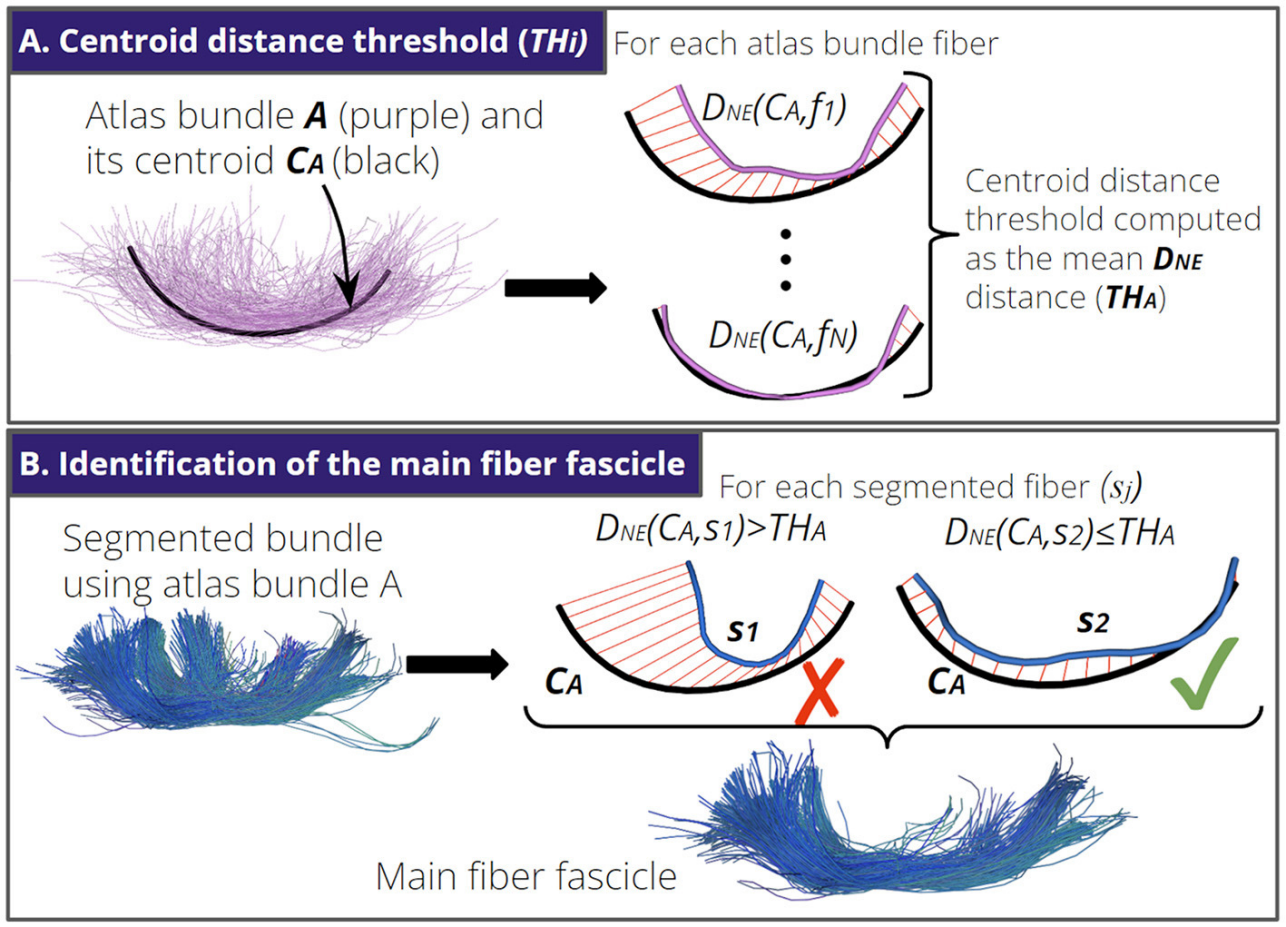
Identification of the main fiber fascicle. **(A)** An atlas bundle *A* with *N* fibers and its centroid *C*_*A*_. First, we compute the distance *D*_*NE*_ between each fiber *f*_*i*_ of *A* and *C*_*A*_. Next, we calculate the mean *D*_*NE*_ distance as the centroid distance threshold for atlas bundle *A* (*TH*_*A*_). **(B)** Illustration of the identification of the main fiber fascicle. We show a segmented fiber bundle using atlas bundle *A*. Next, we remove segmented fibers *s*_*j*_ with a *D*_*NE*_(*s*_*j*_, *C*_*A*_)>*TH*_*A*_. Here, fiber *s*_1_ is removed because its shape differs from the shape of centroid *C*_*A*_.

The identification of the main fiber fascicle aims to remove segmented fibers that do not follow a trajectory similar to the shape of the main atlas bundle. First, for each atlas bundle *i*, we computed a centroid distance threshold (*TH*_*i*_). The *TH*_*i*_ is the mean *D*_*NE*_ distance of the atlas bundle fibers to the corresponding atlas bundle centroid (see Figure 3A). Then, given a segmented fiber bundle *S* using the atlas bundle *i*, the identification of the main fiber fascicle consists of computing the *D*_*NE*_ distance between each segmented fiber *s*_*j*_∈*S* and the centroid of the atlas bundle *i*. Segmented fibers with a *D*_*NE*_ distance higher than the *TH*_*i*_ are discarded (see Figure 3B). In practice, the average value of the *TH*_*i*_ for all atlas bundles was 15 ± 2 *mm*.

### 2.5 Fiber bundle filters

**Figure 4:**
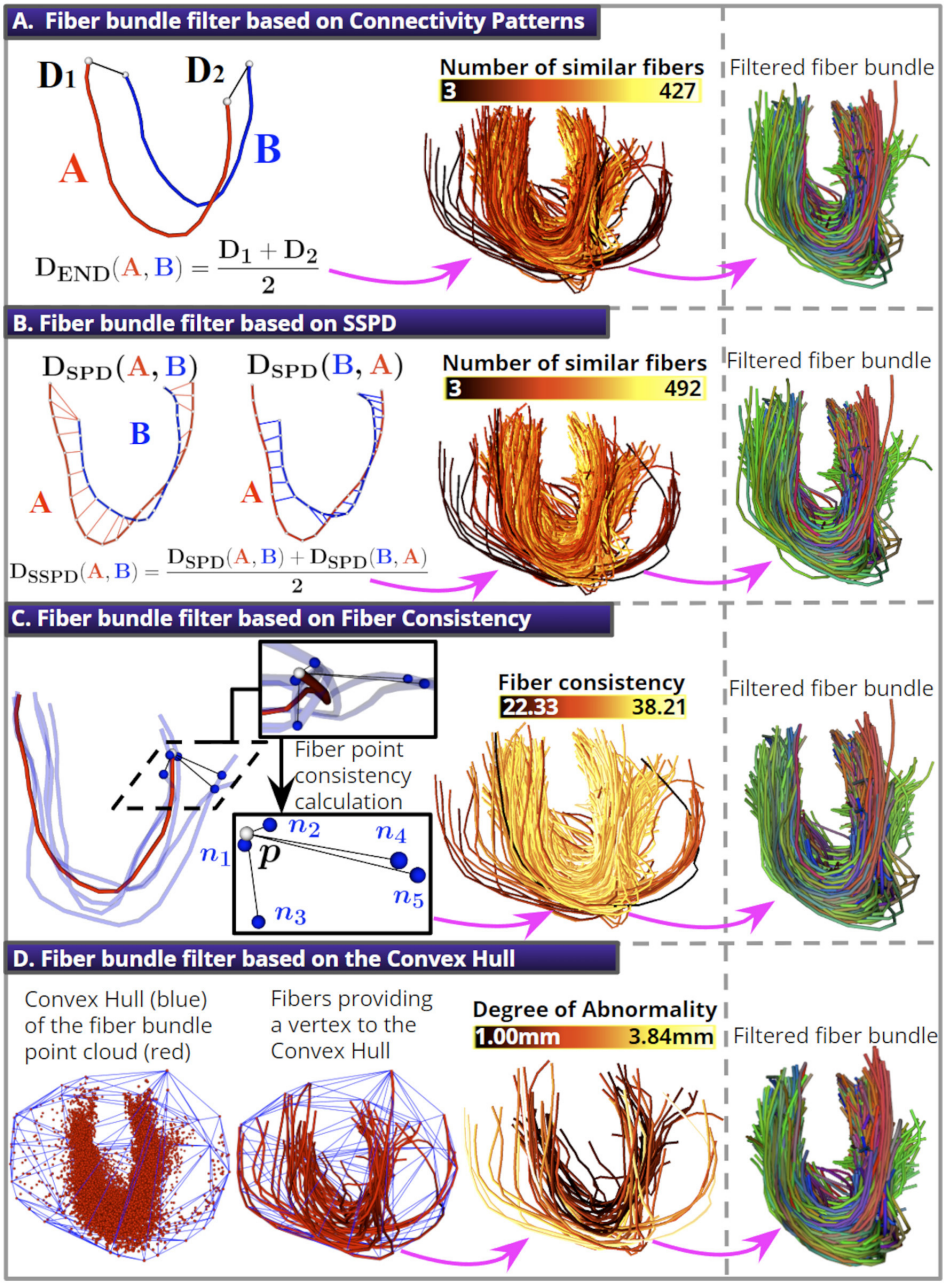
The four fiber bundle filters. **(A)** The *D*_*END*_ distance removes fibers with noisy endpoint positions. **(B)** The *D*_*SSPD*_ distance is used to remove fibers with noisy trajectories. **(C)** A schematic of the fiber point consistency calculation for the red fiber's point **p** (white sphere) using *K*_*f*_ = 5 (blue fibers). **(D)** An illustration of the segmented bundle's point cloud and its Convex Hull (10% of the fibers were discarded for all filters).

This section presents four fiber bundle filters based on different approaches to remove isolated or spurious fibers from segmented bundles. In Figure 4, we show a schematic of each fiber bundle filter and how their application can be used to obtain a short bundle with fewer outliers.

#### 2.5.1 Fiber bundle filter based on Connectivity Patterns

The fiber bundle filter based on Connectivity Patterns (CP) uses the Euclidean distance between corresponding endpoints of two fibers as a criterion to remove spurious fibers. The idea is that fibers with nearby endpoints have a similar pattern of anatomical connectivity. We used the *D*_*END*_ distance (Bertò et al., 2021) to quantify the endpoint similarity between two fibers A and B with *N* points, which is defined in Equation (4):


(4) $$\begin{array}{l} D_{END}\left(A,B\right)=\frac{1}{2}(\min(\left\|a_{1}-b_{1}\right\|,\left\|a_{1}-b_{N}\right\|)\\ \;+\min(\left\|a_{N}-b_{1}\right\|,\left\|a_{N}-b_{N}\right\|)) \end{array}$$

where {*a*_1_, *a*_*N*_} and {*b*_1_, *b*_*N*_} are the endpoints of fiber *A* and *B*, respectively. Next, we describe how the filter works. First, we calculate each fiber's *D*_*END*_ distance to every other fiber in the bundle, and count the number of similar fibers as those with a *D*_*END*_ less than a threshold called θ_*END*_. Finally, we discard a percentage of fibers with the lowest number of similar fibers (see Figure 4A). This fiber bundle filter has two parameters: the Percentage of Discarded Fibers (PDF) and the θ_*END*_ distance threshold.

#### 2.5.2 Fiber bundle filter based on Symmetric Segment-Path Distance

This fiber bundle filter compares fiber trajectories using the Symmetric Segment-Path Distance (SSPD) (Besse et al., 2016). The SSPD considers length differences between trajectories and the possibility that they might be spatially close yet have a different shape. The computation of the SSPD between two fibers A and B is described next. First, the point-to-segment distance (*D*_*ps*_) is defined in Equation (5):


(5) $$D_{ps}\left(a_{i},s_{j}^{B}\right)=\{\begin{array}{c} \left\|a_{i}-a_{i}^{proj}\right\|,\;if\;a_{i}^{proj}\in s_{j}^{B}\\ \min(\left\|a_{i}-b_{j}\right\|,\left\|a_{i}-b_{j+1}\right\|)\;,\;otherwise \end{array}$$

where, *a*_*i*_ is a 3D point of fiber A and $a_{i}^{proj}$ is the orthogonal projection of *a*_*i*_ on the segment $s_{j}^{B}$ of fiber B. Also, *b*_*j*_ and *b*_*j*+1_ are two 3D points composing the line segment $s_{j}^{B}$ of fiber B. Next, the point-to-trajectory distance (*D*_*pt*_) is computed in Equation (6):


(6) $$\begin{array}{l} D_{pt}\left(a_{i},B\right)=\underset{j\in \left[1,...,N-1\right]}{\min}D_{ps}\left(a_{i},s_{j}^{B}\right) \end{array}$$

where *N* is the number of points of the fibers. Then, the Segment-Path-Distance (SPD) from fiber A to fiber B is calculated in Equation (7):


(7) $$\begin{array}{l} D_{SPD}\left(A,B\right)=\frac{1}{N}\overset{N}{\underset{i=1}{\sum }}D_{pt}\left(a_{i},B\right) \end{array}$$

Finally, the SSPD distance is calculated in Equation (8):


(8) $$\begin{array}{l} D_{SSPD}\left(A,B\right)=\frac{D_{SPD}\left(A,B\right)+D_{SPD}\left(B,A\right)}{2} \end{array}$$

The filter works in the same manner as described for the filter based on Connectivity Patterns. First, we calculate the distance *D*_*SSPD*_ from each fiber to all other fibers in the bundle. Then, we count the number of similar fibers as those with a *D*_*SSPD*_ distance less than a distance threshold called θ_*SSPD*_. Finally, we discard a percentage of fibers with the lowest number of similar fibers (see Figure 4B). This fiber bundle filter has two parameters: the PDF and the θ_*SSPD*_ distance threshold.

#### 2.5.3 Fiber bundle filter based on Fiber Consistency

This fiber bundle filter uses the concept of fiber point consistency (Xia and Shi, 2020) to remove spurious fibers. It uses the Minimum average Direct-Flip (MDF) distance (Garyfallidis et al., 2012) to quantify the spatial proximity between two fibers. The MDF distance between fibers A and B with *N* points computes the average distance between corresponding fiber points (Equation 9):


(9) $$D_{MDF}\left(A,B\right)=\min(\frac{1}{N}\sum_{i=1}^{N}\left\|a_{i}-b_{i}\right\|,\frac{1}{N}\sum_{i=1}^{N}\left\|a_{i}-b_{N-i+1}\right\|)$$

where *a*_*i*_ and *b*_*i*_ are corresponding fiber points. For each fiber *f*_*i*_ in the bundle, we denote its set of *K*-nearest fibers by $F_{i}=\left\{f_{j}|j=1,...,K\right\}$. These fibers are spatially closer to *f*_*i*_, calculated using the MDF distance. Then, given any fiber point *p*∈*f*_*i*_, we define its neighborhood point set as $P=\left\{n_{j}|j=1,...,K\right\}$ where *n*_*j*_ is the closest point to *p* on fiber $f_{j}\in F_{i}$. Then, the consistency measure at the fiber point *p* is calculated in Equation (10) (Xia and Shi, 2020):


(10) $$C(p)=\sum_{j=1}^{K}e^{-\frac{||p-n_{j}|{|}^{2}}{\sigma_{c}^{2}}},n_{j}\;\in \;P$$

The parameter σ_*c*_ controls the quantitative conversion from point-wise distance to point-wise affinity. The authors in Xia and Shi (2020) found that a σ_*c*_ = 6 − 8 *mm* is an appropriate value; therefore, we used a σ_*c*_ = 8 *mm*. Then, we calculate the fiber consistency as the average consistency for the streamline points. Isolated fibers tend to have low consistency because the factor $e^{-\frac{||p-n_{j}|{|}^{2}}{\sigma_{c}^{2}}}$ decays exponentially as the distance ||*p*−*n*_*j*_|| increases. Finally, we discard a percentage of fibers with the lowest consistency (see Figure 4C). This fiber bundle filter has two parameters: the PDF and the *K*-nearest fibers (*K*_*f*_).

#### 2.5.4 Fiber bundle filter based on the Convex Hull

This fiber bundle filter uses the Convex Hull (CH) (Kai et al., 2022) to remove spurious fibers. First, we represent a fiber bundle as a point cloud. Next, we calculate the CH of the point cloud using the Qhull algorithm from the Python Scipy package (Virtanen et al., 2020). The CH is the smallest convex set that contains all the points (see Figure 4D). We defined a fiber Degree of Abnormality (DA), calculated for fibers *f*_*i*_ with at least one vertex belonging to the CH. For the DA calculation, we first compute the mean Euclidean distance from each *f*_*i*_ point to its *K*-nearest points in the point cloud and calculate the average value for the streamline points. Fibers with a DA greater than one standard deviation from the mean DA are removed. The algorithm discards fibers until a defined percentage is reached. This fiber bundle filter has two parameters: the PDF and the number of *K*-nearest points (*K*_*p*_).

### 2.6 Test-retest reproducibility indices

We computed reproducibility indices to quantify the similarity between the corresponding test-retest fiber bundles. We employed measurements widely used in the field. We used the Dice Volumetric Overlap (Bertò et al., 2021), Average Fractal Dimension (AFD) (Bertò et al., 2021), Average Minimum Distance (AMD) (Schilling et al., 2021a), and Average Distance (AD) (Guevara et al., 2012).

#### 2.6.1 Indices based on the distance between fibers

Given two bundles, $B_{1}=\left[f_{1}^{1},...,f_{N_{1}}^{1}\right]$ with *N*_1_ fibers and $B_{2}=\left[f_{1}^{2},...,f_{N_{2}}^{2}\right]$ with *N*_2_ fibers, we calculate the Average Distance (AD) (Guevara et al., 2012) and Average Minimum Distance (AMD) (Schilling et al., 2021a) in Equations (11, 12), respectively:


(11) $$\begin{array}{l} AD\left(B_{1},B_{2}\right)=\frac{1}{N_{1}\times N_{2}}\overset{N_{1}}{\underset{i=1}{\sum }}\overset{N_{2}}{\underset{j=1}{\sum }}D_{ME}\left(f_{i}^{1},f_{j}^{2}\right) \end{array}$$

(12) $$\begin{array}{l} AMD\left(B_{1},B_{2}\right)=(\frac{1}{N_{1}}\sum_{i=1}^{N_{1}}\underset{j}{\min}\;D_{ME}\left(f_{i}^{1},f_{j}^{2}\right)\\ \;+\frac{1}{N_{2}}\sum_{j=1}^{N_{2}}\underset{i}{\min}\;D_{ME}\left(f_{j}^{2},f_{i}^{1}\right))/2 \end{array}$$

The AD indicates the spatial proximity between two fiber bundles. Whereas, the AMD indicates the average distance of disagreement when two bundles have fibers with different geometries. Both the AMD and AD are in millimeters.

#### 2.6.2 Indices based on bundle binary masks

Given two bundle binary masks *M*_1_ and *M*_2_, the Dice Volumetric Overlap (Bertò et al., 2021) and the Average Fractal Dimension (AFD) are calculated in Equations (13, 14), respectively:


(13) $$\begin{array}{l} DSC\left(M_{1},M_{2}\right)=\frac{2\times |v\left(M_{1}\right)\cap v\left(M_{2}\right)|}{\left|v\left(M_{1}\right)|+|v\left(M_{2}\right)\right|} \end{array}$$

where, |*v*()| is the number of voxels in the bundle mask. A Dice Score Coefficient (DSC) of 1 indicates perfect overlap, whereas a DSC of 0 indicates that bundle masks do not overlap.


(14) $$\begin{array}{l} AFD\left(M_{1},M_{2}\right)=\left(FD_{box}\left(M_{1}\right)+FD_{box}\left(M_{2}\right)\right)/2 \end{array}$$

where *FD*_*box*_ is the Fractal Dimension (FD) (Bertò et al., 2021) of the bundle mask. The FD quantify the degree of regularity/smoothness of a 3D shape and it is computed using the *box-counting dimension*, which covers a shape with boxes of size δ and it quantifies how the number of boxes varies as δ changes, in double log scale (Equation 15):


(15) $$FD_{box}(M)=-\underset{\delta \to 0}{\lim}\frac{\log\text{ count}(\delta )}{\log(\delta )}$$

where count(δ) is the number of boxes required to cover the bundle mask *M*. A high FD indicates that bundles are smooth and rounded, whereas a low FD means that bundles are wrinkled and irregular (Bertò et al., 2021). Therefore, we propose Equation (14) to quantify the average degree of smoothness/regularity between two bundle masks. In Bertò et al. (2021) the authors calculated the FD of segmented fiber bundles, obtaining values between 1.7 and 2.5.

### 2.7 Enhanced fiber bundle segmentation in the HCP database

**Figure 5:**
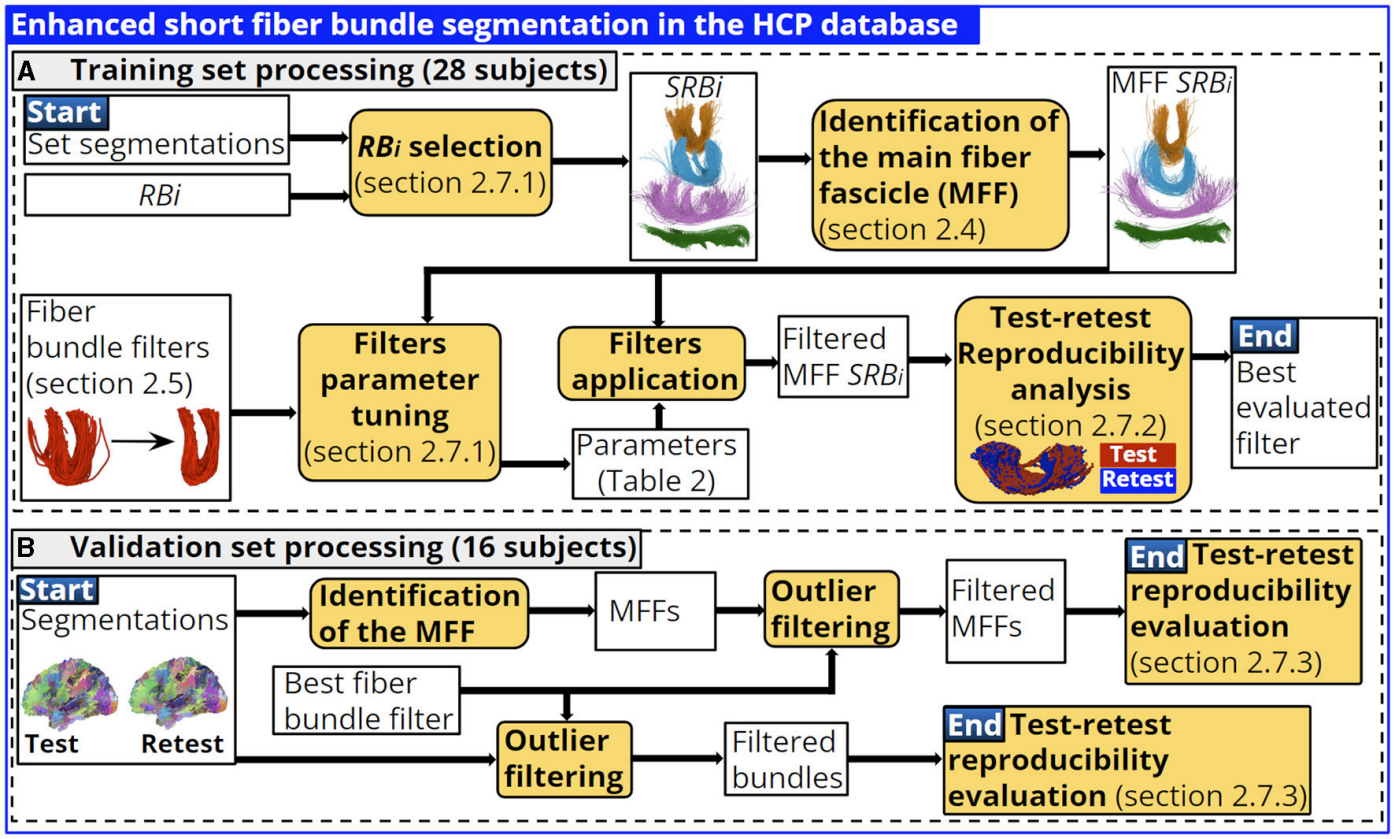
Training and validation set processing. In training we performed a parameter tuning for each fiber bundle filter and each shape representative bundle identified. We determined the best fiber bundle filter based on test-retest reproducibility indices. The best filter performance is evaluated in the validation set considering all atlas bundles. **(A)** Training set processing (28 subjects). **(B)** Validation set processing (16 subjects).

This section presents the methodology implemented to enhance the segmentation of the short fiber bundles based on the identification of the main fiber fascicle and the fiber bundle filters. The database was divided into two groups of randomly chosen subjects. In training (28 subjects), we used four segmented representative bundles, each one corresponding to a main bundle shape identified in the bundle atlas, to perform a filter parameter tuning and determine the best filter (see Figure 5A). The validation set comprises 16 subjects and was used to evaluate the best fiber bundle filter performance using all atlas bundles, with and without the identification of the main fiber fascicle (see Figure 5B).

#### 2.7.1 Filter parameter tuning in the training set

The segmented bundles from the *RB*_*i*_ (see Section 2.3) are denoted *SRB*_*i*_. Also, the *SRB*_*i*_ processed with the identification of the main fiber fascicle (see Section 2.4) are denoted as MFF *SRB*_*i*_.

In the following, we describe the main concepts used to formulate a cost function, which is used to compute appropriate parameters for each fiber bundle filter. We considered two observations from the test-retest bundles. First, spurious fibers in the retest bundle are located distant from the core of the test bundle. Thus, these distant fibers have a high *D*_*ME*_ distance. Second, these fibers have a large difference in the number of neighboring fibers surrounding their trajectory. To estimate the number of neighboring fibers, we calculated a bundle density image and averaged the value of the underlying image along the fiber's path. The bundle density image calculation is described in Supplementary material S3. Next, we introduce the *DT* term in Equation (16), designed to penalize the difference in the number of neighboring fibers.


(16) $$\begin{array}{l} DT\left(f_{i},f_{j}\right)={\left(\frac{abs\left(NS_{i}-NS_{j}\right)}{max\left(NS_{i},NS_{j}\right)}+1\right)}^{2} \end{array}$$

where *NS*_*i*_ and *NS*_*j*_ are the number of neighboring fibers of streamlines *f*_*i*_ and *f*_*j*_, respectively. The term *DT* is 1.0 when *NS*_*i*_ and *NS*_*j*_ are equal and increases as the difference becomes larger.

Subsequently, we formulate a cost function to estimate the fit between the corresponding test-retest main fiber fascicles. Our cost function is denoted as Test-Retest Maximum Distance [TRMD, is inspired by the cost function developed in Garyfallidis et al. (2015)]. Given a test MFF *SRB*_*i*_ with fibers $B_{1}=\left[f_{1}^{1},...,f_{N_{1}}^{1}\right]$ and its corresponding retest MFF *SRB*_*i*_ with fibers $B_{2}=\left[f_{1}^{2},...,f_{N_{2}}^{2}\right]$, the TRMD is calculated in Equation (17):


(17) $$\begin{array}{l} TRMD\left(B_{1},B_{2}\right)=(\frac{1}{N_{1}}\sum_{i=1}^{N_{1}}\underset{j}{\max}D_{ME}\left(f_{i}^{1},f_{j}^{2}\right)\times DT\left(f_{i}^{1},f_{j}^{2}\right)\\ \;+{\frac{1}{N_{2}}\sum_{j=1}^{N_{2}}\underset{i}{\max}D_{ME}\left(f_{j}^{2},f_{i}^{1}\right)\times DT\left(f_{j}^{2},f_{i}^{1}\right))}^{2} \end{array}$$

**Figure 6:**
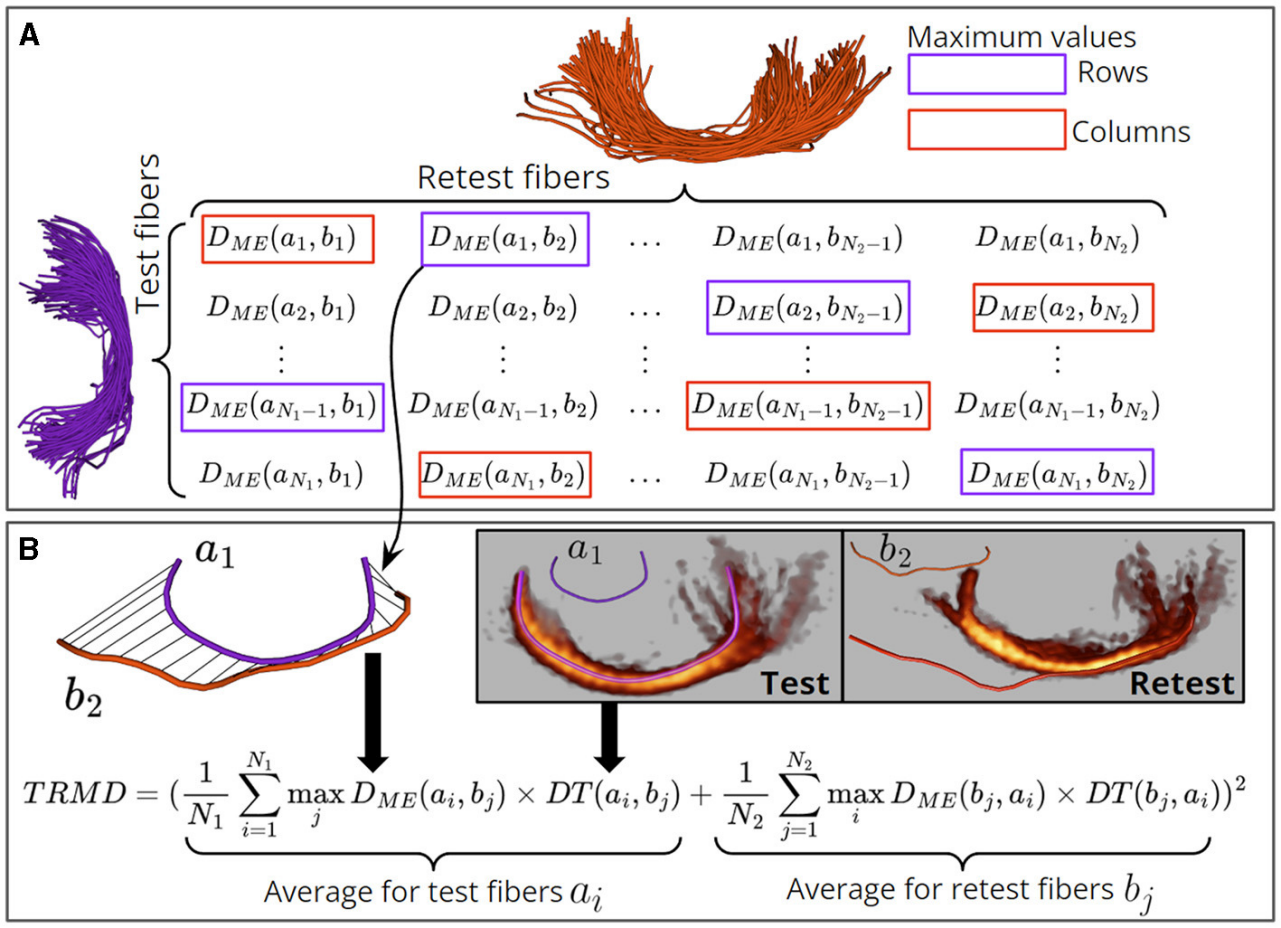
Illustration of the TRMD computation between a pair of test and retest bundles. **(A)** A rectangular matrix is computed that contains the *D*_*ME*_ distance between every pair of fibers of the test and retest bundles. Then, the maximum value of *D*_*ME*_ (max *D*_*ME*_) is calculated for each row and column. **(B)** A pair of fibers with a max *D*_*ME*_ is identified (fibers *a*_1_ and *b*_2_) and their distance is penalized by the difference of density in their neighboring fibers (*DT* term). The TRMD is calculated by summing the average of these penalized distances for the test (rows) and retest (columns) fibers, and the final value is squared. Spurious fibers (e.g., *b*_2_) are located far away from the core fibers of the bundle (e.g., *a*_1_), resulting in a high *D*_*ME*_ distance. Also, these fibers are located in regions with low fiber density, leading to a high *DT* term. Therefore, test-retest bundles with spurious fibers have a high TRMD value. Bundle density images are shown with a gray background and color coded with red (low density)—yellow (high density).

In the TRMD, we compute the maximum *D*_*ME*_ distance from each fiber in *B*_1_ to all fibers in *B*_2_ (and vice-versa for fibers in *B*_2_). Furthermore, these maximum distances are penalized by the term *DT* and averaged. The intuition is that the maximum distance *D*_*ME*_ of fibers in the core of *B*_1_ will correspond to distant and isolated fibers in *B*_2_ (and likewise for fibers in the core of *B*_2_). Consequently, the TRMD will be high due to the *DT* term. As we remove spurious streamlines using a fiber bundle filter, the TRMD will drop sharply and stabilize. See Figure 6 for an illustration of the TRMD computation.

**Figure 7:**
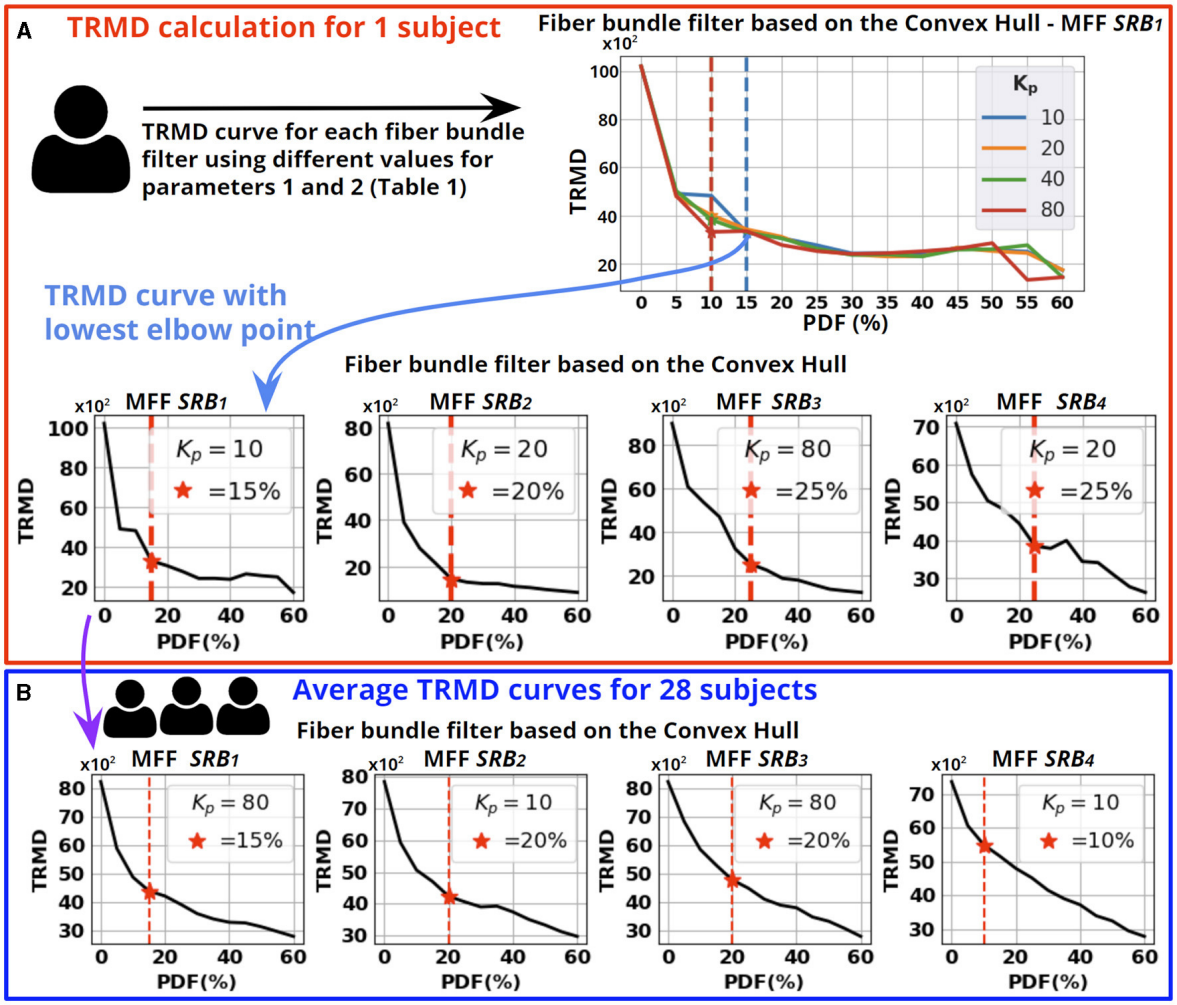
Parameter tuning of the fiber bundle filters. **(A)** Computation of TRMD curves for the fiber bundle filter based on the Convex Hull using a single subject. A TRMD curve is generated for each value of *K*_*p*_, using different PDF values. Then, we selected the TRMD curve with the lowest point at the Elbow point. We show the selected curve for the four MFF *SRB*_*i*_. **(B)** We averaged the selected curves for the 28 subjects to calculate the appropriate parameters for each fiber bundle filter. Each averaged TRMD curve shows the selected PDF (Elbow point shown as a red star) and the selected *K*_*p*_ (statistical mode from the 28 selected curves). The steps described in **(A, B)** were performed for each fiber bundle filter.

Next, we describe the calculation of appropriate parameters for each fiber bundle filter based on the TRMD. First, we compute TRMD curves for each fiber bundle filter using different parameter values (see Table 1) at the individual level (see Figure 7A). For each TRMD curve, we used the Elbow point to choose the best trade-off between reducing the TRMD and increasing the PDF. Then, the curve providing the lowest TRMD on the Elbow point was selected.

**Table 1:** Values used for each parameter of the fiber bundle filters at the individual level.

**Fiber bundle filter**	**Parameter 1 (PDF %)**	**Parameter 2**
Connectivity Patterns	0–60%, step size of 5%	θ_*END*_ (mm) = 5, 8, 10, 12, 15
SSPD	0–60%, step size of 5%	θ_*SSPD*_ (mm) = 5, 8, 10, 12, 15
Fiber Consistency	0–60%, step size of 5%	*K*_*f*_ = 10, 20, 40, 80, 120
Convex Hull	0–60%, step size of 5%	*K*_*p*_ = 10, 20, 40, 80

To perform a parameter tuning for each fiber bundle filter and MFF *SRB*_*i*_, we averaged the corresponding selected curves for the 28 subjects (see Figure 7B). Then, we identified the Elbow of the averaged curves to determine an appropriate PDF. The second parameter of each filter was set as the statistical mode of the selected curves. In Table 2, we show the parameters computed from the 28 subjects for each filter. The automatic detection of Elbow points is described in Supplementary material S4. Supplementary material S5 shows TRMD curves for an individual subject and the averaged TRMD curves.

**Table 2:** Parameters selected for each fiber bundle filter and main fiber fascicle of the segmented representative bundles (MFF *SRB*_*i*_).

**Fiber bundle filter**	**MFF *SRB*_1_**	**MFF *SRB*_2_**	**MFF *SRB*_3_**	**MFF *SRB*_4_**
Connectivity Patterns	*PDF* = 15%	*PDF* = 10%	*PDF* = 15%	*PDF* = 20%
	θ_*END*_ = 8.0	θ_*END*_ = 8.0	θ_*END*_ = 12.0	θ_*END*_ = 10.0
SSPD	*PDF* = 10%	*PDF* = 15%	*PDF* = 15%	*PDF* = 10%
	θ_*SSPD*_ = 5.0	θ_*SSPD*_ = 5.0	θ_*SSPD*_ = 5.0	θ_*SSPD*_ = 5.0
Fiber Consistency	*PDF* = 20%	*PDF* = 15%	*PDF* = 20%	*PDF* = 15%
	*K*_*f*_ = 80	*K*_*f*_ = 120	*K*_*f*_ = 20	*K*_*f*_ = 120
Convex Hull	*PDF* = 15%	*PDF* = 20%	*PDF* = 20%	*PDF* = 10%
	*K*_*p*_ = 80	*K*_*p*_ = 10	*K*_*p*_ = 80	*K*_*p*_ = 10

#### 2.7.2 Test-retest reproducibility analysis in the training set

This section describes the methodology used to select the best fiber bundle filter. Over the subjects' test and retest main fiber fascicles, we applied each fiber bundle filter with parameters from Table 2. The selection of the best filter is based on the best scores in the reproducibility indices (described in Section 2.6).

We also calculated test-retest reproducibility metrics for every possible combination between the four fiber bundle filters. Combining two or more filters means that each fiber bundle filter was applied independently to the main fiber fascicle. Then, we obtained the intersection of shared fibers between every filtered fascicle to produce a final bundle. The results demonstrate an improvement in the test-retest reproducibility indices when combining two or more filters. Nonetheless, the improvement was minimal compared to only applying the filter based on the Convex Hull. Further details are available in Supplementary material S6.

Finally, the best fiber bundle filter was applied to the segmented representative bundles without the identification of the main fiber fascicle, and their test-retest reproducibility was assessed. The results show that filtering also improved the test-retest reproducibility indices in this case. See Supplementary material S7 for more details.

We used the Wilcoxon signed-rank test to determine the statistically significant improvement of the test-retest reproducibility indices after filtering (Wilcoxon, 1945), corrected for multiple comparisons using False Discovery Rate (FDR) (Benjamini and Hochberg, 1995).

#### 2.7.3 Validation set processing

This section evaluates the performance of the best fiber bundle filter using all atlas bundles. The atlas bundles were grouped by shape in the SWM atlas processing, and the parameters of the fiber bundle filter were computed according to the shape of the bundle. Therefore, the parameters of the best filter were set according to the shape of the bundle (see Table 2).

We used the subjects from the validation set and bundles segmented in the 16 subjects with a minimum of 10 fibers. First, we applied the best filter over bundles with and without the identification of the main fiber fascicle. Then, we assessed the improvement of the test-retest reproducibility indices from the filtered fiber bundles.

Additionally, we applied a random filtering of fibers, using the same percentage of fiber discarding of the best fiber bundle filter. The results show that the test-retest reproducibility indices did not change by respect to the non-filtered bundles. This demonstrates that the improvement in reproducibility indices was due to the removal of spurious fibers rather than the decrease in the fiber count of the bundles. Refer to Supplementary material S9for further details.

Again, we used the Wilcoxon signed-rank test to assess the statistically significant improvement of the test-retest reproducibility indices after filtering (Wilcoxon, 1945), corrected for multiple comparisons using FDR (Benjamini and Hochberg, 1995).

### 2.8 Enhanced fiber bundle segmentation applied in the ABIDE-II database

In this section, we applied the enhanced short fiber bundle segmentation in lower-quality data from the ABIDE-II database. In this case, the enhanced segmentation was applied to detect alterations in diffusion-based microstructural indices.

**Figure 8:**
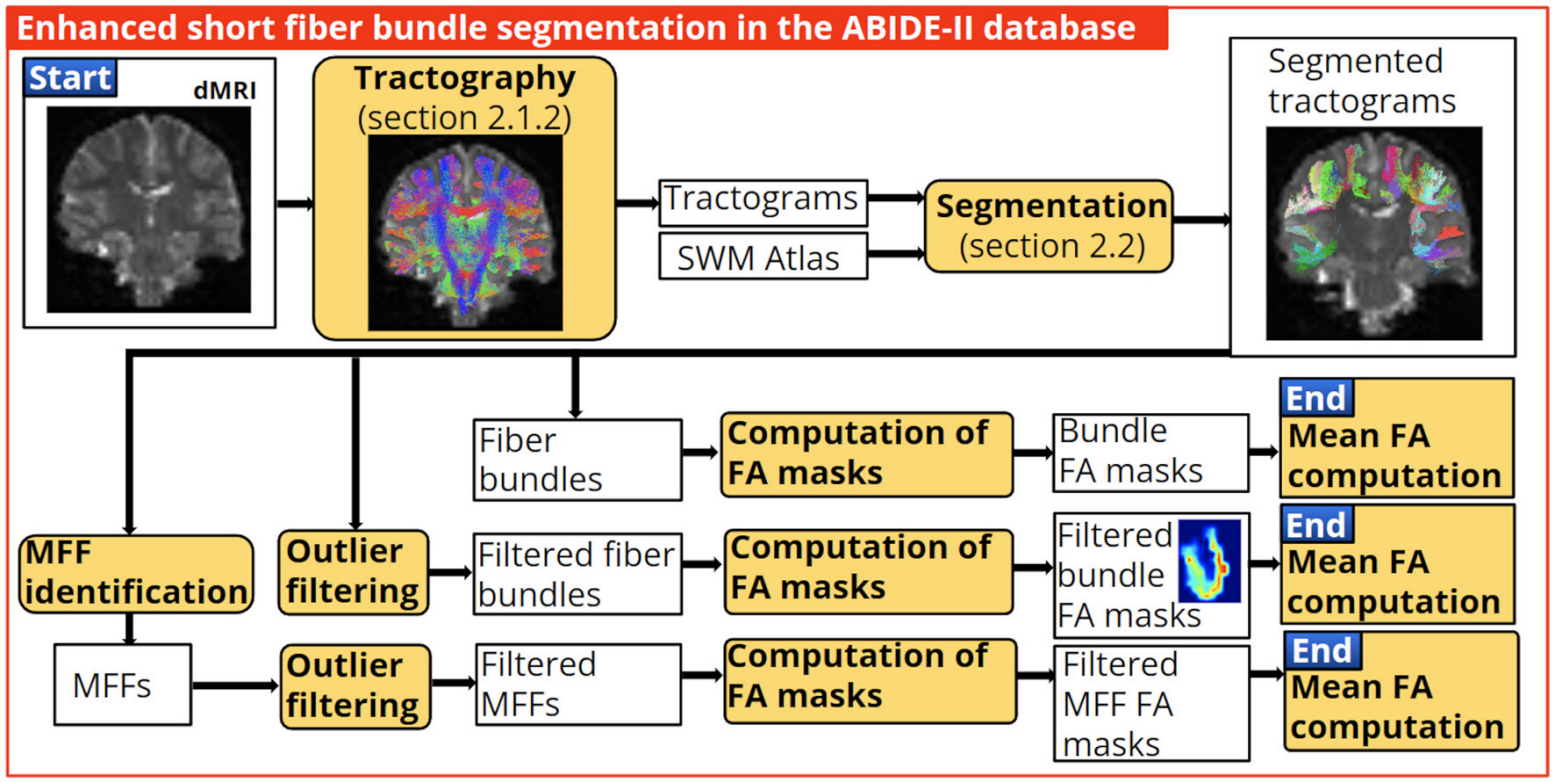
Application of the enhanced short fiber bundle segmentation in the clinical quality database ABIDE-II. We used the identification of the main fiber fascicle and the best fiber bundle filter to obtain well-defined bundles. Then, bundle FA masks were computed, and the bundles' mean FA values were calculated to assess statistically significant differences between control and ASD subjects. Finally, we compared the number of bundles with the significant difference found with the enhanced and without processing segmentation.

We used bundles segmented in all subjects with a minimum of 10 fibers. Then we applied the best fiber bundle filter (with parameters from Table 2), with and without the identification of the main fiber fascicle. Next, a Fractional Anisotropy (FA) mask was calculated for each bundle, and the mean FA value was computed. Subsequently, a two-tailed independent t-test was used to assess the significant difference in the mean FA of the bundles between control and subjects with ASD. Finally, we compared the number of significant bundles found with the enhanced segmentation and the segmentation without processing (see Figure 8). We also present results for the Mean Diffusivity (MD) and the Radial Diffusivity (RD).

The normal distribution of the bundles' mean FA, MD and RD was tested using the Shapiro-Wilk test (Shapiro and Wilk, 1965). For non-normally distributed data, the Mann-Whitney test (Mann and Whitney, 1947) was employed. All statistical tests were conducted with a significance threshold set at a *p*-value < 0.05.

## 3 Results

### 3.1 Results for the test-retest reproducibility analysis

In this section, we present the test-retest reproducibility indices (described in Section 2.6) for the *SRB*_*i*_ of subjects from the training set, processed with the identification of the main fiber fascicle and each fiber bundle filter. To ease the reading of the following sections, we use the notation MFF+Filter_*j*_, which refers to the bundle *SRB*_*i*_ processed with the identification of the main fiber fascicle and further processed with fiber bundle filter *j*. For example, MFF+CH means we processed the segmented bundles with the identification of the main fiber fascicle, and then applied the Convex Hull filter. Supplementary Table S3 summarizes all notations.

Next, we present the mean score for each test-retest reproducibility index (see Table 3). The mean Dice Volumetric Overlap score for the main fiber fascicles shows moderate to relatively good agreement in the volume occupied. The MFF + CH processing had the highest improvement compared to the MFF scores. Also, a significant improvement was found between the MFF and MFF+CH scores for bundles *SRB*_1_, *SRB*_2_ and *SRB*_4_ (*p*-value < 0.05 for each comparison), which demonstrates that the filtering improved the agreement in the volume occupied between test-retest main fiber fascicles.

**Table 3:** Mean scores for each test-retest reproducibility index and *SRB*_*i*_ (mean ± standard deviation), using the fiber bundle filters.

		**MFF**	**MFF + CP**	**MFF + SSPD**	**MFF + FC**	**MFF + CH**
DSC	*SRB*_1_	0.71 ± 0.09	0.71 ± 0.09	0.71 ± 0.10	0.72 ± 0.11	**0.73** **±0.11**
	*SRB*_2_	0.73 ± 0.06	0.75 ± 0.06	0.75 ± 0.06	0.76 ± 0.06	**0.77** **±0.06**
	*SRB*_3_	0.56 ± 0.17	0.56 ± 0.17	0.56 ± 0.18	0.56 ± 0.18	**0.57** **±0.18**
	*SRB*_4_	0.76 ± 0.05	0.76 ± 0.06	0.77 ± 0.05	0.77 ± 0.07	**0.78** **±0.06**
AFD	*SRB*_1_	2.11 ± 0.13	2.13 ± 0.15	2.13 ± 0.14	2.15 ± 0.15	**2.17** **±0.15**
	*SRB*_2_	2.03 ± 0.12	2.05 ± 0.12	2.06 ± 0.11	2.08 ± 0.12	**2.09** **±0.11**
	*SRB*_3_	1.73 ± 0.30	1.73 ± 0.31	1.74 ± 0.31	1.75 ± 0.33	**1.76** **±0.33**
	*SRB*_4_	2.13 ± 0.08	2.13 ± 0.09	2.15 ± 0.08	2.17 ± 0.08	**2.18** **±0.09**
AMD	*SRB*_1_	3.63 ± 0.66	3.56 ± 0.67	3.58 ± 0.69	**3.50** **±0.71**	3.52 ± 0.68
	*SRB*_2_	2.96 ± 0.48	2.86 ± 0.51	2.86 ± 0.52	2.80 ± 0.52	**2.79** **±0.53**
	*SRB*_3_	4.67 ± 1.15	4.62 ± 1.21	4.64 ± 1.23	**4.58** **±1.30**	4.62 ± 1.36
	*SRB*_4_	2.51 ± 0.32	2.43 ± 0.36	2.44 ± 0.33	**2.37** **±0.34**	2.39 ± 0.33
AD	*SRB*_1_	11.23 ± 1.11	10.53 ± 1.24	10.76 ± 1.20	**10.44** **±1.27**	10.60 ± 1.23
	*SRB*_2_	11.17 ± 1.43	10.39 ± 1.62	10.21 ± 1.64	10.17 ± 1.66	**10.02** **±1.73**
	*SRB*_3_	13.00 ± 0.95	11.98 ± 1.11	12.12 ± 1.13	11.84 ± 1.19	**11.56** **±1.16**
	*SRB*_4_	10.73 ± 0.78	**9.32** **±0.87**	10.29 ± 0.86	9.86 ± 0.98	10.20 ± 0.93

The bold values indicate the best score.

The shape of the filtered main fiber fascicles was smoother, as demonstrated by the higher values of the Average Fractal Dimension. Similarly, the MFF+CH processing had the highest improvement compared to MFF scores. In addition, a significant improvement was found between the MFF and MFF+CH scores for the four *SRB*_*i*_ (*p*-value < 0.05 for each comparison), demonstrating the ability to remove spurious fibers, as they constitute one of the main sources of irregularity in the shape of the bundle.

The mean score for the Average Minimum Distance improved when applying the fiber bundle filters. The MFF + FC processing had the highest improvement in bundles *SRB*_1_, *SRB*_3_ and *SRB*_4_, when compared to MFF scores. Significant improvements were found between the MFF and MFF + FC scores for bundles *SRB*_1_ and *SRB*_4_ (*p*-value < 0.05 for each comparison). The MFF+CH processing had the highest improvement in bundle *SRB*_2_, compared to MFF scores (*p*-value < 0.05). The results of the AMD indicate that the application of the fiber bundle filters allowed us to obtain more geometrically similar and compact test-retest fascicles.

The mean score for the Average Distance shows that fibers from filtered test-retest fascicles were spatially closer. The MFF+FC processing had the best improvement in bundle *SRB*_1_, when compared to the MFF scores (*p*-value < 0.05). The MFF+CH processing had the highest improvement in bundles *SRB*_2_ and *SRB*_3_ when compared to MFF scores (*p*-value < 0.05 for each comparison). The MFF+CP processing had the highest improvement in bundle *SRB*_4_ when compared to MFF scores (*p*-value < 0.05). Results for the Average Distance demonstrate the ability of the fiber bundle filters to remove fibers far away from the fascicle's core and generate test-retest fascicles with fibers spatially close to each other.

**Figure 9:**
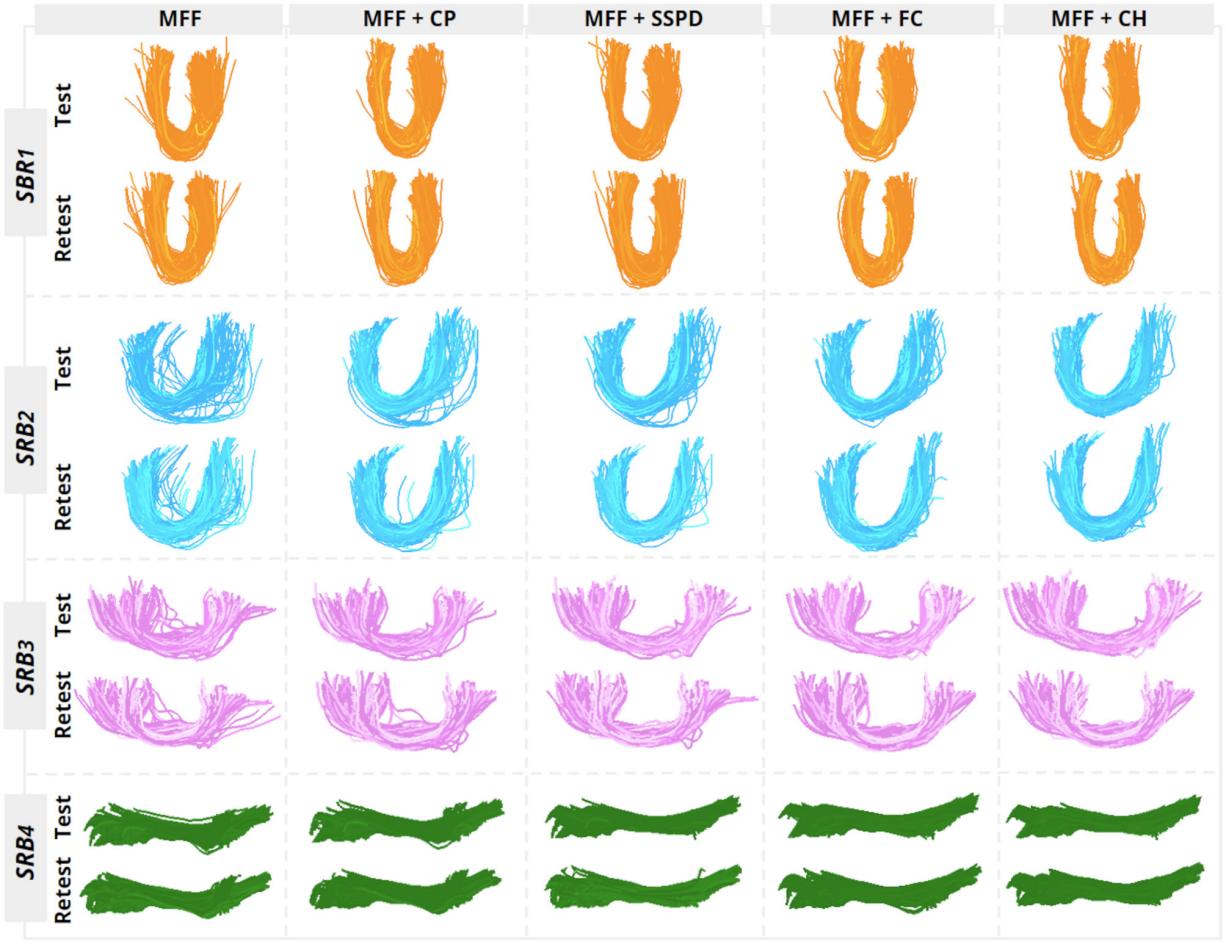
Resulting test-retest main fiber fascicles for subject 143325 of the training set. We show each MFF *SRB*_*i*_ processed with each fiber bundle filter. The filter based on the Convex Hull (fifth column) had the best results, generating rounded bundles with smooth shape and with most isolated fibers removed.

The fiber bundle filter based on the Convex Hull was chosen as the best filter because it had the highest improvement in most test-retest reproducibility indices. In Figure 9, we show each *SRB*_*i*_ of subject 143325, where the fiber bundle filter based on the Convex Hull removed most isolated fibers, providing well-defined fascicles and improving their test-retest similarity. Finally, the identification of the main fiber fascicle removed an average percentage of 51.6 ± 17.16% fibers from all *SRB*_*i*_.

To analyze the configuration of rejected fibers, we applied the QuickBundles clustering (Garyfallidis et al., 2012) to segmented bundles of the training set from the HCP database (28 subjects). In Supplementary Figure S18 we show histogram plots of the mean cluster size (number of fibers) and frequency for segmented representative bundles, filtered bundles and the rejected fibers. The clustering of segmented bundles generates clusters with a frequency decreasing from 100 for the range [1–6] fibers, to around 20 for the range [91–96] clusters. Furthermore, filtered bundles present fewer small clusters, with < 50 clusters with an average size in the range [1–6]. On the other side, rejected fibers were clustered into a large number of small clusters, with a frequency ranging from 190 to 300 clusters for the same size range. These results show that in general, the filtering removes spurious fibers. However, it may exist some atlas bundles with one or more subpopulations of fibers.

Additionally, in the Supplementary material, we included two figures showing representative fibers of the rejected and accepted fibers of each filter and MFF, for the U-shaped (Supplementary Figure S19) and the open *U*-shape (Supplementary Figure S20) representative bundles. In general, rejected fibers are similar for the four filters, presenting an irregular shape and positioned far away from the core of the bundle. It can be observed that the FC and CH filters discard more complex-shaped fibers than the other two filters, generating cleaner bundles. This may be because these filters are not only based on a distance measure but also consider in some way the density of the bundles. Respecting the MFF, is it especially useful for bundles with an open *U*-shape, such as the one illustrated in Supplementary Figure S20, where noisy fibers in the middle of the bundle, differing from the main bundle shape, are removed more efficiently.

### 3.2 Results for the test-retest reproducibility evaluation

This section presents results for the test-retest reproducibility indices using all atlas bundles. We used fiber bundles segmented in the 16 subjects from the validation set (and in both test and retest acquisitions) with a minimum of 10 fibers. Using these criteria, we obtained 462 fiber bundles per subject and a total of 7,392 (16 × 462) bundles. We applied the identification of the main fiber fascicle and the fiber bundle filter based on the Convex Hull. For the resulting bundles of each processing, we computed the mean score of the test-retest reproducibility indices (see Table 4), which provides an overall view of the performance of the filter based on the Convex Hull.

**Table 4:** Mean scores for segmented bundles in the validation set, with and without processing.

**Test-retest reproducibility indice**	**NP**	**CH**	**MFF**	**MFF + CH**
Dice Volumetric Overlap	0.70 ± 0.11	**0.74** **±0.12**	0.66 ± 0.15	**0.68** **±0.16**
Average Fractal Dimension (AFD)	2.01 + 0.21	**2.11** **±0.21**	1.92 ± 0.29	**1.99** **±0.29**
Average Minimum Distance (AMD)	3.68 ± 0.91	**3.57** **±1.01**	3.88 ± 1.24	**3.77** **±1.41**
Average Distance (AD)	15.70 ± 2.91	**14.05** **±3.02**	12.53 ± 2.12	**11.35** **±2.28**

The Dice Volumetric Overlap shows the mean DSC. The AD and AMD are in *mm*. Bold values indicate an improvement of the score after applying the filter based on the Convex Hull over NP bundles and main fibers fascicles.

We use the label “No Processed” (NP) to refer to segmented fiber bundles with neither the identification of the main fiber fascicle nor the fiber bundle filter based on the Convex Hull processing. Also, the label CH refers to segmented fiber bundles processed only with the Convex Hull filter.

First, we present results for CH bundles compared to NP bundles. We obtained a higher score for the Dice Volumetric Overlap in filtered bundles. Therefore, filtered test-retest bundles had a higher agreement in the volume occupied. Also, CH bundles had a smoother and more regular shape than NP bundles, as demonstrated by the higher score of the AFD. Concerning the spatial positioning of the fibers, the results of the AMD score demonstrate that the filtered bundles had fewer isolated fibers than NP bundles. Finally, CH bundles had a lower AD score than NP bundles, which means that filtered test-retest bundles had fibers spatially closer to each other.

Next, we describe the results for the main fiber fascicles, with and without filtering. The reproducibility scores of the main fiber fascicles improved when applying the filtering (MFF+CH column of Table 4) for all indices. After filtering, we obtained a higher score for the Dice Volumetric Overlap and AFD. Therefore, filtered main fiber fascicles were in greater agreement in the volume occupied with smoother shapes than MFF bundles. Also, lower scores for the AD and AMD were found for the MFF+CH, which means that test and retest main fibers fascicles were more compact and with fewer isolated fibers after filtering. Finally, the identification of the main fiber fascicle removed an average percentage of 53.1 ± 19.5% fibers from segmented bundles.

Supplementary Table S5 shows the number of fiber bundles with a significant improvement over the reproducibility indices when applying the filter based on the Convex Hull. By solely applying the filtering, we improved the test-retest reproducibility indices in over 300 short fiber bundles. Likewise, by applying the filtering to the main fiber fascicles, we obtained improved test-retest reproducibility indices in over 300 bundles. In Supplementary material S12 we show figures of filtered fiber bundles.

### 3.3 Results for the ABIDE-II database

We used bundles segmented in all subjects with a minimum of 10 fibers, resulting in 422 bundles per subject. In Table 5, we show the number of bundles with statistically significant difference (uncorrected *p*-value < 0.05) in the mean FA, MD and RD, between control and ASD subjects. We found a higher number of bundles using the filter based on the Convex Hull.

**Table 5:** Number of bundles with significant differences in diffusion-based microstructural indices between control and ASD subjects (uncorrected *p*-value < 0.05).

	**NP**	**CH**	**MFF + CH**
FA	2	4	8
MD	15	13	20
RD	7	13	17

A higher number of significant bundles were found with the identification of the main fiber fascicle and filter based on the Convex Hull.

**Figure 10:**
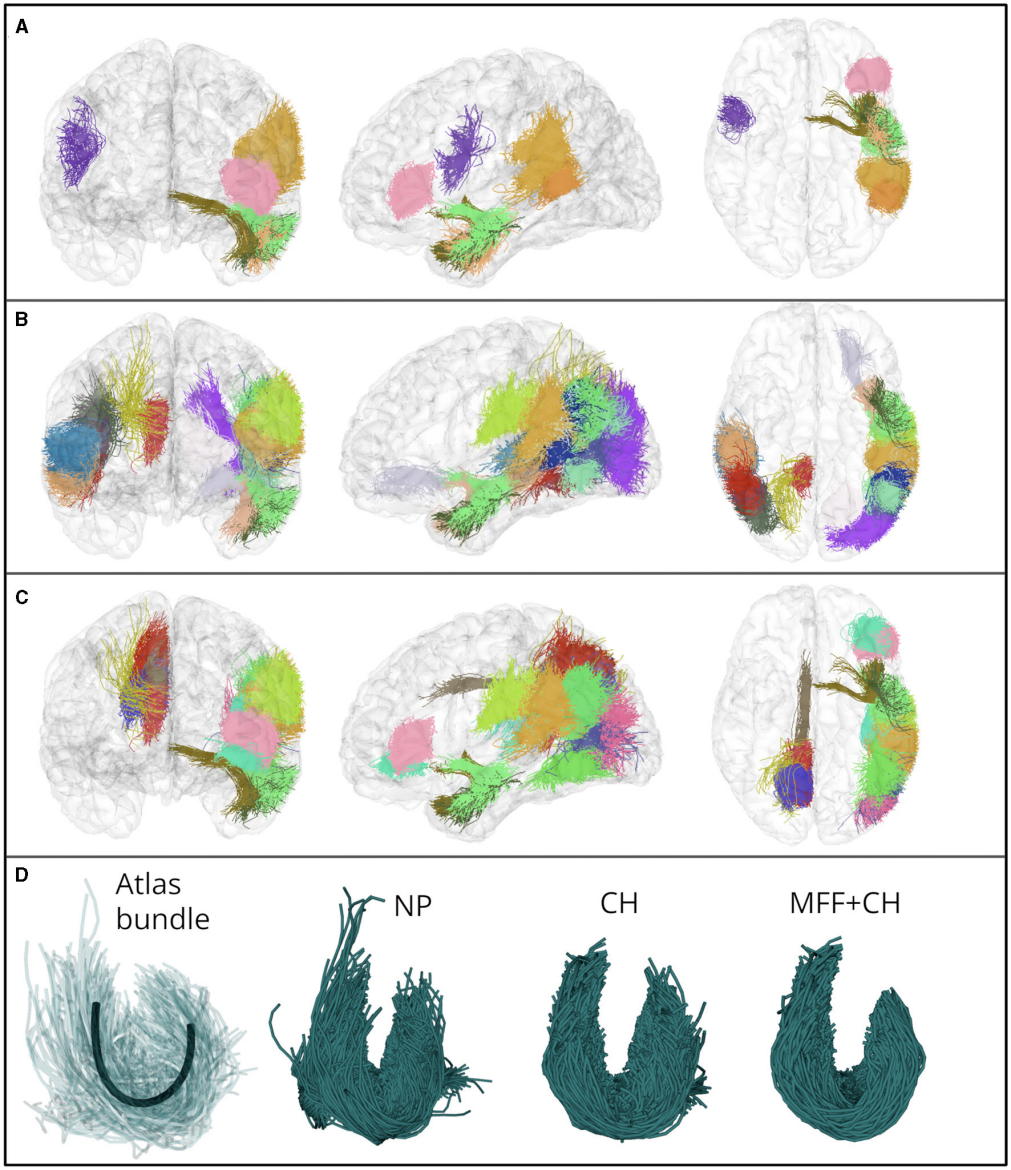
**(A–C)** Show bundles with significant differences in FA, MD and RD mean values, between control and ASD subjects. The bundles were processed with the identification of the main fiber fascicle and the filter based on the Convex Hull. **(A)** Eight fascicles with significant difference in the mean FA. **(B)** Twenty fascicles with significant difference in the mean MD. **(C)** Seventeen fascicles with significant difference in the mean RD. **(D)** Atlas bundle connecting the superior temporal and supramarginal gyri (centroid is shown in black), and the segmented bundle in a control subject. The filter based on the Convex Hull allowed us to obtain well-defined bundles in a lower quality database.

In Figure 10A, we show the eight bundles with significant differences in mean FA between controls and subjects with ASD. These bundles were processed with the identification of the main fiber fascicle and the filter based on the Convex Hull. Likewise, we show the twenty bundles with significant MD differences in Figure 10B and the seventeen bundles with significant differences in the RD in Figure 10C. Additionally, in Figure 10D, we show the segmented bundle of a control subject. It can be seen that the identification of the main fiber fascicle and the filter based on the Convex Hull obtained well-defined bundles with fewer spurious fibers than NP bundles, in a lower-quality database.

Supplementary Tables S7–S15 provide the bundles' mean FA, MD, and RD values averaged across subjects. Also, the uncorrected *p*-value and the Cohen's d are shown for each bundle. Decreased FA, increased MD, and increased RD were found in subjects with ASD. Also, Supplementary Figures S27–S29 show more filtered bundles from control subjects. After applying the FDR correction for multiple comparisons, no significant differences were found in any of the segmentation results, regardless of whether fiber filtering was applied or not. This is due to the high number of comparisons performed, which potentially increases the likelihood of missing genuinely significant findings. Also, bundles found to be significant without FDR correction had a medium to large effect size (Cohen's *d*).

To evaluate whether the filter based on the CH is also the best performing for the ABIDE-II database we performed a test with a small subsample of 5 control subjects randomly chosen from the ABIDE-II database, with a second diffusion MRI image (acquired in the same session). We applied the four fiber filters to the MFF bundles and calculated test-retest reproducibility indices. In Supplementary Table S16, we list the mean score for each index. Similar to the HCP database results, the fiber bundle filter based on the Convex Hull had the best improvement in most indices.

Furthermore, in Supplementary Figure S30, we show bundles for one subject from each database (ABIDE-II and HCP). In general, bundle shapes and spurious fibers are similar for both databases. Nonetheless, we noticed that some bundles from the ABIDE-II database were more irregular than bundles from the HCP database, which could be explained by the lower quality of the data (see bundle *SRB*_2_ of Supplementary Figure S30). Furthermore, in Supplementary Figure S31, a whole-brain tractogram and 100 randomly selected bundles from a subject of the HCP database and a subject from the ABIDE-II database are shown. It can be seen that the filtering produced more compact short fiber bundles for both subjects. Also, the fiber bundles present in general similar shape, even though the fiber bundles are more noisy and dispersed for the ABIDE-II database subject.

Additionally, we performed a quick experiment to test the validity of using the filters' parameters tuned with the HCP database without any further filter optimization on the ABIDE-II database. For that, we analyzed the same small subsample of five control subjects from the ABIDE-II database employed in the previous test. Then, we applied the parameter tuning based on the Test-Retest Maximum Distance (TRMD), described in Section 2.7.1. Results show that the parameters obtained for the ABIDE-II subjects are very similar to those calculated using the HCP database for bundles *SRB*_1_ and *SRB*_2_, representing the shape of ~80% of the atlas bundles (see Supplementary Figure S32). Furthermore, we calculated the mean FA, MD, and RD values for the filtered bundles of the ABIDE-II database using the two sets of optimal parameters. Supplementary Tables S17, S18 list these values for the segmented representative bundles, where it can be seen that the results are quite similar, with slight differences in a few bundles.

Finally, Supplementary Tables S19–S21 show the mean and standard deviation of FA, MD, and RD metrics before and after filtering with CH and MFF + CH. Furthermore, the difference between the mean metrics between control and ASD subjects before and after filtering are also included. Results show that in general, the filtering increased the difference between control and ASD subjects.

## 4 Discussion

This paper proposes several tools to better study the SWM fiber bundles. Our work consisted of implementing and validating four fiber bundle filters to remove spurious fibers. Furthermore, we define a methodology to identify the main fiber fascicle, which allows us to disregard fibers whose shape differs from the main atlas bundle shape, enabling us to obtain well-defined bundles. Our results show an improvement in several test-retest reproducibility indices from short fiber bundles of the HCP database.

Also, the filter application allowed us to improve the quality of the short fiber bundles from a lower-quality database (ABIDE-II). We demonstrated the relevance of filtering by improving the sensitivity to alterations in diffusion-based microstructural indices (FA, MD, and RD) between control and ASD subjects. We found a large number of bundles with significant differences in microestructural indices using fiber bundle filtering. Furthermore, these bundles were predominantly located in the parietal and temporal lobes, consistent with the findings in the existing literature. We found a decrease in FA and an increase in MD and RD in subjects with ASD compared to controls. As discussed in Section 4.2, our results align with previous reports on the topic, where the same trend of microstructural alterations has been reported for the subjects with ASD.

### 4.1 Enhanced short fiber bundle segmentation in the HCP database

We enhanced the short fiber bundle segmentation in two ways. First, we used the atlas bundle centroids to identify the main fiber fascicle. Second, we designed and implemented four fiber bundle filters to remove spurious fibers. The identification of the main fiber fascicle allowed us to obtain well-defined bundles. However, this identification step may depend on the research objective. If the study focuses on performing a detailed mapping of the *U*-fiber shapes, then it could be beneficial to use main fiber fascicles. However, we suggest omitting this step if high cortical coverage is needed. Furthermore, future work could improve the identification process by employing several atlas bundle centroids, allowing the description of other shapes that may exist within the atlas bundle.

Fiber bundle filters were applied to remove spurious fibers. First, the HCP database was split into two groups of subjects: the training and validation set. Next, we describe the main findings of the training set. Table 2 presents the recommended values for the filter parameters according to the shape of the bundle. These parameters were used to filter segmented representative bundles. Our results show that the best filter is the Convex Hull, which provides the highest improvement in most of the test-retest reproducibility indices.

Test and retest bundles, processed with the filter based on the Convex Hull, had a higher agreement on the volume occupied, a smoother shape, and fewer isolated fibers than the unprocessed bundles. The test-retest reproducibility indices showed minimal improvement when two or more fiber bundle filters were combined. Furthermore, we showed that the filter based on the Convex Hull improved the test-retest reproducibility with or without the identification of the main fiber fascicle.

The superior performance of the fiber bundle filter based on the Convex Hull may be attributed to its topological properties. The Convex Hull's envelope offers a straightforward way of identifying spurious fibers with noisy trajectory, as they are likely to provide a vertex to the envelope. This inherent topological advantage allowed us to efficiently identify and isolate spurious fibers, contributing to the filter's effectiveness.

In the validation set, we evaluated the filter based on the Convex Hull using 462 bundles per subject. We found a significant improvement in the test-retest reproducibility indices in more than 300 short fiber bundles. The application of the main fiber fascicle and the filtered test-retest bundles had higher agreement in the occupied volume, a smoother shape, and fewer isolated fibers than the unprocessed bundles.

Although an alternative approach could be filtering out spurious fibers from the atlas bundles and then performing fiber bundle segmentation, we opted to apply the filtering process directly to the segmented bundles. This decision was based on our previous experience, where we observed that the proposed pipeline yielded better results.

### 4.2 Results from the ABIDE-II database

Few studies investigate SWM's structural connectivity in individuals with Autism Spectrum Disorder (ASD). In the following, we describe the most important findings of these studies and compare them with our results. Sundaram et al. (2008) reported a decrease in FA in short-range fibers of the frontal lobe, using subjects with ASD aged 4.8 ± 2.4 years old. Shukla et al. (2011) reported a decrease in FA and an increase in MD and RD in short association fibers of the frontal lobe from subjects with ASD between 9 and 18 years old. Additionally, they found an increase in MD and RD in short fibers of the parietal and temporal lobes. On the other hand, d'Albis et al. (2018) used adult subjects with ASD and reported a decrease in structural connectivity in 13 short fiber bundles from the temporal-parietal-frontal lobes.

Hong et al. (2018) used cortical surface and microstructural indices to quantify SWM alterations in 53 subjects with ASD. They reported a decrease in FA and an increase in MD and RD in the medial parietal and lateral temporo-parietal regions in subjects with ASD. Furthermore, reduced FA and increased MD were observed in the precuneus and posterior cingulate regions in subjects with ASD. Finally, Bletsch et al. (2020) used 92 adult subjects with ASD aged 18–52 years. Individuals with ASD showed reduced FA in the SWM of the right temporal lobe and the left lateral orbitofrontal cortex. In addition, they observed an increase in MD in the SWM of the orbitofrontal cortex, pars triangularis, left fusiform, and inferior temporal regions.

Comparison between studies is challenging due to the different methodologies, ages of the subjects, and criteria used to define the SWM. Nevertheless, our results are consistent with other works, where FA decreases and MD and RD increase in the SWM of individuals with ASD. See, for example, microstructural alterations (Supplementary Tables S7–S15). Overall, the brain regions most affected by ASD reported in the literature correspond to the frontal, parietal, and temporal lobes. Using our enhanced segmentation, our work identified bundles with decreased FA in the superior temporal, middle temporal, and supramarginal regions (see Supplementary Table S9). In contrast, the unprocessed segmentation only identified two bundles with a significant difference in FA (see Supplementary Table S7).

Unprocessed and enhanced segmentation allowed us to identify bundles with significant differences in MD. However, enhanced segmentation allowed us to obtain a greater number of significant bundles located in the inferior parietal (Supplementary Table S11) and the middle temporal gyri (Supplementary Table S12). Finally, the enhanced segmentation allowed us to identify a higher number of bundles with significant differences in the RD, located in the precuneus, superior temporal, inferior parietal, and supramarginal giry (Supplementary Tables S14, S15). Overall, the areas most affected in our study correspond to the parietal and temporal lobes, which is consistent with the literature reports. An increase in the number of significant short fiber bundles offers a broader scope for uncovering correlations with clinical manifestations of ASD, such as social awareness or executive functioning (d'Albis et al., 2018). This processing can facilitate a deeper understanding and more precise characterization of cognitive profiles and SWM integrity among individuals with ASD or other diseases.

### 4.3 Limitations and future work

Limitations and future work are summarized below. Our study used a limited sample of 44 subjects from the HCP database (Glasser et al., 2013), and 44 subjects from the ABIDE-II database (Martino et al., 2017), further validation of the developed tools could be performed using more subjects and databases of different quality, such as the Parkinson's progression markers initiative (PPMI) (Marek et al., 2011).

Due to the high inter-subject variability of the short bundles, other parameter tuning strategies could be employed for the filter based on the Convex Hull. For instance, the leverage of statistical analysis or machine learning to automatically fit the parameters of the filter based on the subject's unique bundle features. Another area of improvement in our work is the utilization of fixed segmentation thresholds. Modifying these thresholds could enhance the detection of short fiber bundles with the trade-off of segmenting a higher proportion of spurious fibers. Future work could include an analysis of the performance of the fiber bundle filter based on the Convex Hull when increasing the segmentation thresholds. Also, new tractography algorithms dedicated to the reconstruction of the short connections could be used (Shastin et al., 2022).

The proposed processing does not reassign rejected fibers to other SWM bundles. As it is shown in the Supplementary Figure S18, most of the rejected fibers constitute noisy fibers rather than different subpopulations of fibers. Nonetheless, a reassignment method could be integrated in future work to avoid removing valid streamlines. Also, a more detailed SWM bundle atlas could improve the representation of different fiber populations for high quality data.

This work aimed to evaluate the performance of different SWM fiber bundle filters and determine suggested parameters based on the HCP database, that could be used in other databases. Nevertheless, the two databases used differ vastly in male/female ratio as well as age and data quality. The sample employed from the HCP database is composed of subjects aged 22–25 years old (13 males, 31 females), with high-quality diffusion MRI data (270 total directions, three b-values, and 1.25 *mm* isotropic voxels). On the other hand, the sample from the ABIDE-II database is composed of control subjects (21 male, one female; 9.8 ± 3.6 years old) and Autism Spectrum Disorder (ASD) patients (21 male, one female; 9.8 ± 5.6 years old), with low-quality diffusion MRI data (64 directions, one b-value, and 3 *mm* isotropic voxels). Despite this asymmetry between databases, the parameter tuning in the ABIDE-II subsample (see Supplementary Figure S32) was quite similar to the HCP tuning in most bundles. However, future work could perform an analysis of the filtering using different data quality databases and tractography algorithms to evaluate the robustness of the filters and parameter tuning.

Finally, we limited our work to four test-retest reproducibility indices. Three of them have been previously used to quantify the similarity between fiber bundles: Dice Volumetric Overlap (Bertò et al., 2021; Schilling et al., 2021a), Average Distance (Guevara et al., 2012), and Average Minimum Distance (Schilling et al., 2021a). Furthermore, in our work, we proposed the Average Fractal Dimension (Bertò et al., 2021) as a measure of the average smoothness between the test and the retest bundles. We used indices that best fit our research objectives. Nonetheless, several other proposed indices in the literature, such as the relative difference of the mean FA (Zhang et al., 2019), the Intra-class correlation coefficient (Boukadi et al., 2019) or the volume overreach (Maier-Hein et al., 2017) can be used in future work.

## 5 Conclusion

In conclusion, the fiber bundle filter based on the Convex Hull significantly improved the test-retest reproducibility indices of the short fiber bundles. Our results show that we can identify well-defined short bundles with a regular shape. Our enhanced SWM segmentation could be beneficial in several research lines, such as the study of the regional organization of short fibers (Pron et al., 2020; Guevara et al., 2022), cortical parcellation (López-López et al., 2020), or the creation of SWM atlas (Zhang et al., 2018; Román et al., 2022). Our improvements are relevant to make the bundle segmentation method of Vázquez et al. (2019) or other segmentation algorithms more sensitive to alterations in the SWM diffusion-based microstructural indices. The results derived from the ABIDE-II database provide substantial support for this assertion. We have shown that the filter based on the Convex Hull increased the number of bundles with significant differences in the FA, MD, and RD between controls and subjects with ASD. Notably, the location of the bundles identified with significant differences is consistent with prior research in the field.

## Data availability statement

Publicly available datasets were analyzed in this study. This data can be found at: HCP Young Adult, 1200 Subjects Data Release: https://www.humanconnectome.org/study/hcp-young-adult, ABIDE II database: https://fcon_1000.projects.nitrc.org/indi/abide/abide_II.html. The code of fiber bundle filters and the main fascicle identification, as well as the example datasets are available at: https://github.com/cmendosanchez/EnhancedSWM.

## Ethics statement

The studies involving humans were approved by WUMinn Consortium, NYU Langone Medical Center. The studies were conducted in accordance with the local legislation and institutional requirements. Written informed consent for participation was not required from the participants or the participants' legal guardians/next of kin in accordance with the national legislation and institutional requirements.

## Author contributions

CM: Conceptualization, Methodology, Writing – original draft, Writing – review & editing, Investigation, Software, Visualization. CR: Software, Writing – review & editing, Resources. J-FM: Writing – review & editing, Conceptualization. CH: Writing – review & editing, Conceptualization, Methodology, Resources, Supervision, Writing – original draft. PG: Conceptualization, Methodology, Resources, Supervision, Writing – original draft, Writing – review & editing, Funding acquisition, Project administration.
