# Supplementary material for: Short fiber bundle filtering and test-retest reproducibility of the Superficial White Matter
Source: Front Neurosci. 2024 Apr 26;18:1394681. doi: 10.3389/fnins.2024.1394681 (PMC11088237; doi:10.3389/fnins.2024.1394681)
Supplement: Supplementary file 1 [file Data_Sheet_1.PDF]

Supplementary File

S1 SWM ATLAS PROCESSING

S1.1 Alignment between two fibers

To focus only on the shape differences between two fibers, we perform an alignment based on their central points. Given two fibers A and B, the alignment consists of two steps. First, we center both fibers using their central point. This is performed separately for each fiber by subtracting the coordinates of the central point to the corresponding coordinates of every other fiber point. Thus, the centered fibers have a central point with coordinates (x,y,z) equal to (0,0,0). After centering fibers A and B, we denote them A_c and B_c, respectively (see Fig. S1-A).

Second, we apply an optimal 3D rotation to fiber B_c such that its geometry maximally overlaps with fiber A_c (see Fig. S1-B). To compute the optimal rotation values, we used *Streamline-based Linear Registration* (Garyfallidis et al., 2015). In short, this registration method performs an overlap between a static and a moving set of streamlines. For this purpose, it applies a linear transformation to the moving set of streamlines such that a cost function is minimum, using a L-BFGS-B optimizer (Morales and Nocedal, 2011). It is possible to apply a rigid or affine registration, however, we only computed the 3D optimal rotation values as both fibers were already centered by their central point. After applying the optimal rotation to fiber B_c, we denote it as B_{cr} (**c**entered and **r**otated). Finally, we call the application of both steps *alignment of fiber B to fiber A*.

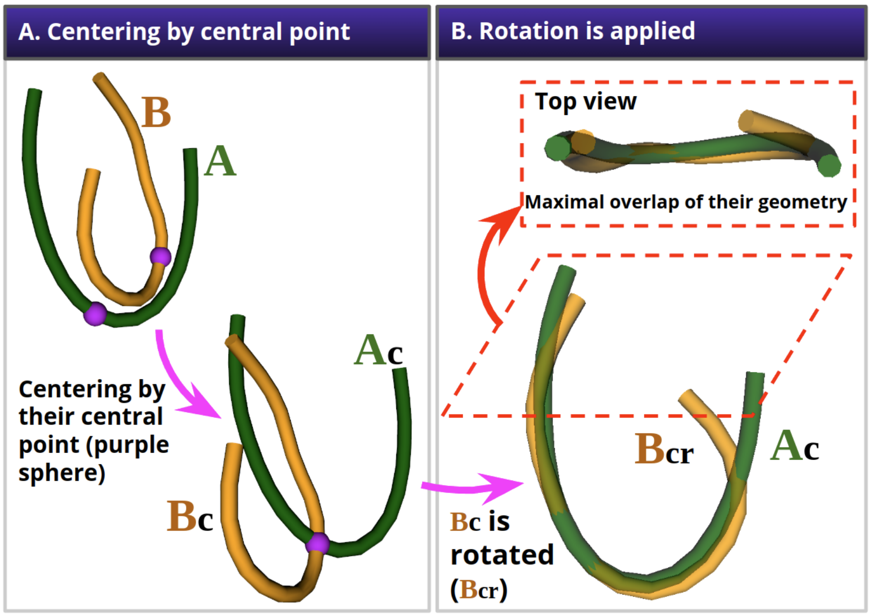

Figure S1. (A) Fibers A (green) and B (orange) are aligned by their central point. After centering, we denote fibers A and B as A_c and B_c, respectively. (B) An optimal rotation is applied to fiber B_c in order to maximally overlap its geometry to fiber A_c. The centered and rotated fiber B is denoted B_{cr}.

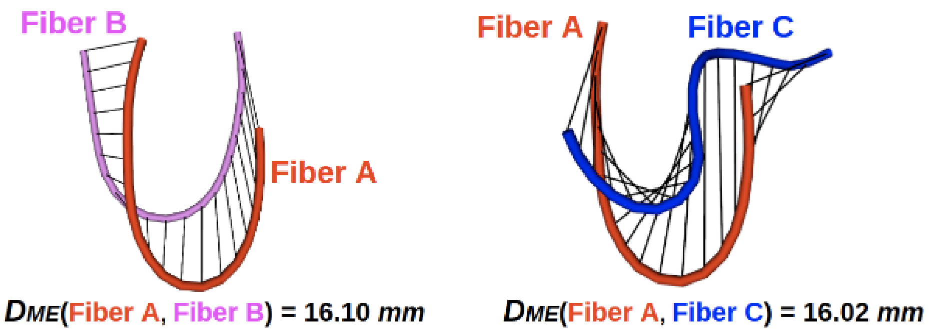

Figure S2. The shape of fibers A and B is more similar than the shape of fibers A and C. However, the D_{ME} distance indicates a higher similarity between fibers A and C due to fiber C being spatially closer to fiber A. By performing an alignment between fibers, we can remove this effect.

S1.2 Fiber distance measure D_{SHAPE}

Once two fibers are aligned, we aim to quantify the similarity between their shape. The distance D_{ME} has been successfully used to study short fibers (Guevara et al., 2017; Román et al., 2022). However, this metric only provides a maximal Euclidean distance between two fibers, making it difficult to identify subtle differences between their trajectory (see Fig. S2).

We propose a new fiber distance measure based on the D_{ME} distance, called D_{SHAPE}, which penalizes the maximum angular aperture between the corresponding fiber linear segments. Given two aligned fibers A_c and B_{cr}, the distance D_{SHAPE} is computed as follows. First, for each fiber, we compute a 3D tangent vector at each fiber point employing finite differences. Next, we calculate the term f_{SHAPE} in Eq. S1, which quantifies the maximum angular aperture between the corresponding tangent vectors of the two aligned fibers (see Fig. S3).

$$f_{SHAPE}(A_c, B_{cr}) = \max_i(\arccos(\frac{a_i \cdot b_i}{\|a_i\| \, \|b_i\|}) \tag{S1}$$

where, a_i and b_i are corresponding tangent vectors of fibers A_c and B_{cr}, respectively. The term f_{SHAPE} ranges from 0 (all vectors are parallel) to π radians (at least one pair of vectors are anti-parallel). Then, the penalization term L_{SHAPE} is calculated in Eq. S2:

$$L_{SHAPE}(A_c, B_{cr}) = 1 + \frac{f_{SHAPE}(A_c, B_{cr})}{\pi} \tag{S2}$$

Finally, the D_{SHAPE} is defined in Eq. S3:

$$D_{SHAPE}(A_c, B_{cr}) = D_{ME}(A_c, B_{cr}) \times L_{SHAPE}(A_c, B_{cr}) \tag{S3}$$

The D_{SHAPE} is simply the D_{ME} distance when all vectors are parallel ($f_{SHAPE} = 0$; $L_{SHAPE} = 1$) and $2 \times D_{ME}$ when fibers have at least one pair of corresponding vectors with opposite directions ($f_{SHAPE} = \pi$; $L_{SHAPE} = 2$). In this paper, the distance D_{SHAPE} is calculated only in a pair of aligned fibers. Finally, it is worth mentioning that the direction of the tangent vectors might change depending on how the fibers are stored on memory. To perform the calculation of the D_{SHAPE} distance between two fibers, we first verified the orientation of the fibers, and realigned them if needed. A detailed description of this processing

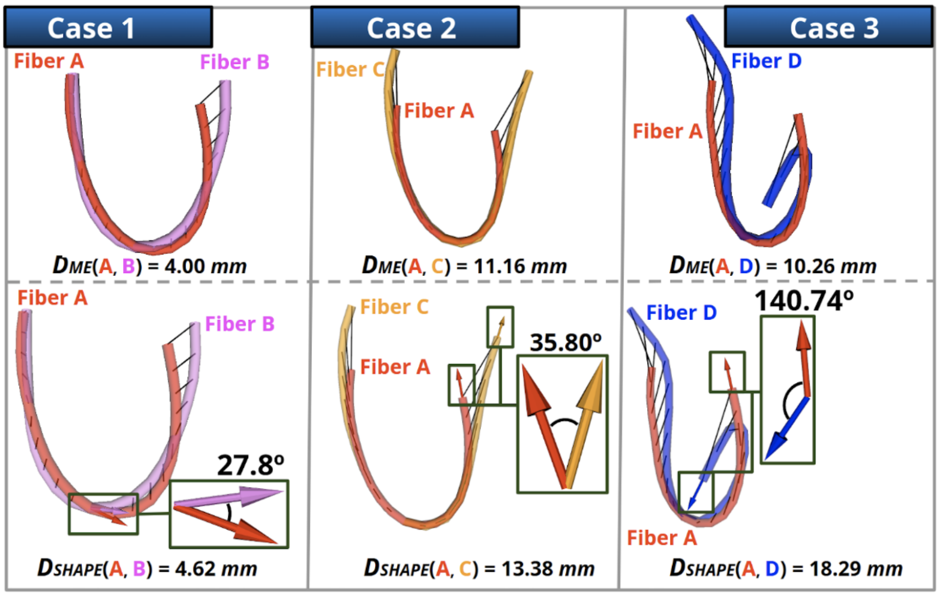

Figure S3. Example of the D_{SHAPE} distance applied to three different cases of aligned fibers. Case 1: two fibers with similar shape, the D_{ME} and D_{SHAPE} distance are low. Case 2: two fibers with overall similar shape, the D_{SHAPE} distance is slightly higher than the D_{ME} due to the L_{SHAPE} penalization term. Case 3: two fibers with different shapes, the D_{SHAPE} distance is much higher due to the high angular difference between their trajectory. Furthermore, the D_{SHAPE} distance enables a better differentiation between cases 2 and 3.

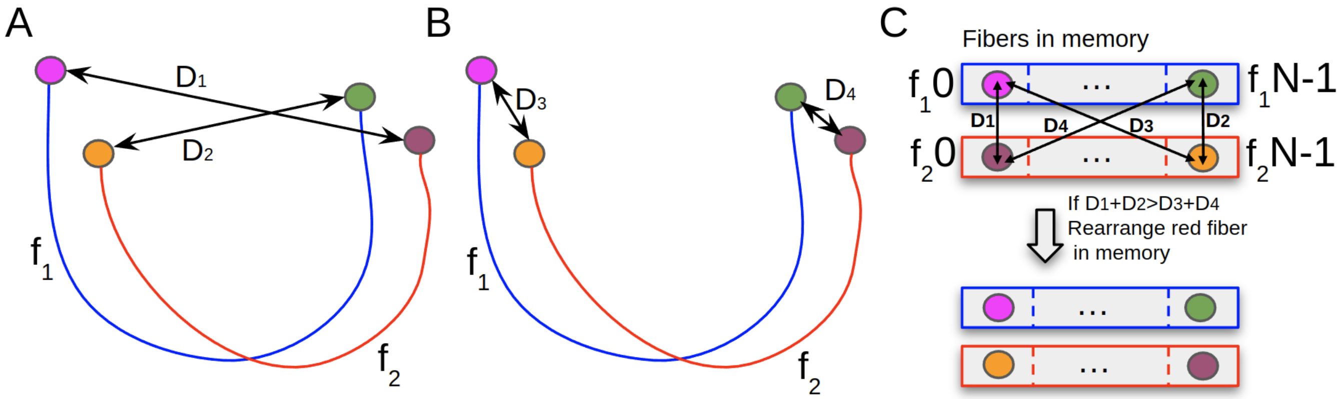

Figure S4. Illustration of the point reorientation of one fiber (f_2) that presents the opposite orientation in memory to another fiber (f_1). First, we calculate the Euclidean distances D_1 and D_2 between the fiber end points for direct comparison ($f_1 0$ — $f_2 0$ and $f_1 N$-1 — $f_2 N$-1), as shown in (A). Next, we calculate the Euclidean distances D_3 and D_4 between the fiber end points for inverse comparison ($f_1 0$ — $f_2 N$-1 and $f_1 N$-1 — $f_2 0$), as shown in (B). To evaluate the orientation of the fibers, the sum of distances D_1 and D_2 is compared with the sum of distances D_3 and D_4. If $D_1 + D_2 > D_3 + D_4$, the fibers have opposite orientations, and a point rearrangement in memory is required for one fiber, f_2 in this case. (C) Schematic of the fiber rearrangement in memory. N is the number of points of each fiber.

is included in Fig. S4. The reorientation is based on the Euclidean distance between their end points to ensure that the aligned fibers had tangent vectors following the same direction of flow.

S1.3 Atlas bundle centroids calculation

This section aims to calculate an adequate centroid for each atlas bundle. We propose an iterative algorithm that includes fiber length, shape and position. The algorithm is made up of the following steps (see Fig. S5). ***Step 1***: Filtering by fiber length. ***Step 2***: Filtering by fiber shape. ***Step 3***: Identification of a fiber in the middle of the bundle (fiber f_M). ***Step 4***: Alignment to fiber f_M and centroid computation. If the resulting centroid's length is less than the average fiber length of the input atlas bundle, then we recalculate every step using a lower threshold for Step 1. Otherwise, the algorithm is terminated. Next, we explain each step in more detail.

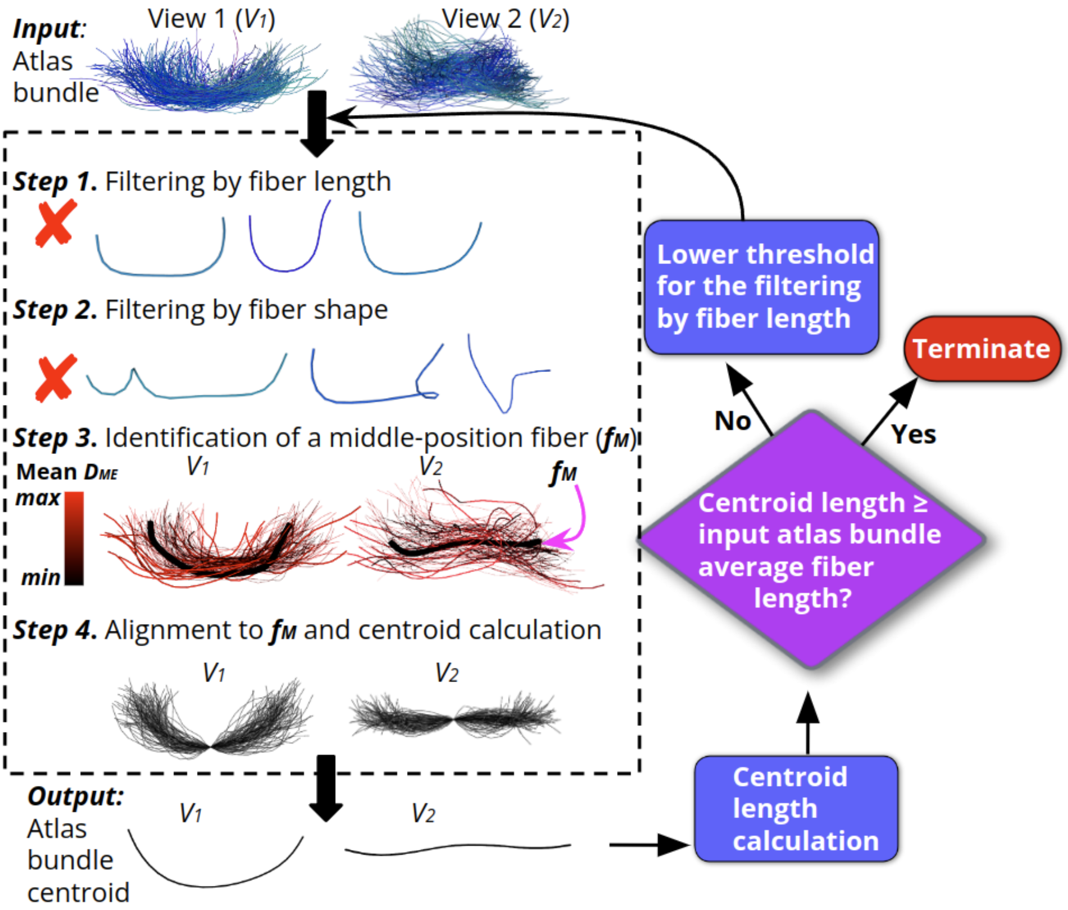

Figure S5. The proposed method for the calculation of an atlas bundle centroid. We show two views of an input atlas bundle (V1 and V2). **Step 1:** Fibers too short to reliably describe the main atlas bundle shape are removed. **Step 2**: Fibers with noisy shapes are removed. **Step 3:** The fiber in the middle of the bundle is denoted as f_M and identified as the fiber with the lowest mean distance D_{ME} from all other fibers. The fiber f_M is shown with a larger width for illustrative purposes and is highlighted with a pink arrow. **Step 4:** Fibers are aligned to fiber f_M. Then, the centroid is calculated as the arithmetic mean of the corresponding fiber points.

Step 1: Filtering by fiber length. This step helps produce a longer centroid, covering the atlas bundle along its entire length. For this purpose, fibers with a length below the k-percentile are discarded. The algorithm is set with an initial length threshold equal to the 0-percentile (no fibers are discarded). If the resulting centroid needs to be recalculated, the length threshold is increased by 10 percentiles. The resulting atlas bundle from this step is denoted as B_l.

Step 2: Filtering by fiber shape. This step aims to discard fibers with noisy shape from the B_l bundle, which may alter the centroid's final shape. For example, fibers with an abnormal change in curvature along their trajectory. First, the fibers' mean D_{SHAPE} distance to every other fiber in the B_l bundle are calculated (see Fig. S6-A). Then, fibers with a mean D_{SHAPE} distance greater than the k-percentile are discarded.

In the following, we describe the procedure employed to determine an appropriate k-percentile threshold. First, we generate subsets of fibers with a mean distance D_{SHAPE} below the k-percentile, where k ranges from 10 to 100 with a step size of 10 (see Fig. S6-B1). Next, the fibers of each subset are aligned with the fiber with the lowest mean distance D_{SHAPE}, using the alignment method described in section S1.1 (see Fig. S6-B2). An average fiber is computed for each subset of aligned fibers as the arithmetic mean of the corresponding fiber points. The average fiber describes the overall shape of the subset and it is denoted AF_k (see Fig. S6-B3). Finally, for each subset of fibers, the sum of the square D_{SHAPE} distances to their corresponding average fiber is calculated. We used the Elbow method to determine an appropriate k-percentile threshold, which is the point where the sum of squared D_{SHAPE} distances begins to increase rapidly due to the inclusion of noisy shaped fibers.

Fig. S6-C displays the sum of the squared D_{SHAPE} distances for different subsets of fibers. In this example, the 70-percentile is used as the threshold, removing 30% of the fibers with the highest mean distance D_{SHAPE}. The resulting bundle of this step is denoted as B_{ls}.

Step 3: Identification of a fiber in the middle of the bundle (fiber f_M). This step is performed to ensure the computation of a centroid in the middle of the bundle B_{ls}. First, we calculate the fibers' mean distance D_{ME} to every other fiber in the bundle B_{ls}. Then, the fiber positioned in the middle of the bundle B_{ls} is identified as the one with the lowest mean distance D_{ME} and denoted as the fiber f_M. In Fig. S5-*Step 3* we show the fibers color-coded by their mean distance D_{ME}, from a low distance (black) to a high distance (red). It can be seen that most fibers with a high distance are located in the exterior part of the bundle, whereas fibers with a low distance are towards the core of the bundle.

Step 4: Alignment to fiber f_M and centroid computation. An alignment of each fiber from the B_{ls} bundle is performed on the fiber f_M, which helps to avoid errors due to the different positions and orientations of the fibers (see Fig. S5-*Step 4*). Finally, the atlas bundle centroid is computed from the aligned set of fibers as the arithmetic mean of the corresponding fiber points.

We used a length criterion to terminate the algorithm. If the centroid length is less than the average fiber length of the input atlas bundle, then the centroid is recalculated with a lower threshold for the step of filtering by fiber length (see Step 1). Otherwise, the algorithm is terminated.

S1.4 Alignment with scaling and atlas bundle centroids distance measure

In this section, we propose a dedicated atlas bundle centroids distance measure, called D_C. The D_C distance is based on the alignment and D_{SHAPE} distance (refer to sections S1.1 and S1.2). First, given two atlas bundle centroids A and B, we perform an alignment to focus solely on shape differences, independently from their positions. In addition, the alignment removes differences in centroid length, which is achieved by the following procedure. First, both centroids are aligned by their central point, resulting in centered centroids A_c and B_c. Then, the *Step-2* of the alignment is slightly modified to apply a rotation and an isotropic scaling factor to produce centroid B_{crs} (**c**entered, **r**otated and **s**caled). To help avoid confusion, this modified alignment will be referred to as the *alignment with the scaling of centroid B to centroid A*.

In the same way as the rotation, the isotropic scaling factor is also computed from the *Streamline-based registration* (Garyfallidis et al., 2015) method to enlarge or shrink the centroid B_c in order to maximally

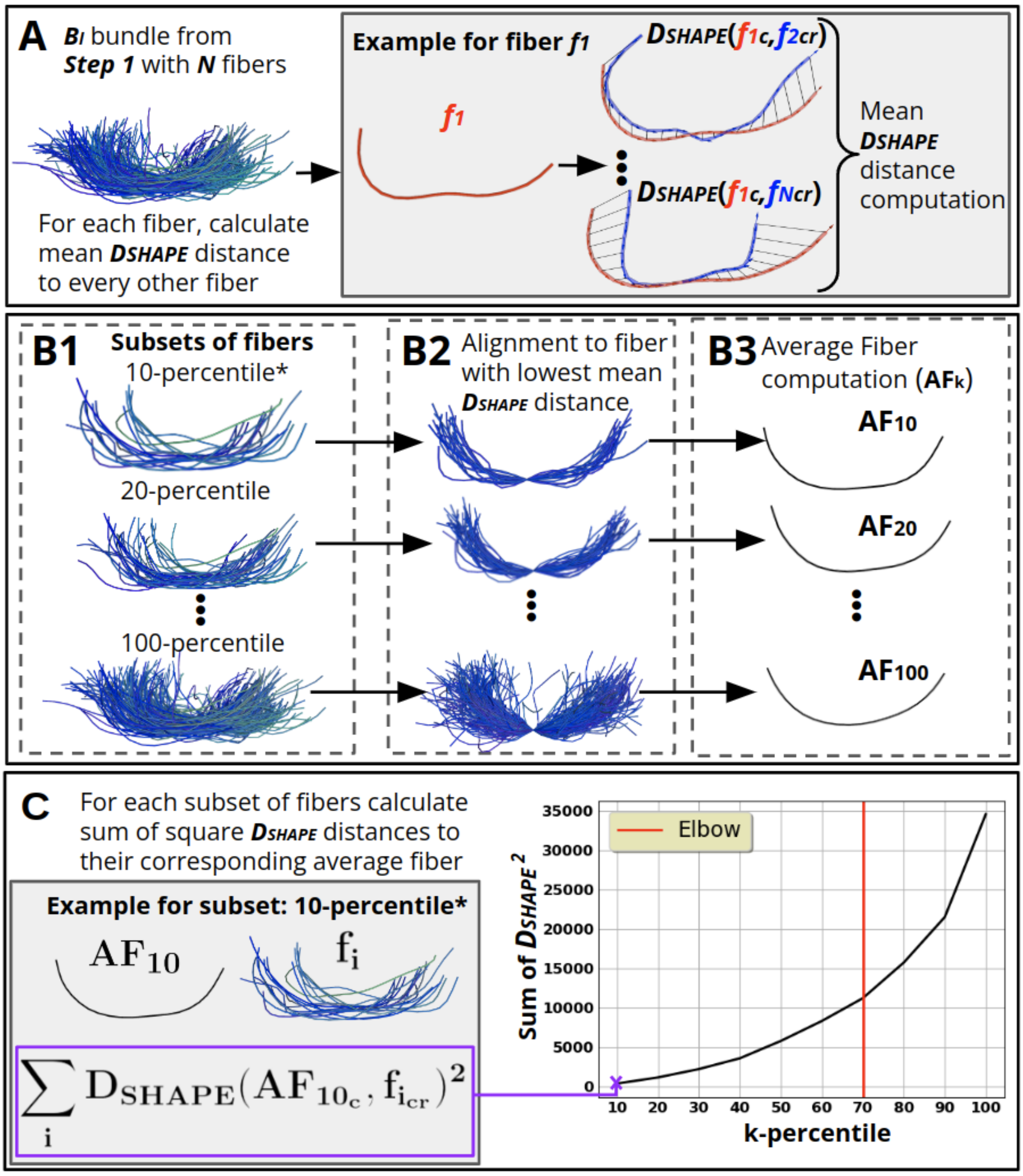

Figure S6. Fiber shape filtering. (A) The fibers' mean D_{SHAPE} distance to every other fiber in the B_l bundle are calculated. The D_{SHAPE} distance is calculated over a pair of aligned fibers. (B1) Subsets of fibers with a mean D_{SHAPE} distance less than a set of k-percentiles are generated. (B2) The fibers of each subset are aligned to the fiber with the lowest mean D_{SHAPE} distance. (B3) An average fiber is computed for each group of aligned fibers as the arithmetic mean of the corresponding fiber points. (C) An appropriate k-percentile threshold is defined using the Elbow method, which generates a subset of fibers with similar shape.

overlap its geometry to A_c. Therefore, to take into account the length of both centroids, two scenarios of alignment with scaling must be considered: (i) centroids A_c and B_{crs} are generated (see Fig. S7-B1); (ii) centroid A_{crs} and B_c are generated (see Fig. S7-B2). Based on the above, we define the D_C distance between two atlas bundle centroids A and B in Eq. S4:

$$D_C(A, B) = (D_{SHAPE}(A_c, B_{crs}) + D_{SHAPE}(B_c, A_{crs}))/2 \tag{S4}$$

The D_C distance is symmetric and illustrated in Fig. S7-C.

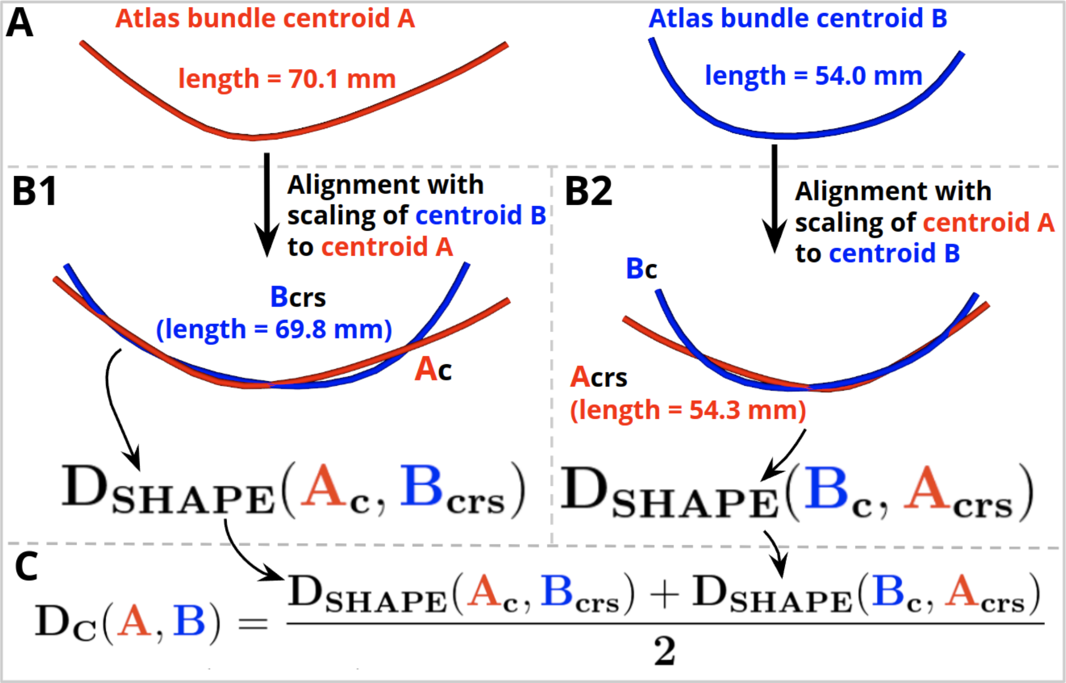

Figure S7. (A) Two atlas bundle centroids A and B and their respective length. (B1) Centroid B is centered, rotated and scaled. The isotropic scaling modifies centroid B length to be approximately equal to the length of centroid A (69.8 mm and 70.1 mm, respectively). (B2) The isotropic scaling modifies the length of centroid A to be approximately equal to the length of centroid B (54.3 mm and 54.0 mm, respectively). (C) The D_C distance is the average D_{SHAPE} distance considering both scenarios of alignment, (B1) and (B2), between atlas bundle centroids A and B.

S1.5 Hierarchical clustering over atlas bundle centroids

In this section, atlas bundle centroids are grouped by shape using hierarchical clustering. First, the D_C distance between every pair of atlas bundle centroids is computed, resulting in a distance matrix of the atlas bundle centroids. Then, a centroid affinity graph is calculated from the distance matrix. The affinity is calculated as $a_{ij} = e^{-D_C(i,j)^2/\sigma^2}$, where $D_C(i,j)$ is the D_C distance between atlas bundle centroids i and j. The parameter σ determines the similarity scale and is set to 60 mm, a value that has been successfully used to cluster short fibers (Guevara et al., 2017; Román et al., 2022). Next, the centroid affinity graph is used to perform an average-link hierarchical clustering. The clustering produces a hierarchical tree (dendrogram) with leaves representing atlas bundle centroids and nodes representing clusters. An adaptive partition of the hierarchical tree is used to calculate the clusters, using a maximum distance (D_{Clmax}) between centroids as a criterion to cut the dendrogram. The partition process starts from the top node and goes down to the leaves, calculating a maximum pairwise distance between descendant centroids for each node. A final cluster of atlas bundle centroids is generated when this distance is less than D_{Clmax}. We used values for D_{Clmax} that generate different numbers of clusters (N_c) (see Tab. S1).

D_{Clmax} (mm)	40	44	45	46	47	51	52	54
N_c	21	17	15	11	10	5	4	3

Table S1. Values of D_{Clmax} generating different number of clusters (N_c).

We computed the intra-cluster variation or Within Cluster Sum of Squares (WCSS) (Aggarwal and Reddy (2018)) to find the optimal number of clusters. Next, we describe the WCSS calculation for the data in Table S1. We use the notation Cl_{i,N_c} to refer to a cluster i of atlas bundle centroids belonging to the partition of N_c clusters. Also, the notation C_j refers to the atlas bundle centroid j. For each Cl_{i,N_c}, we identify the $C_j \in Cl_{i,N_c}$ with the lowest mean D_C distance to every other centroid in the cluster, which is denoted as $\hat{C}_{i,N_c}$. Then, we performed an alignment with scaling of each C_j to their corresponding $\hat{C}_{i,N_c}$, generating a cluster of aligned centroids. The cluster of aligned centroids i from the N_c partition is denoted as CCl_{i,N_c}. Then, we computed an average centroid from the CCl_{i,N_c} as the arithmetic mean of the corresponding centroid points (referred to as $\overline{C}_{i,N_c}$). Every notation is illustrated in Fig. S8-A. Finally, the WCSS for different N_c is computed in Eq. S5:

$$WCSS(N_c) = \sum_{i=1}^{N_c} \sum_{C_j \in Cl_{i,N_c}} D_C(C_j, \overline{C}_{i,N_c})^2 \tag{S5}$$

The idea is to use a number of clusters where the main atlas bundle shapes are represented. Thus, at the Elbow point the WCSS starts to decrease slowly due to small clusters with low representativeness. The Elbow method shows that five clusters are optimal (see Fig. S8-B). However, we discarded one cluster with size one, as it was a single sample. Therefore, we used the remaining four clusters to select shape representative atlas bundles.

S1.6 Selection of shape representative atlas bundles

As a final stage of the atlas processing, we used the four clusters $Cl_{i,5}$ $i \in [1,..,4]$, to select atlas bundles representing the main shapes in the SWM atlas. The cluster $Cl_{i,5}$ is used to identify the corresponding atlas bundles (see Fig. S8-C). The atlas bundle clusters are denoted BCl_i, where i is a correlative number that

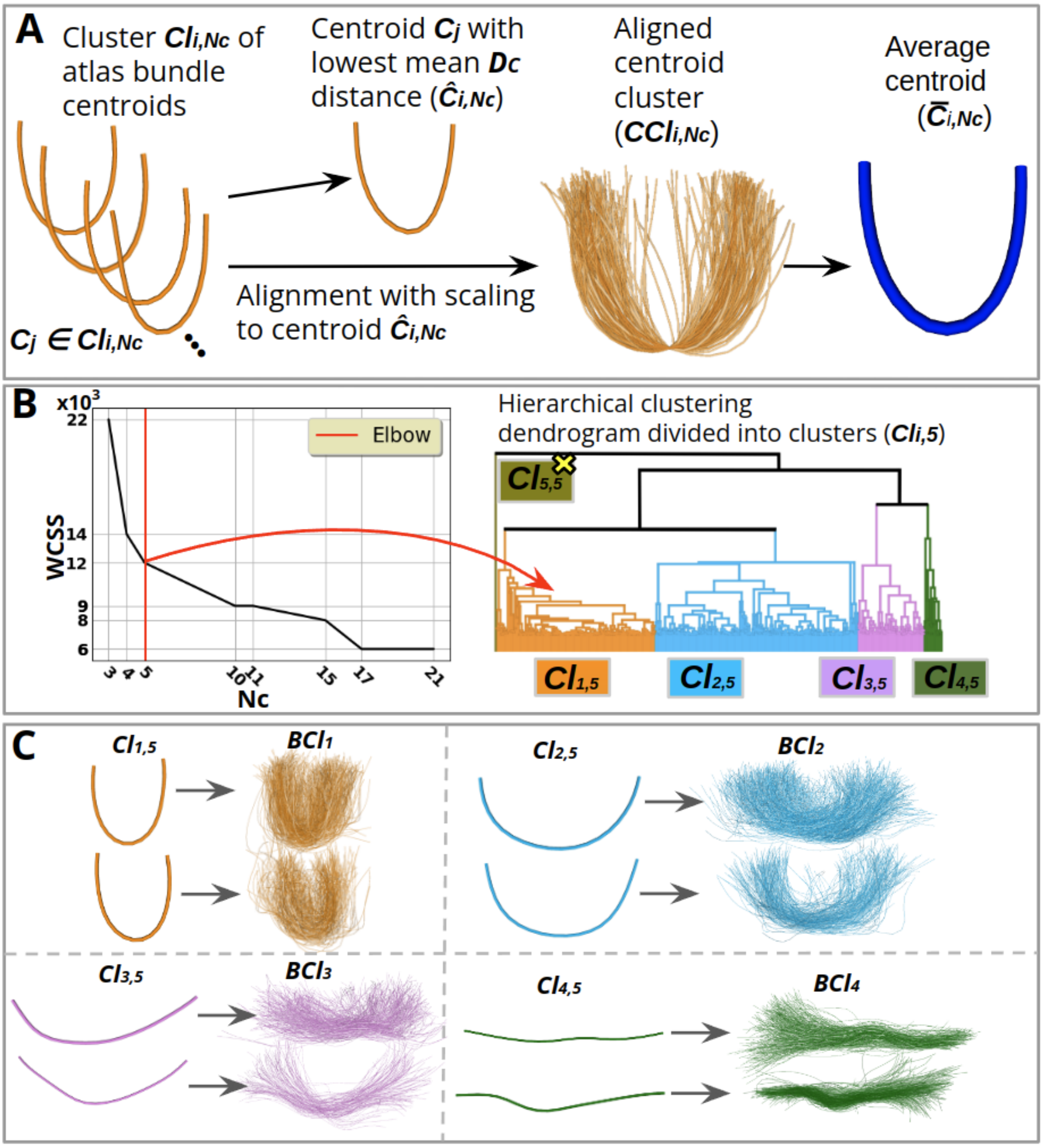

Figure S8. (A) For each cluster of atlas bundle centroids Cl_{i,N_c}, we identified the centroid with the lowest mean D_C distance to every other centroid in the cluster (denoted as $\hat{C}_{i,N_c}$). Then, each centroid $C_j \in Cl_{i,N_c}$ was aligned with scaling to centroid $\hat{C}_{i,N_c}$ to obtain an aligned centroid cluster (CCl_{i,N_c}). Finally, the arithmetic mean of the corresponding centroid points is computed from the CCl_{i,N_c} and denoted as $\overline{C}_{i,N_c}$. (B) We show the WCSS for different N_c. The Elbow method is used to find the optimal $N_c = 5$. (C) Using the centroids from cluster $Cl_{i,5}$, we identified the corresponding atlas bundles.

matches the number i of the cluster $Cl_{i,5}$. For each BCl_i, we used two bundles' features to generate 2D plots: the number of fibers and the mean length of the fibers.

Next, we searched around the average values of each axis to select an atlas bundle with a shape similar to the corresponding $\overline{C}_{i,5}$. Also, each selected atlas bundle is tagged with a label in the format *"Representative Bundle i"* (RB_i). In the following, we describe the location of each RB_i within the brain. RB_1: atlas bundle connecting the Precentral and Postcentral gyri from the right hemisphere. RB_2: atlas bundle connecting the Inferior-Parietal and Middle-Temporal gyri from the left hemisphere. RB_3: atlas bundle connecting the

Inferior-Parietal and Inferior-Temporal gyri from the right hemisphere. RB_4: atlas bundle connecting the Caudal-Anterior-Cingulate and Posterior-Cingulate gyri from the left hemisphere. These four atlas bundles are used to describe the main bundle shapes in the SWM atlas. In Fig. S9 we summarize and illustrate the processing performed to obtain shape representative atlas bundles.

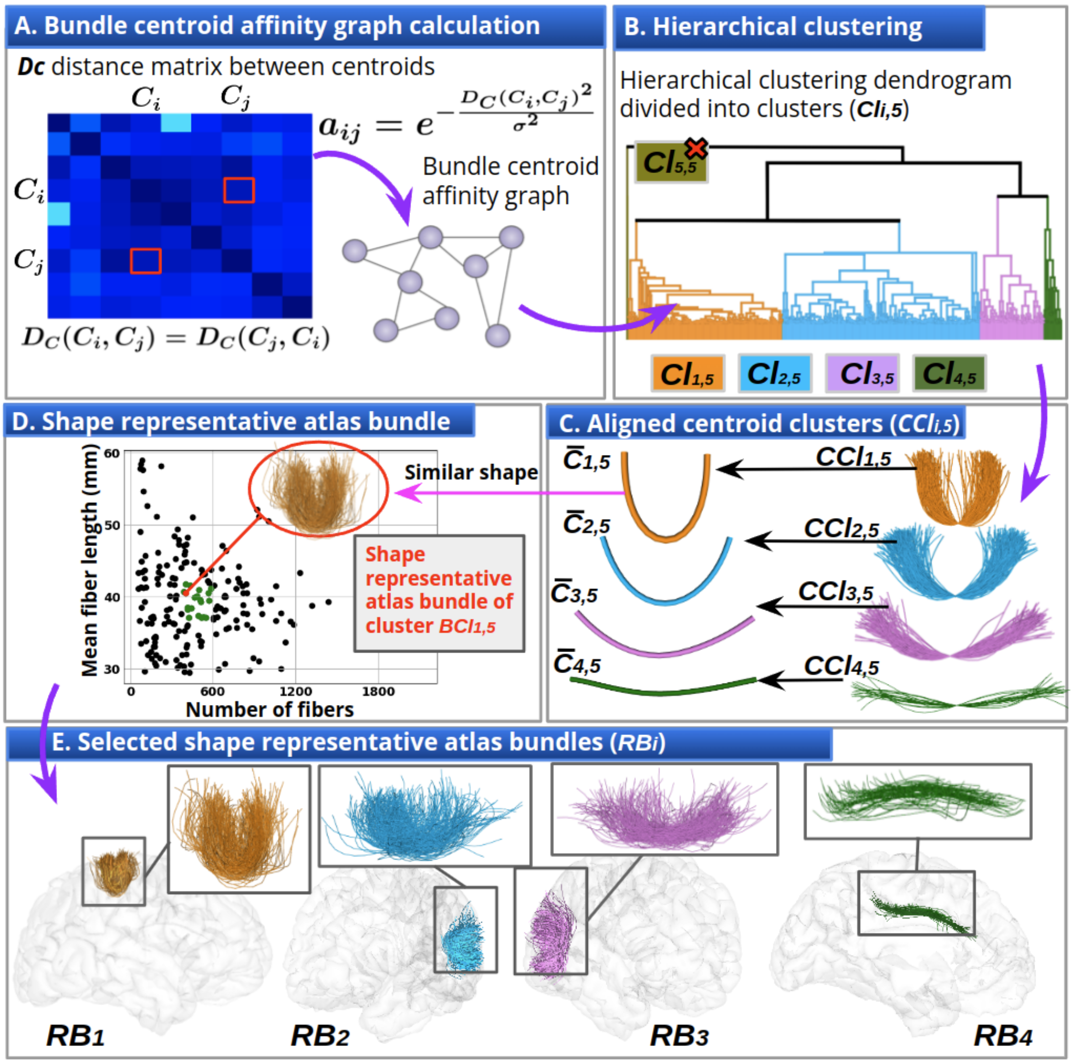

Figure S9. (A) The D_C distance between every pair of atlas bundle centroids is computed to generate a D_C distance matrix. Then, a centroid affinity graph is calculated from the distance matrix. (B) An average-link hierarchical clustering is performed on the centroid affinity graph to group atlas bundle centroids into clusters. We show the optimal clusters ($N_c = 5$), colored with different colors. (C) We show the aligned centroid clusters ($CCl_{i,5}$) of each cluster computed in (B). Also, their respective $\overline{C}_{i,5}$ is shown. (D) The 2D plot of atlas bundle cluster BCl_1. The horizontal axis is the atlas bundles' fiber count, whereas the vertical axis corresponds to the atlas bundles' mean fiber length. We searched around the average values of each axis (green dots) to select an atlas bundle with a shape similar to the corresponding $\overline{C}_{i,5}$. The shape representative atlas bundle from cluster BCl_1 is highlighted in a red circle, which is called RB_1. (E) The four RB_i over a cortical mesh to illustrate their position within the human brain.

S2 RESULTS FOR THE ATLAS BUNDLE CENTROIDS

S2.1 Atlas bundle centroid lengths

In Fig. S10-A/B we present a barplot displaying the mean fiber length of the 525 atlas bundles (blue bars), sorted from lowest to highest. Also, the centroid's length of each atlas bundle is overlayed as a red bar. In Fig. S10-A, atlas bundle centroids were calculated by only computing the arithmetic average of the corresponding fiber points, without any further processing. This is a traditional method in the field for the calculation of centroids (Yeatman et al., 2012; Colby et al., 2012). In Fig. S10-B, we show the length of the centroids calculated with our proposed method. It can be seen that centroids calculated using our scheme are longer and achieve a length approximately equal to the mean fiber length of the atlas bundles (a blue line indicates the top of the blue bars). In addition, Fig. S10-C shows a qualitative comparison for both methods. It can be seen that our proposed method generated centroids with better coverage of the atlas bundle, adequately describing the overall bundle shape. Whereas the traditional method generated shorter centroids with an irregular geometry.

The average difference between the centroid's length and the atlas bundle mean fiber length were $1.01 \pm 0.83mm$ and $10 \pm 2.8mm$ for the proposed and traditional method, respectively. Finally, the mean percentage of fibers removed for the filtering by fiber length and shape (see section S1.3, *Step 1* and *Step 2*) were $45\% \pm 15\%$ and $30\% \pm 6\%$, respectively.

S2.2 Projection of the centroids and atlas bundle density images

In this section we quantify how well the proposed centroids fit their corresponding atlas bundle shape. For each centroid, a 2D plane was generated using the endpoints and the central point. First, vectors v_1 and v_2 were computed, directed from the central point to the endpoints. Then, using v_1 and v_2, the perpendicular vector v_3 was calculated. Finally, the cross product between v_1 and v_3 generated the perpendicular vector v_4 (see Fig. S11).

A density image was computed for each atlas bundle, and voxels with a density value less than 10% of the maximum density value were removed (Horbruegger et al., 2019), allowing us to obtain the *core* of the bundle. For each atlas bundle, the density image and the centroid of the bundle were projected onto the v_1-v_4 plane. The distance from each projected voxel to the nearest point of the projected centroid was calculated, and these distances were averaged. This quantified the similarity between the centroid and the overall morphology of the atlas bundle. As expected, a smaller average distance was obtained for the proposed centroids, as they are located in denser regions. In contrast, the traditional centroid yielded larger distances due to an inadequate coverage of the bundle (see Fig. S12). The mean$\pm$standard deviation of the average distances for the proposed and traditional centroids were $3.86 \pm 0.92mm$ and $4.02 \pm 0.94mm$, respectively.

S3 BUNDLE DENSITY IMAGE CALCULATION

We calculated bundle density images composed of $1mm \times 1mm \times 1mm$ voxels. To map fiber points to voxels, fibers were upsampled with a maximum distance between points of $1mm$ to consider most of the voxels containing the fiber trajectory. Also, a maximum of one point per fiber in each voxel was considered for density image computation.

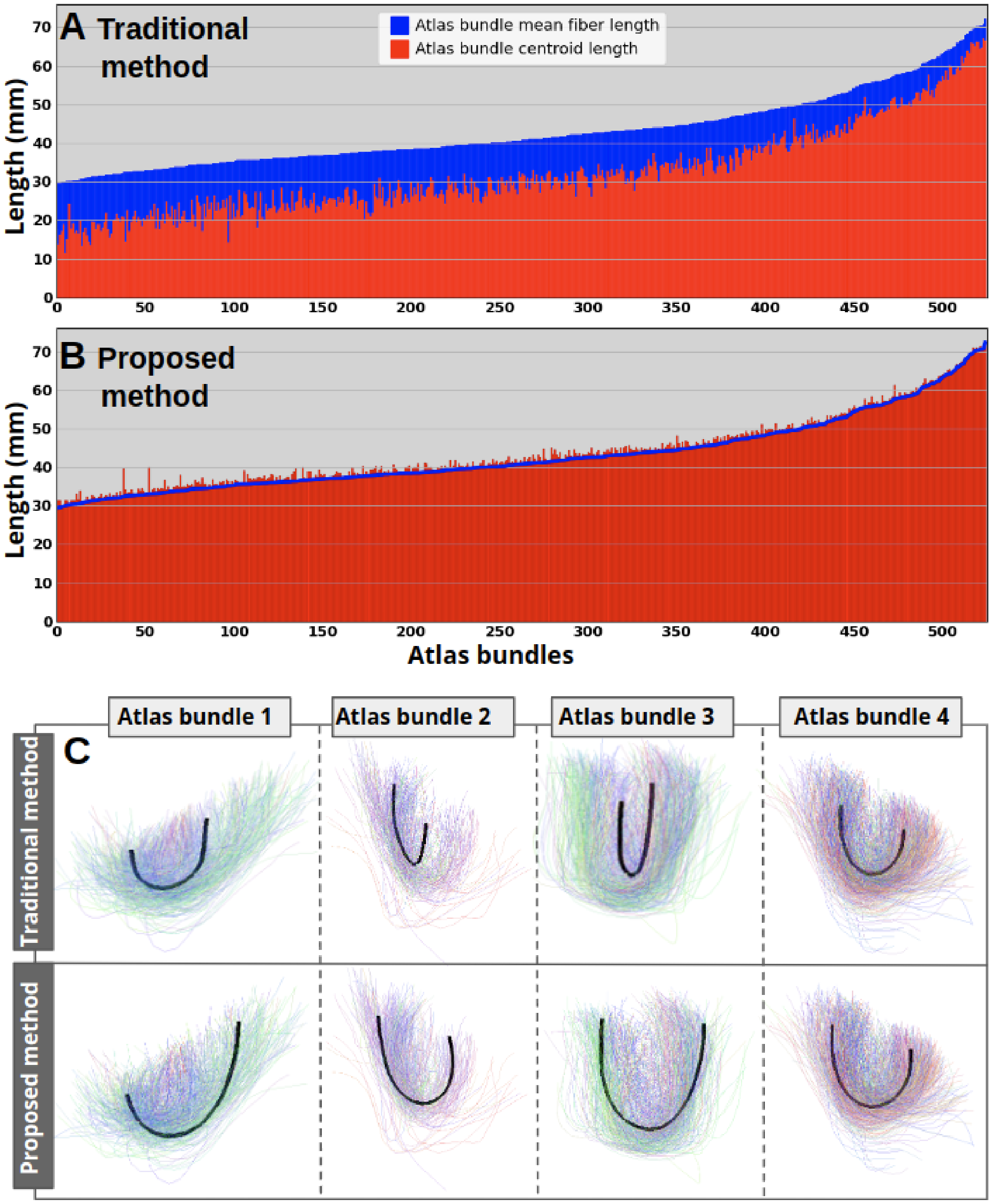

Figure S10. (A) Bar plot displaying the mean fiber length of each atlas bundle (blue) and the length of their corresponding centroids (red). In this case, centroids were calculated as the arithmetic average of the corresponding fiber points. (B) Bar plot displaying the mean fiber length of each atlas bundle (a blue line is drawn to highlight the top of the blue bars) and the length of their corresponding centroids (red). In this case, centroids were calculated using our proposed method. (C) Four atlas bundles and their corresponding centroids (black) from the traditional and proposed method.

S4 AUTOMATIC ELBOW POINT DETECTION

We used the Kneedle algorithm (Satopaa et al., 2011) with default parameters to automatically detect Elbow points, which is publicly available at `https://github.com/arvkevi/kneed`. The Kneedle

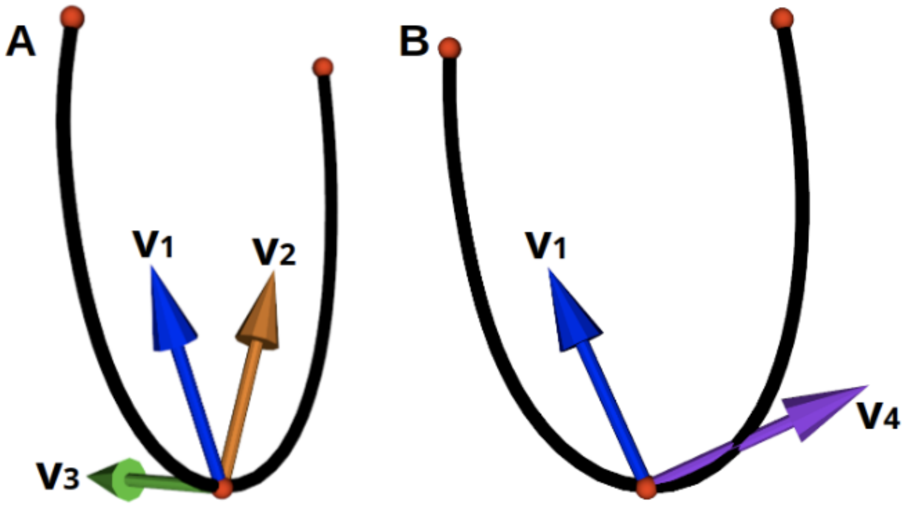

Figure S11. Vectors from an atlas bundle centroid. (A) Vectors v_1, v_2, v_3. (B) Vectors v_1 and v_4 defining a 2D plane.

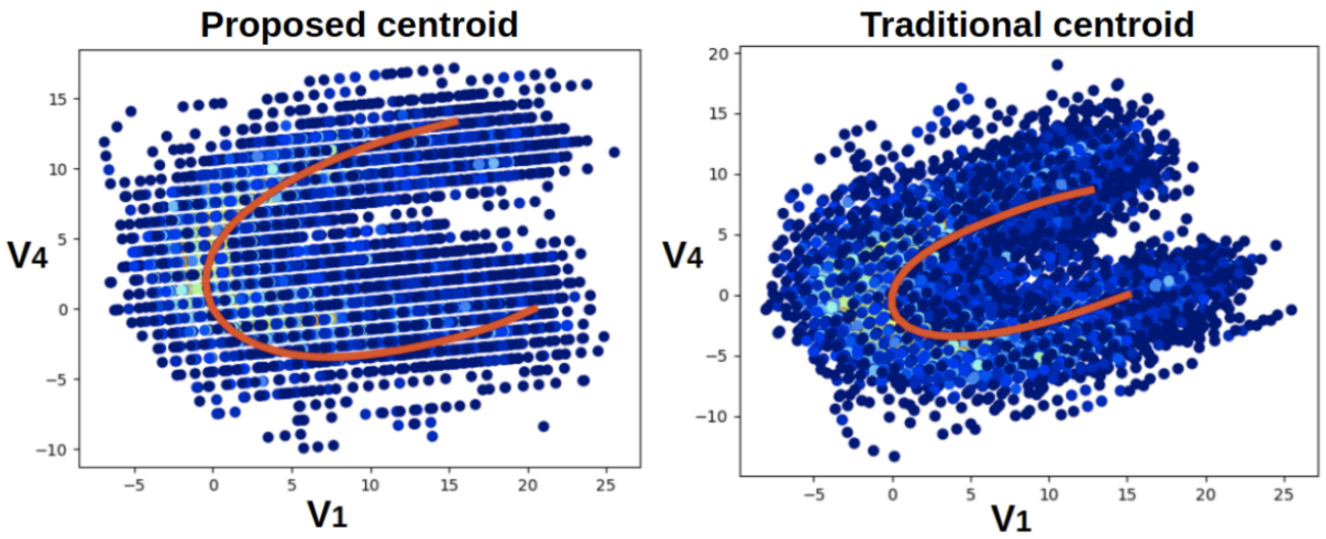

Figure S12. Projection onto the v_1-v_4 plane of the atlas bundle density image (blue) and centroid (red). The average distance of the projected voxels to the nearest point of the projected proposed centroid was $2.49mm$. In contrast, the average distance was $2.69mm$ for the traditional centroid. It can be seen that the proposed centroid covers the atlas bundle along its entire length.

algorithm determines the Elbow as the point of maximum curvature, where the curvature is a mathematical measure of how much a function differs from a straight line.

S5 TRMD CURVES FROM THE TRAINING SET (HCP DATABASE)

Fig. S13 shows TRMD curves for a single subject. Fig. S14 shows the averaged TRMD curves for the 28 subjects in the training set.

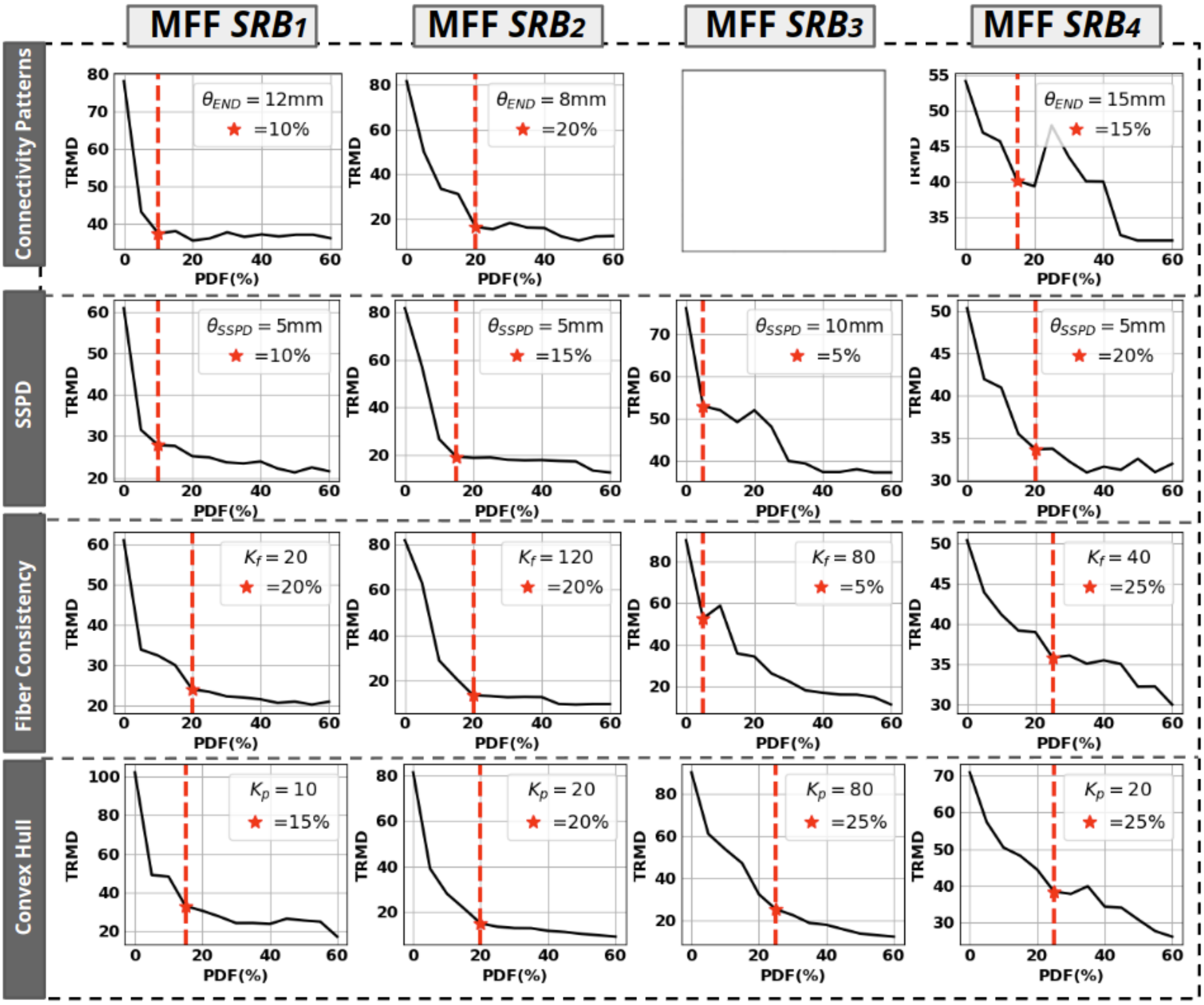

Figure S13. TRMD curves of subject 861456 from the training set. Notice that TRMD curves at the individual level could be noisier if the fiber bundle filter does not adequately filter spurious fibers. For example, see the fiber bundle filter based on Connectivity Patterns applied in the MFF SRB_4, where the TRMD increases at the middle of the curve. This is because the filter only considers endpoints information. Thus, after filtering, the bundle could still have noisy and isolated fibers. Also, to avoid selecting a TRMD curve without an overall convex shape, we only considered TRMD curves with an Elbow point between a PDF in the range of $0\% - 30\%$. TRMD values are in $\times 10^2$.

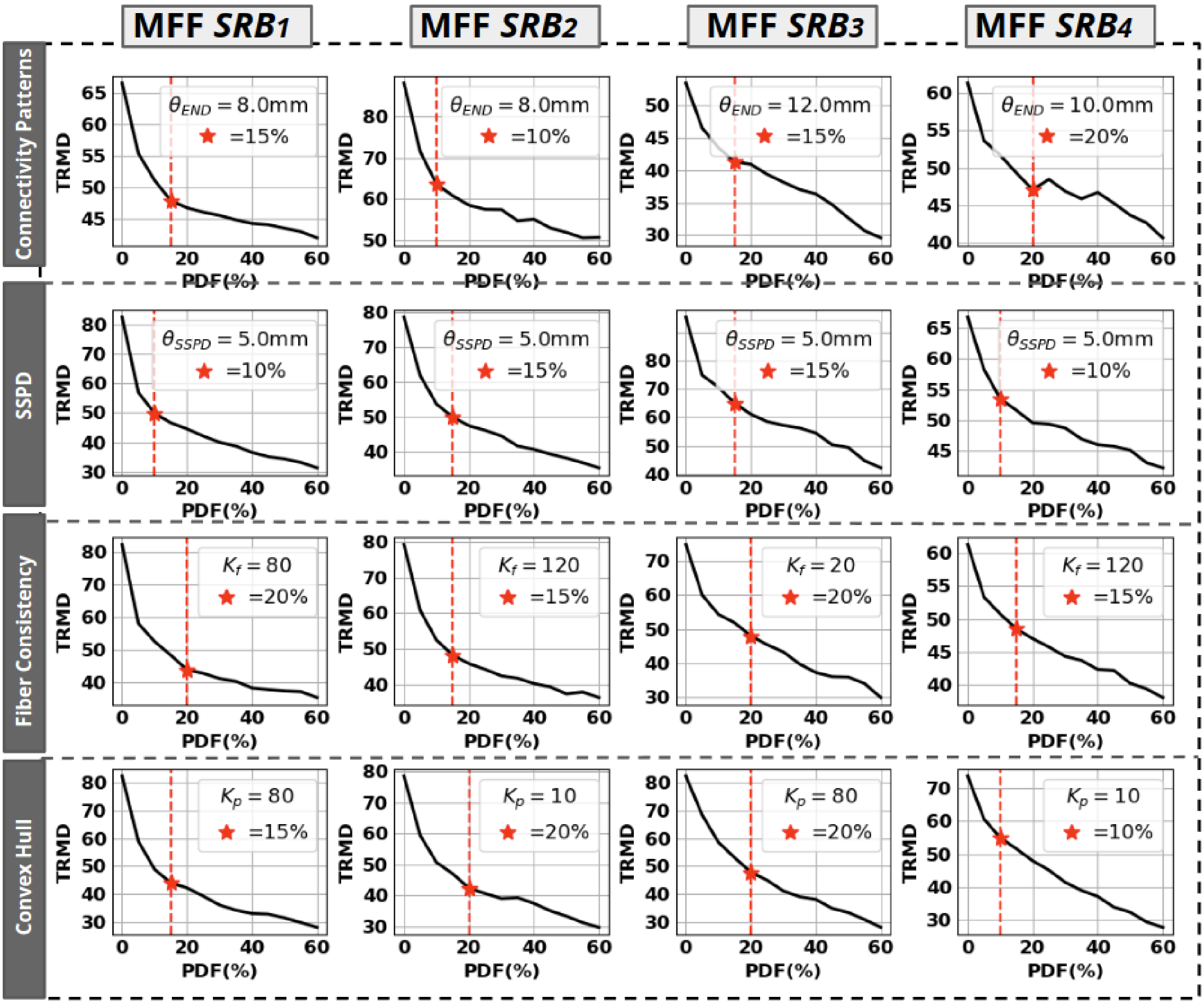

Figure S14. Average TRMD curves using the 28 subjects from the training set. The PDF(%) is determined from the horizontal axis of the Elbow point (red star). Also, the filters' second parameter (θ_{END}, θ_{SSPD}, K_f, K_p) corresponds to the statistical mode of the selected curves from the 28 subjects. The mean number of TRMD curves considered for each fiber bundle filter and MFF SRB_i was 26 ± 2 curves. A summary of the parameters is shown in Table 2 of the main manuscript. TRMD values are in $\times 10^2$.

S6 COMBINATIONS OF THE FIBER BUNDLE FILTERS IN THE TRAINING SET (HCP DATABASE)

We quantified the improvement in the test-retest reproducibility indices for every possible combination between fiber bundle filters. In Fig. S15, we show the median value of the test-retest reproducibility indices for the main fiber fascicles, processed with every possible filter combination. Each filter is denoted by a number from 1 to 4. Therefore, the vertical axis indicates whether two or more filters were combined.

For reproducibility measures based on binary masks, such as the Dice Volumetric Overlap and the AFD, the combination of filters showed minimal improvement in the median values. Notably, high scores were obtained by only applying the filter based on the Convex Hull, on par with several different combinations of filters.

The results for AD show that the fibers of the test-retest fascicles come into closer spatial proximity as the number of combined filters increases. This trend is illustrated in the third column of Fig. S15, where the combination of three or four filters obtained the lowest distances. However, compared to applying only the Convex Hull filter, the reduction was small ($0.5mm{\sim}1mm$ in the four MFF SRB_i)

Lower AMD values were obtained by only applying the filter based on the Convex Hull, and combining filters achieved a marginal improvement (reduction less than $0.1mm$). These findings suggest that the filter based on the Convex Hull removed spurious fibers ithout excessively reducing the bundles' diameter.

In addition, we computed the percentage of fibers discarded for every possible combination between filters. Then, we averaged this value for corresponding test-retest fascicles and denoted it as APDF (Average Percentage of Discarded Fibers). In Fig. S16, we show bar plots displaying the median values of the APDF for the 28 subjects, with standard deviation error bars. As expected, the median APDF increases as the number of combined filters increases, with the combination of four filters yielding the highest fiber discarding rate. The combination of four filters resulted in a median APDF of $\sim$30%, indicating an appropriate agreement in identifying noisy fibers between filters.

S7 BEST FIBER BUNDLE FILTER PERFORMANCE WITHOUT THE IDENTIFICATION OF THE MAIN FIBER FASCICLE IN THE TRAINING SET (HCP DATABASE)

This section presents test-retest reproducibility indices for each SRB_i, without the identification of the main fiber fascicle , and processed with the fiber bundle filter based on the Convex Hull. We use the label "No Processed" (NP) to refer to segmented fiber bundles with neither the identification of the main fiber fascicle nor the fiber bundle filter based on the Convex Hull processing. Also, the label CH refers to segmented fiber bundles processed only with the Convex Hull filter.

In Table S2 we show the mean score for each test-retest reproducibility index. Next, we describe the results of applying the filter based on the Convex Hull to NP bundles. The mean Dice Volumetric Overlap score for the NP bundles shows a relatively good agreement in the volume occupied. The CH bundles had significantly higher DSC scores than the NP bundles for the four SRB_i (p-value< 0.05 for each comparison). Also, the CH bundles had significantly higher AFD scores than the NP bundles for the four SRB_i (p-value< 0.05 for each comparison), resulting in bundles with a smoother and more regular shape.

The CH bundles had significantly lower AMD scores than NP bundles for the four SRB_i (p-value< 0.05 for each comparison), which translates to filtered bundles being more compact and with fewer isolated

fibers. The CH bundles had significantly lower AD scores than NP bundles (p-value< 0.05 for each comparison), resulting in test-retest bundles with fibers spatially closer to each other. We show a fiber bundle with and without processing in Fig. S17-A. It can be seen that filtered bundles have fewer spurious fibers (CH and MFF+CH bundles), improving the similarity between test-retest acquisitions. We observed that only applying the identification of the main fiber fascicle did not overall improve the reproducibility indices. However, the identification of the main fiber fascicle plus filtering resulted in well-defined fiber bundles (see Fig. S17-B).

	DSC		AFD	
	NP	CH	NP	CH
SRB_1	0.76±0.06	**0.78±0.07**	2.22±0.06	**2.27±0.07**
SRB_2	0.75±0.04	**0.80±0.05**	2.07±0.07	**2.17±0.07**
SRB_3	0.65±0.11	**0.67±0.11**	1.90±0.19	**1.98±0.20**
SRB_4	0.76±0.05	**0.79±0.06**	2.15±0.07	**2.22±0.07**
	**AMD**		**AD**	
	NP	CH	NP	CH
SRB_1	3.61±0.48	**3.52±0.54**	14.33±1.65	**13.34±1.86**
SRB_2	3.06±0.44	**2.90±0.47**	13.37±1.33	**11.91±1.65**
SRB_3	4.26±0.85	**4.11±0.91**	16.26±0.98	**14.47±1.25**
SRB_4	2.53±0.34	**2.44±0.37**	14.69±1.45	**13.79±1.54**

Table S2. Mean scores for each test-retest reproducibility index and SRB_i (mean ± sd). The bold values indicate an improvement of the score after applying the filter based on the Convex Hull over the unprocessed bundles.

S8 ABBREVIATION OF EACH PROCESSING APPLIED TO THE SHORT FIBER BUNDLES

In Tab. S3, we present the abbreviations for the different processings applied to the short fiber bundles.

MFF	MFF+CP	MFF+SSPD	MFF+FC	MFF+CH
Segmented fiber bundles processed with the identification of the main fiber fascicle (MFF)	MFF following a fiber bundle filtering based on Connectivity Patterns (CP)	MFF following a fiber bundle filtering based on SSPD	MFF following a fiber bundle filtering based on Fiber Consistency (FC)	MFF following a fiber bundle filtering based on the Convex Hull (CH)

Table S3. Abbreviations used to describe segmented fiber bundles with different processing.

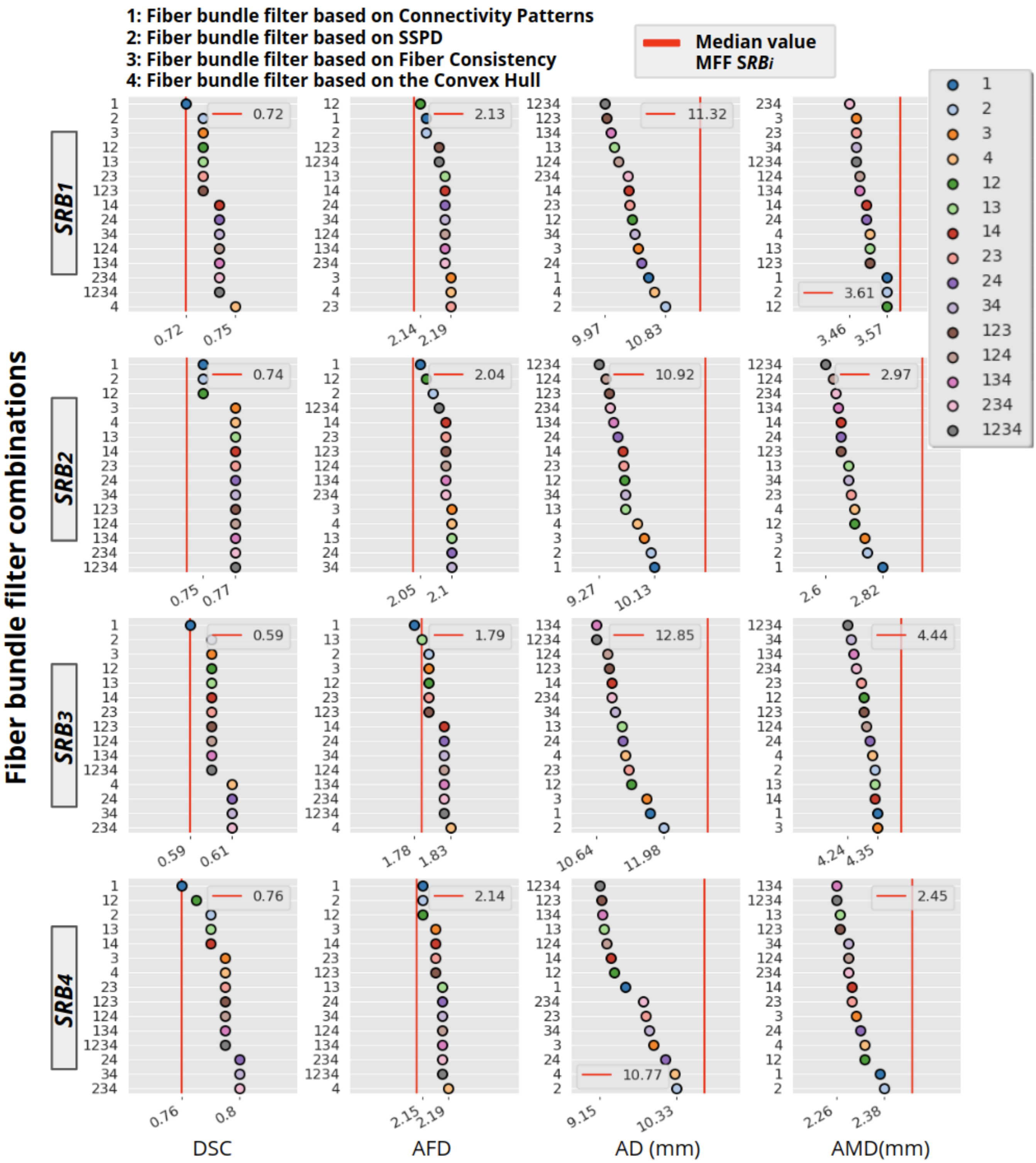

Figure S15. The red line represents the median value of the test-retest reproducibility indices for the MFF SRB_i, and the blue dots denote these indices after applying various combinations of fiber bundle filters. Each column corresponds to a specific test-retest reproducibility index, and the rows indicate the filtered SRB_i. The horizontal axis displays the range between the minimum and maximum median values observed among the filtered bundles.

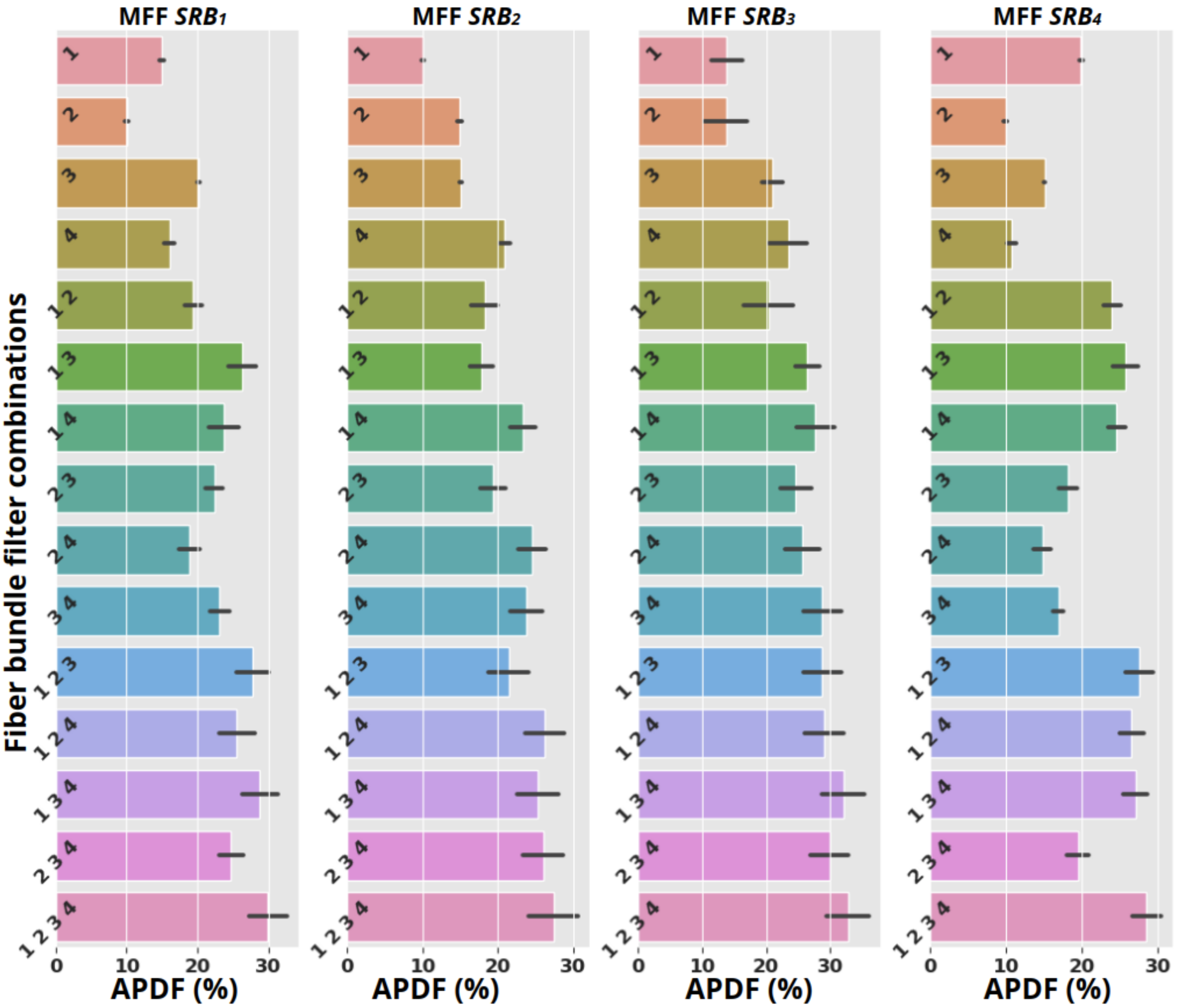

Figure S16. Average Percentage of Discarded Fibers (APDF) for every possible fiber bundle filter combination applied to the MFF SRB_i. Each fiber bundle filter is coded with a number from 1 to 4. Therefore, the vertical axis indicates which filters were combined. 1: Connectivity Patterns, 2: SSPD, 3: Fiber Consistency, 4: Convex Hull. We show bar plots of the median APDF for the 28 subjects, with standard deviation bars.

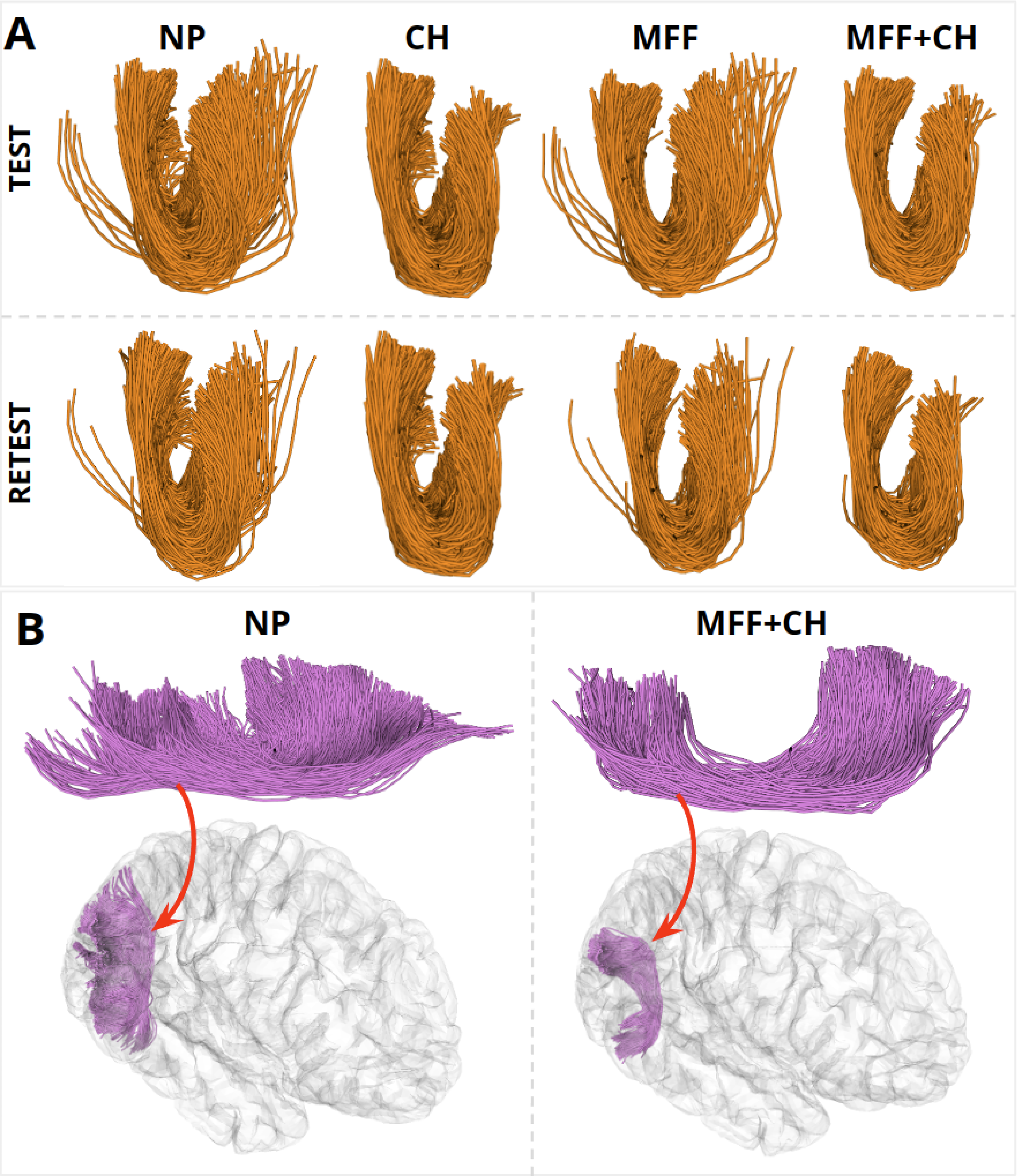

Figure S17. (A) Fiber bundle SRB_1 of subject 115320, with and without processing. (B) Fiber bundle SRB_3 of subject 192439, with and without processing. It can be seen that the identification of the main fiber fascicle and the fiber bundle filter based on the Convex Hull removed more spurious fibers and generated a well-defined structure of the bundle.

S9 ADDITIONAL RESULTS IN THE VALIDATION SET (HCP DATABASE)

S9.1 Random filtering of fibers

We applied random filtering of fibers to show that the test-retest reproducibility indices were better due to the discarding spurious fibers and not due to the bundles' fiber count. For each bundle, we set a percentage of discarded fibers equal to the PDF value of the filter based on the Convex Hull. Subsequently, fibers were randomly removed from the bundle until the discarded percentage was reached. In Table S4, we show the mean score for the total set of 7392 bundles. It can be seen that the test-retest reproducibility indices had no improvement and even worsened in most cases.

Test-retest reproducibility indice	NP	Random filtering	MFF	MFF+ Random filtering
Dice Volumetric Overlap	0.70±0.11	0.69±0.12	0.66±0.15	0.64±0.16
Average Fractal Dimension (AFD)	2.01±0.21	1.98±0.23	1.92±0.29	1.89±0.30
Average Minimum Distance (AMD)	3.68±0.91	3.79±0.96	3.88±1.24	4.00±1.28
Average Distance (AD)	15.70±2.91	15.69±2.91	12.53±2.12	12.54±2.12

Table S4. Mean scores of the test-retest reproducibility indices when performing a random filtering. The Dice Volumetric Overlap shows the mean DSC. The AMD and AD are in mm.

S9.2 Improvement in test-retest reproducibility indices

In Table S5 we show the number of short fiber bundles with a significant improvement in test-retest reproducibility indices.

Test-retest reproducibility indice	Number of bundles (between NP and CH)	Number of bundles (between MFF and MFF+CH)
Dice Volumetric Overlap	378/462	308/462
Average Fractal Dimension (AFD)	459/462	430/462
Average Minimum Distance(AMD)	342/462	347/462
Average Distance (AD)	462/462	460/462

Table S5. Number of fiber bundles with a significant improvement in the test-retest reproducibility indices when applying the fiber bundle filter based on the Convex Hull (p-value< 0.05).

S10 FIBER CONFIGURATION ANALYSIS

To analyze the configuration of rejected fibers, we applied the QuickBundles clustering (Garyfallidis et al. (2012)) to segmented bundles, filtered bundles and rejected fibers of the training set from the HCP database (28 subjects). We used the four representative bundles of each subject. For the clustering, we used a threshold of 8 mm. In Fig. S18 we show histogram plots of the mean cluster size (number of fibers) and frequency for all the resulting clusters. It can be seen in Fig. S18-A that the clustering of segmented bundles generates clusters with an average size in the range of [1-96] fibers with a frequency decreasing from 100 for the range [1-6] fibers, to around 20 for the range [91-96] clusters. Furthermore, filtered bundles present fewer small clusters, as shown in Fig. S18-B, with less than 50 clusters with an average size in the range [1-6]. On the other side, rejected fibers were clustered into a large number of small clusters, with a frequency ranging from 190 to 300 clusters for the size range [1-6] fibers (Fig. S18-C). The results show that in general, the filtering removes spurious fibers. However, it may exist some atlas bundles with one or more subpopulations of fibers.

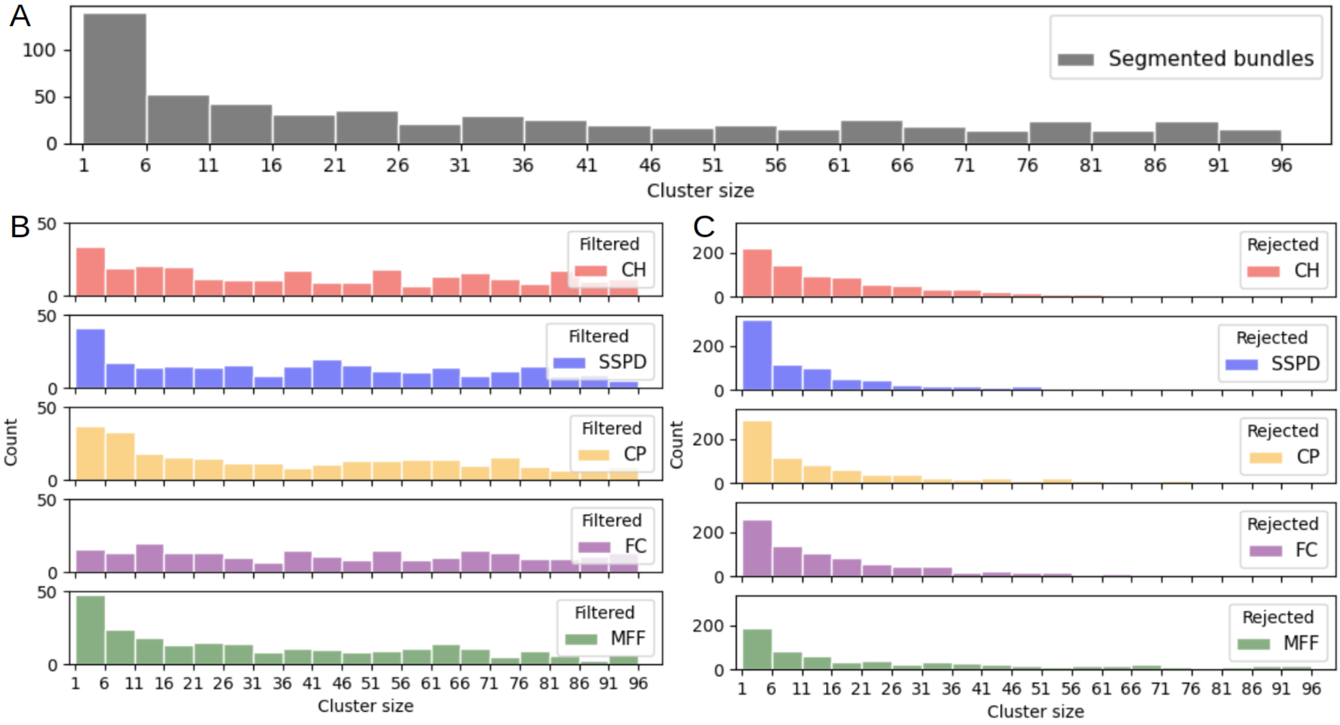

Figure S18. Histograms displaying the average cluster size of the representative segmented bundles after applying the QuickBundles algorithm with a threshold of 8 mm. (A) Results for the segmented representative bundles. (B) Results for the filtered bundles. (C) Results for the rejected fibers. CH: Filtering based on the Convex Hull. SSPD: Filtering based on the SSPD distance. CP: Filtering based on Connectivity Patterns. FC: Filtering based on Fiber Consistency. MFF: selection of the main fiber fascicle.

S11 REJECTED AND ACCEPTED FIBERS OF THE FIBER BUNDLE FILTERS

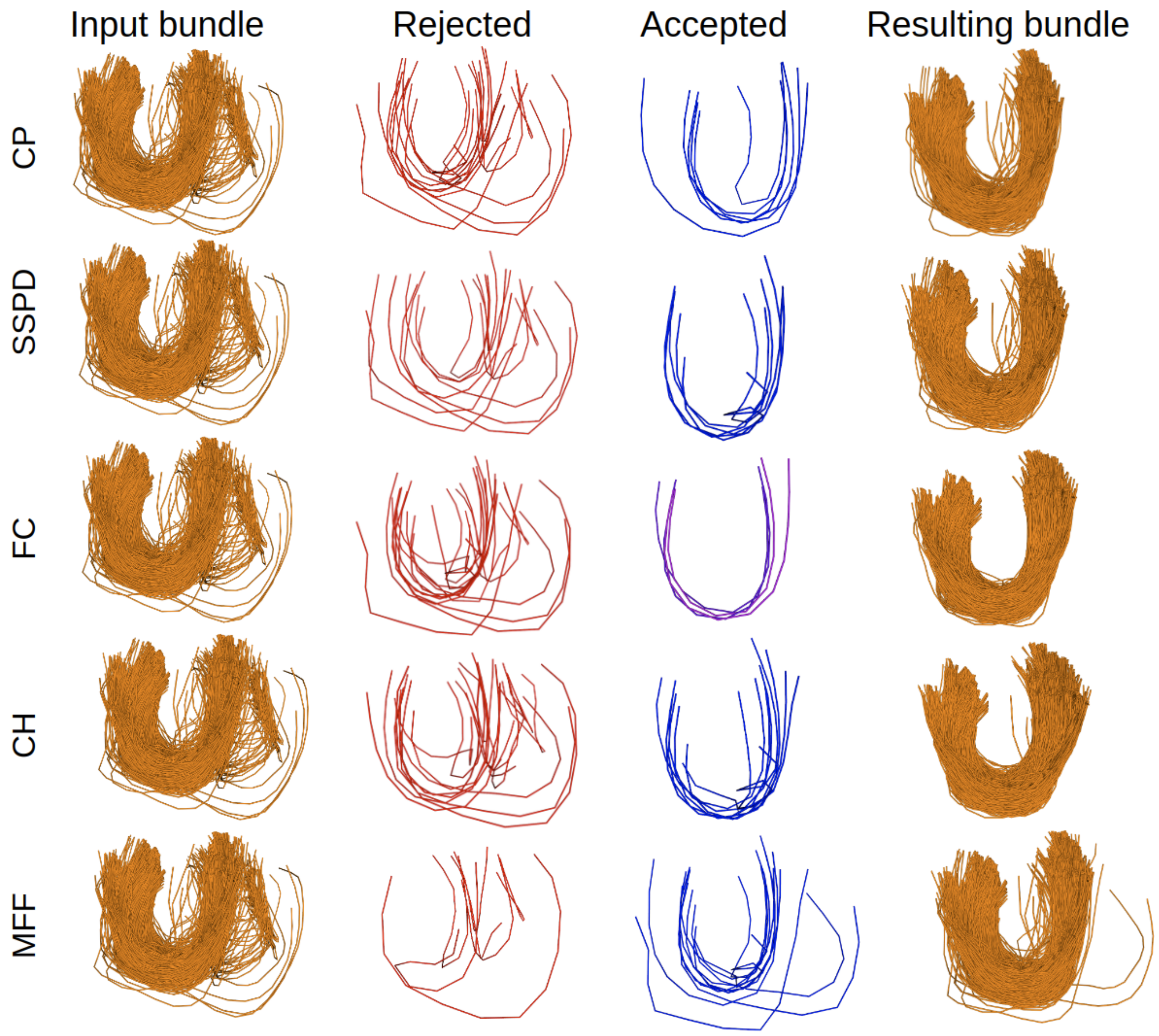

Figure S19. Example of rejected and accepted fibers for the four filters, illustrated by the centroids of resulting fiber clusters. Representative U-shaped short fiber bundle connecting the precentral and postcentral regions of subject 169343 of the HCP database.

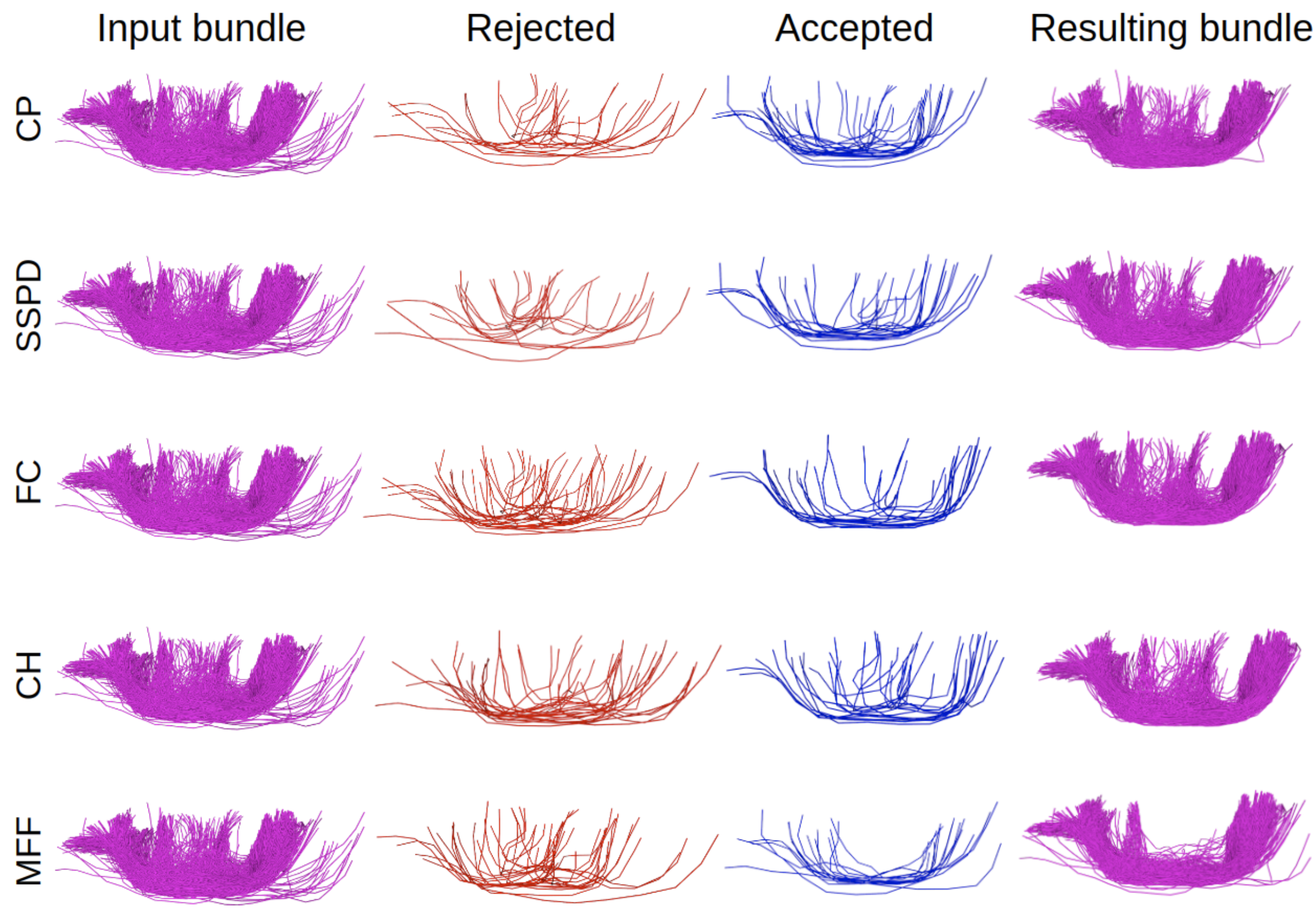

Figure S20. Example of rejected and accepted fibers for the four filters, illustrated by the centroids of resulting fiber clusters. Representative open U-shaped short fiber bundle connecting the inferior parietal and inferior temporal regions of subject 187547 of the HCP database.

S12 SEGMENTED BUNDLES FROM THE HCP DATABASE

Figures S21-S26 show segmented and filtered short fiber bundles from the validation set of the HCP database.

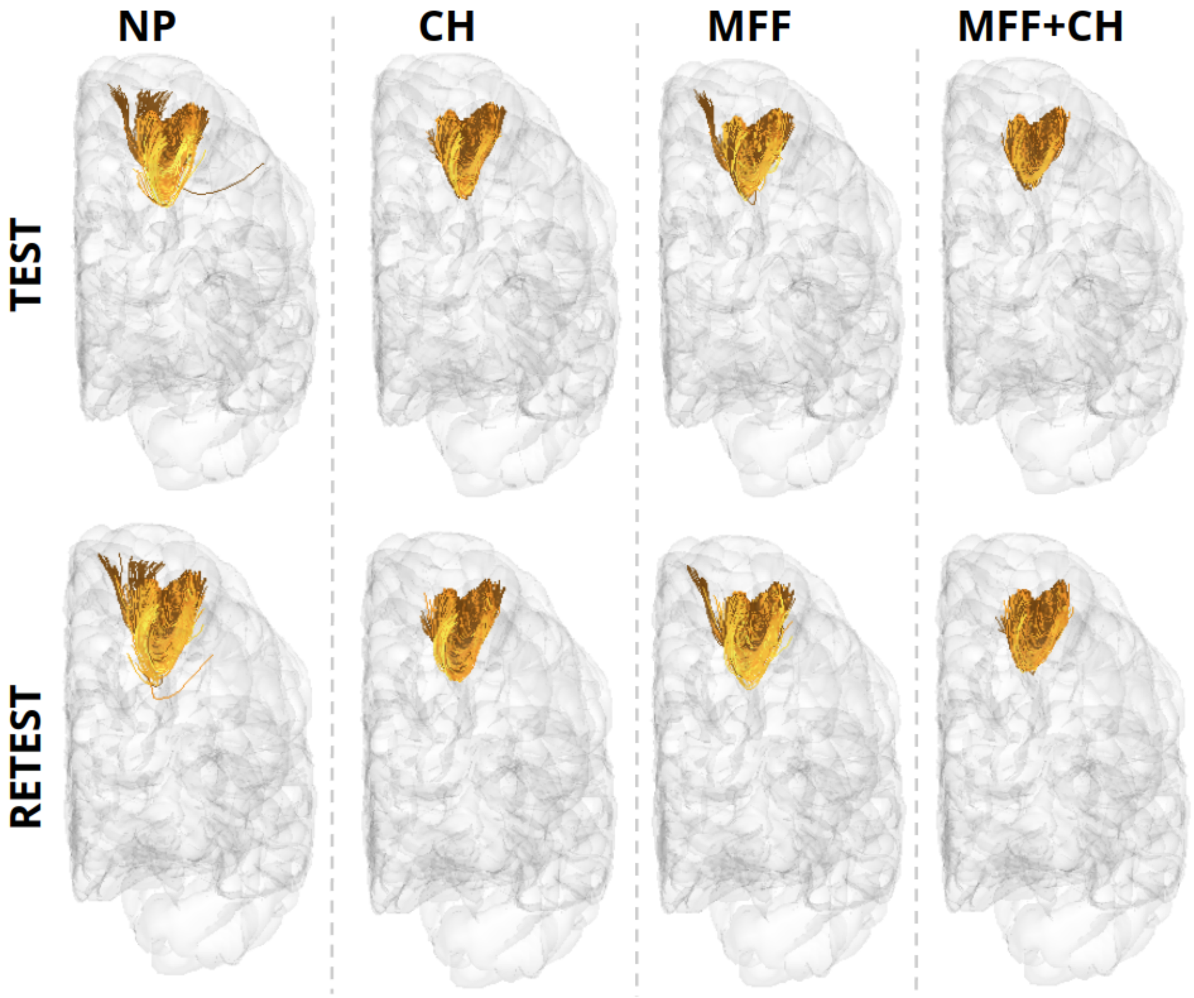

Figure S21. Subject 122317 from the validation set. We show a segmented fiber bundle connecting the Caudal Middle Frontal and Superior Frontal gyri from the brain's left hemisphere. NP: No processed, CH: fiber bundle filtered with the filter based on the Convex Hull. MFF: the identification of the main fiber fascicle of the bundle. MFF+CH: the identification of the main fiber fascicle was applied followed by the fiber bundle filter based on the Convex Hull. Coronal view.

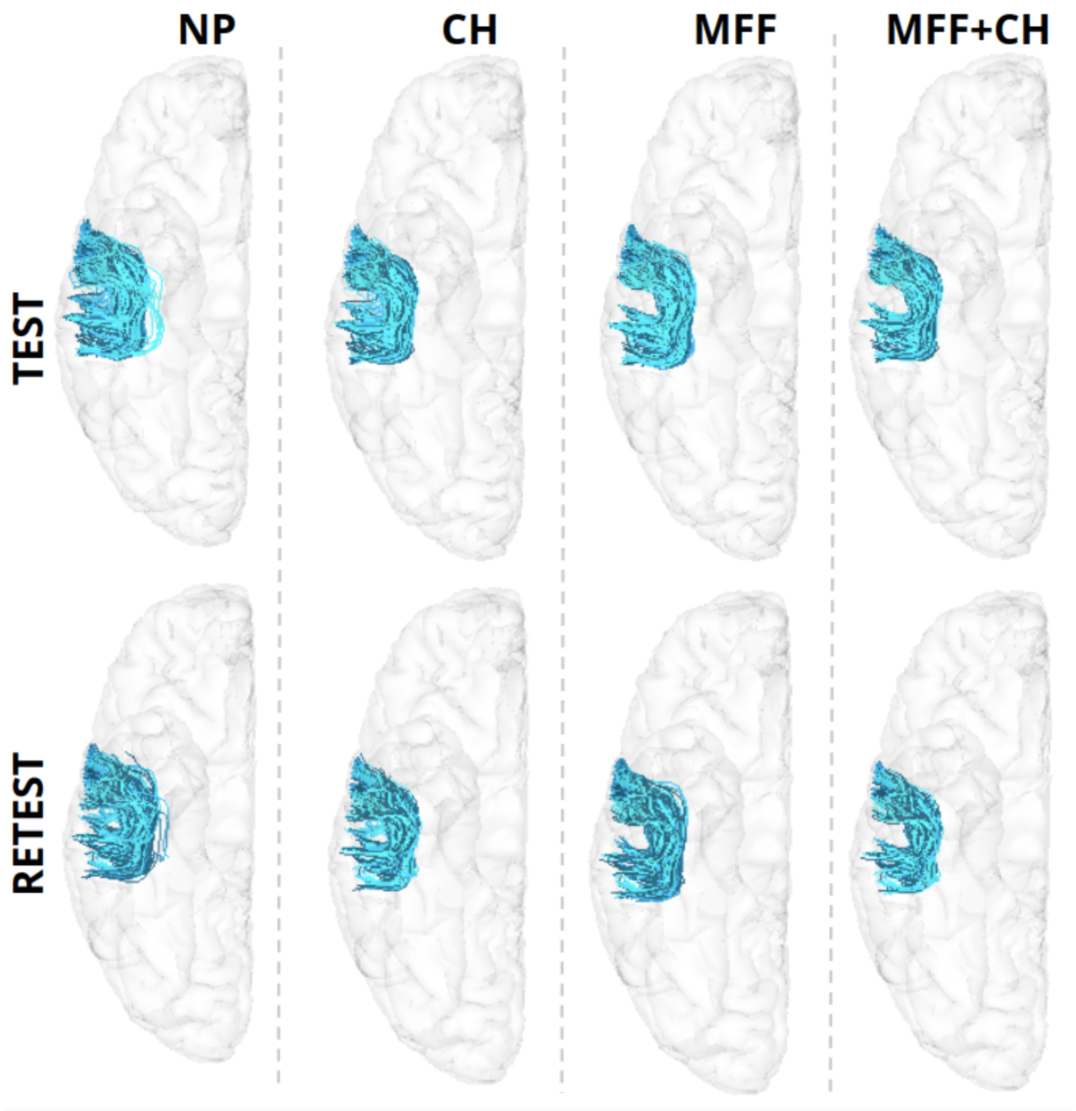

Figure S22. Subject 122317 from the validation set. We show a segmented fiber bundle connecting the Precentral and Supra Marginal gyri from the brain's right hemisphere. NP: No processed, CH: fiber bundle filtered with the filter based on the Convex Hull. MFF: the identification of the main fiber fascicle of the bundle. MFF+CH: the identification of the main fiber fascicle was applied followed by the fiber bundle filter based on the Convex Hull. Axial view.

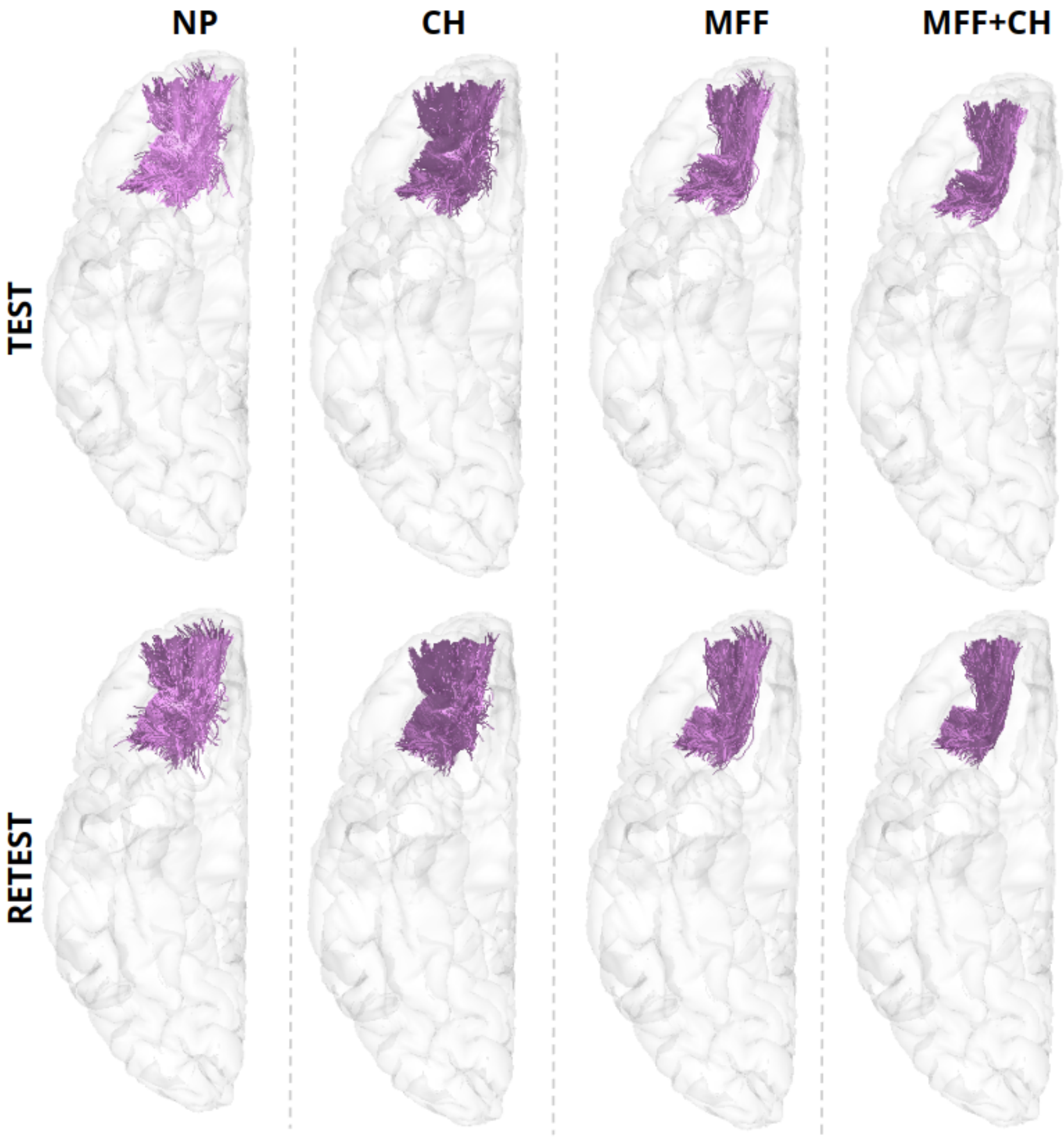

Figure S23. Subject 149337 from the validation set. We show a segmented fiber bundle connecting the Rostral Middle Frontal and Lateral Orbito Frontal gyri from the brain's right hemisphere. NP: No processed, CH: fiber bundle filtered with the filter based on the Convex Hull. MFF: the identification of the main fiber fascicle of the bundle. MFF+CH: the identification of the main fiber fascicle was applied followed by the fiber bundle filter based on the Convex Hull. Axial view.

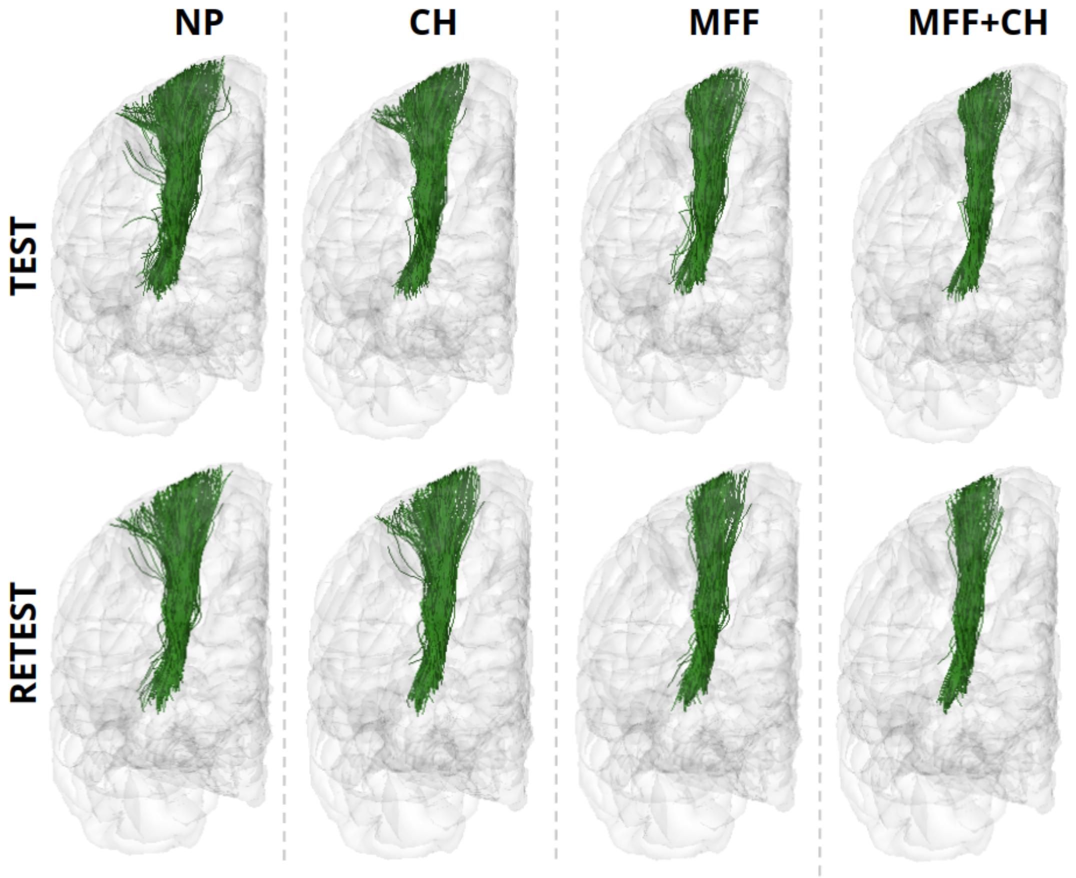

Figure S24. Subject 204521 from the validation set. We show a segmented fiber bundle connecting the Postcentral and Insula gyri from the brain's right hemisphere. NP: No processed, CH: fiber bundle filtered with the filter based on the Convex Hull. MFF: the identification of the main fiber fascicle of the bundle. MFF+CH: the identification of the main fiber fascicle was applied followed by the fiber bundle filter based on the Convex Hull. Coronal view.

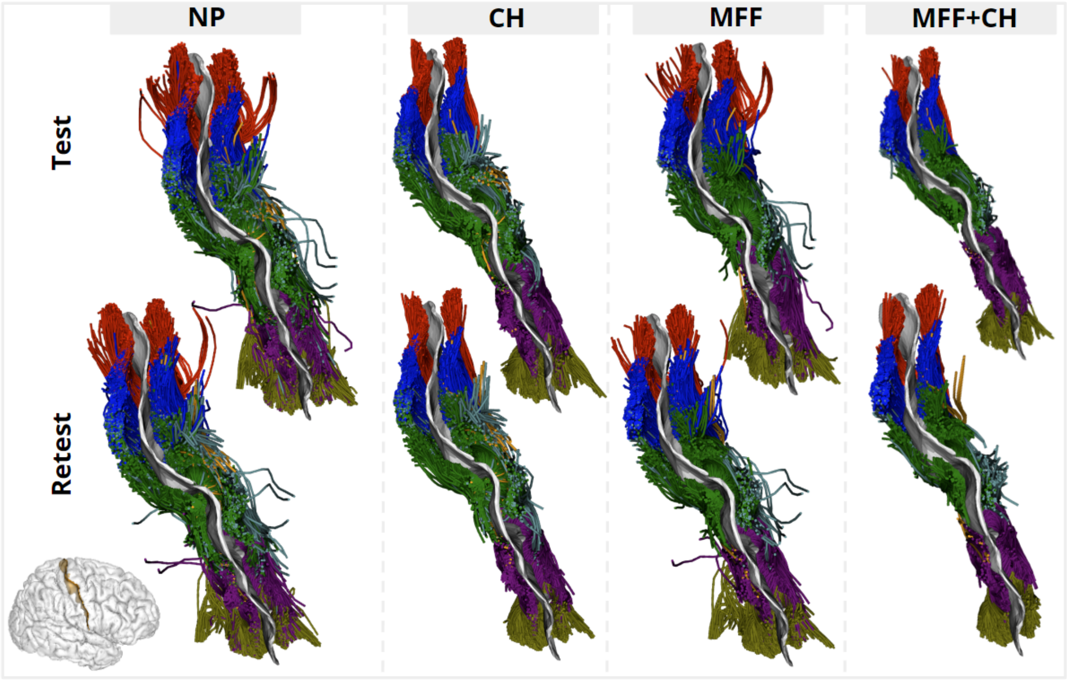

Figure S25. Seven bundles surrounding the central sulcus from the right hemisphere of subject 783462. We observed that the filtered fiber bundles properly follow the sulcus morphology.

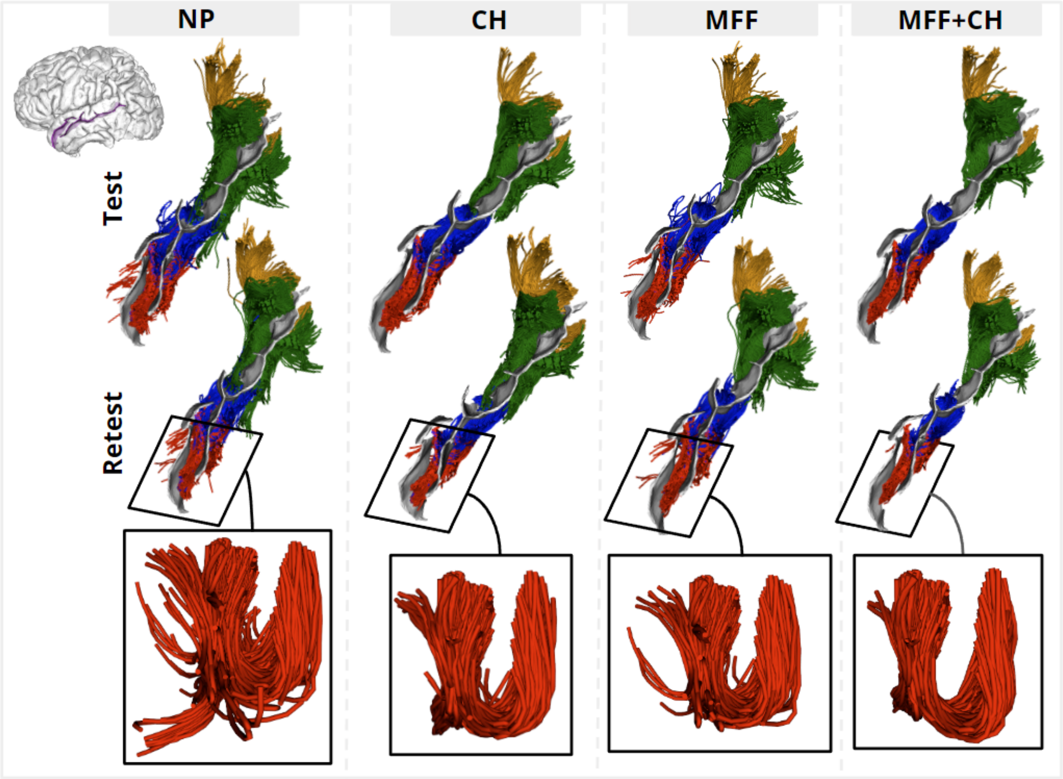

Figure S26. Four bundles surround the superior temporal sulcus from the left hemisphere of subject 194140. The Convex Hull filter allowed us to enhance the quality of the segmented bundles. We show a zoomed view of the red bundle to better illustrate the effect of the fiber bundle filter based on the Convex Hull. Each bundle is shown with a different color.

S13 SEGMENTED BUNDLES FROM THE ABIDE-II DATABASE

Figures S27-S29 show segmented bundles from control subjects of the ABIDE-II database.

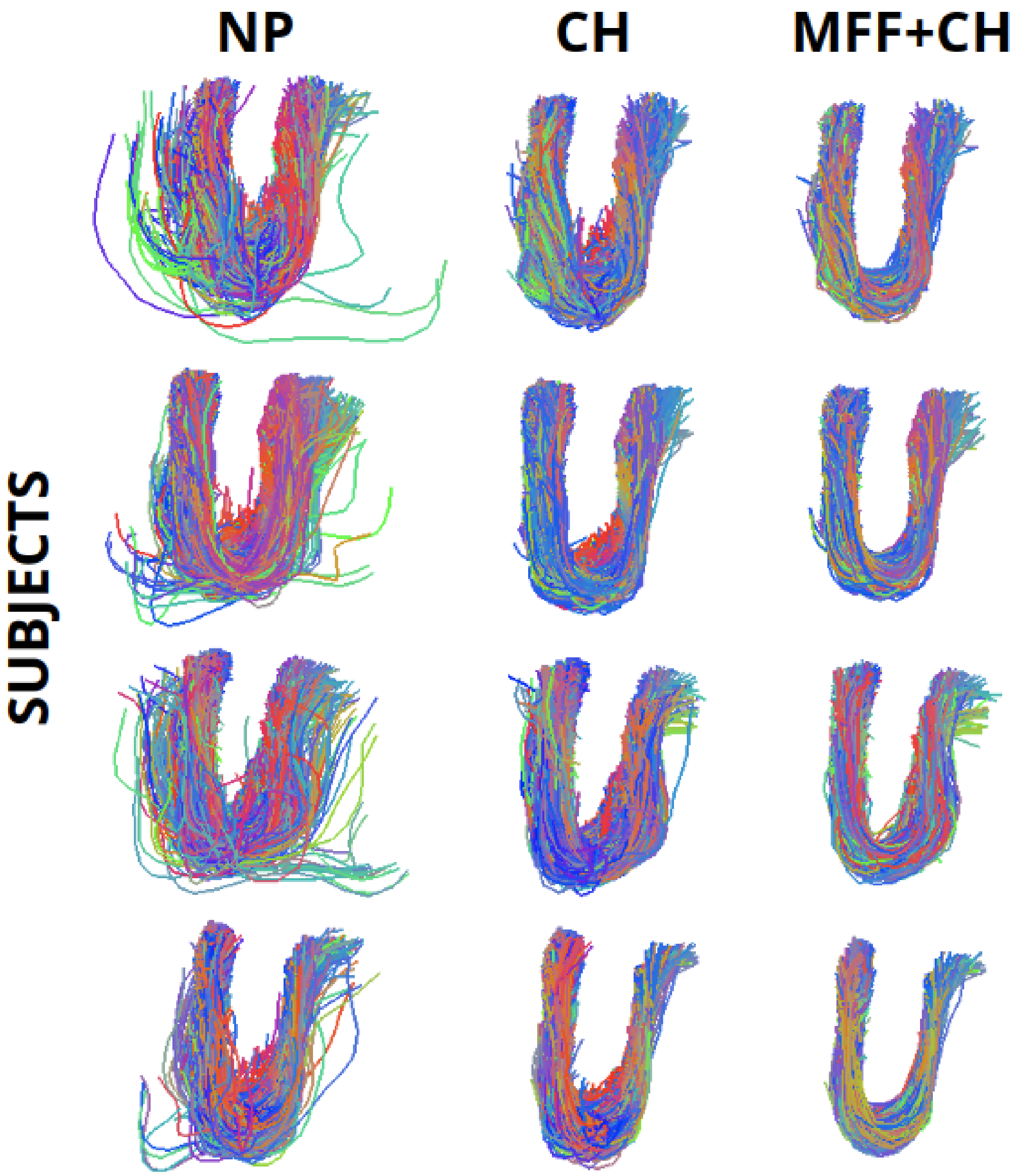

Figure S27. We show a segmented fiber bundle connecting the Precentral and Postcentral gyri from the brain's right hemisphere. NP: No processed, CH: fiber bundle filtered with the filter based on the Convex Hull. MFF+CH: the identification of the main fiber fascicle was applied followed by the fiber bundle filter based on the Convex Hull. Each row corresponds to the bundles of a different control subject.

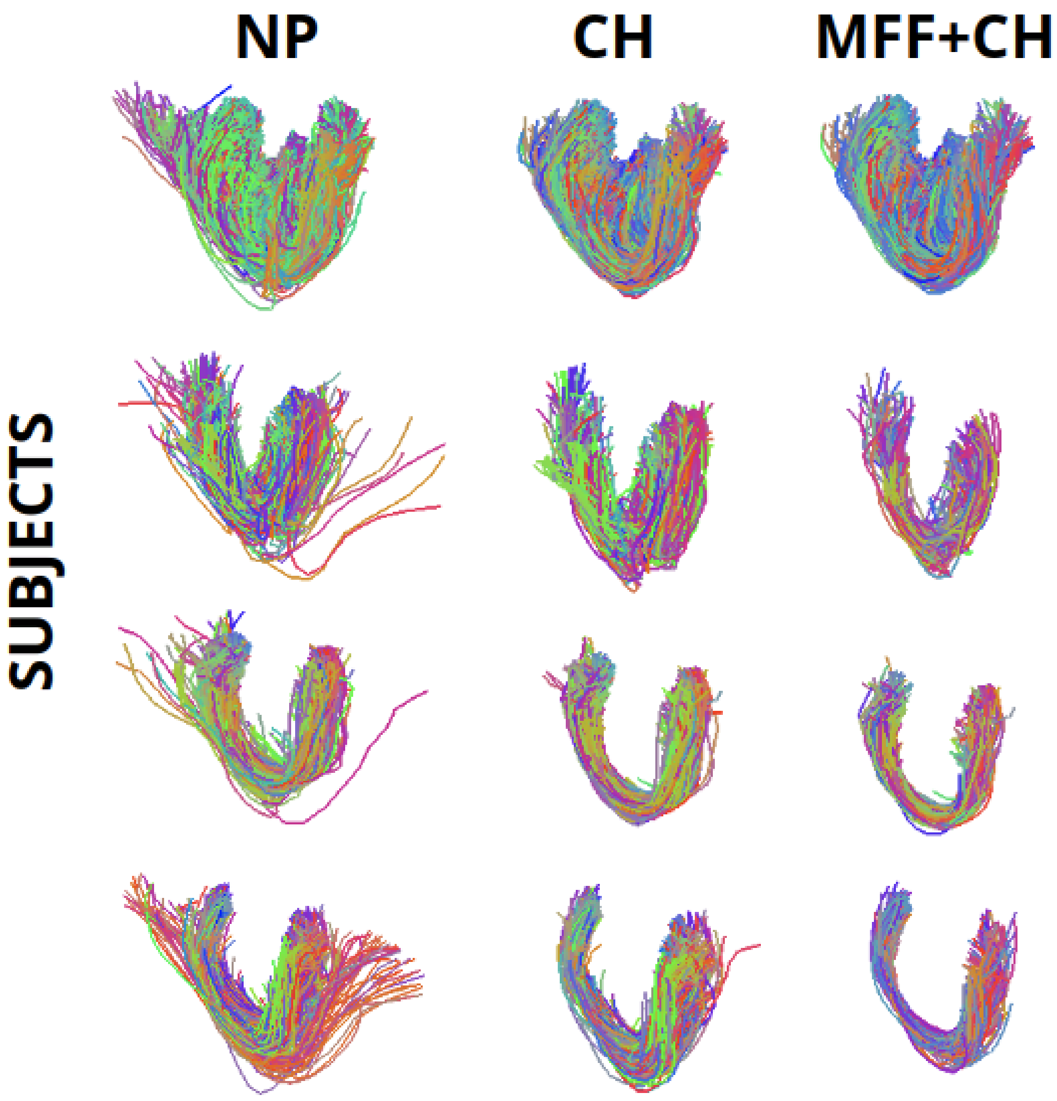

Figure S28. We show a segmented fiber bundle connecting the Caudal Middle Frontal and Superior Frontal gyri from the brain's left hemisphere. NP: No processed, CH: fiber bundle filtered with the filter based on the Convex Hull. MFF+CH: the identification of the main fiber fascicle was applied followed by the fiber bundle filter based on the Convex Hull. Each row corresponds to the bundles of a different control subject.

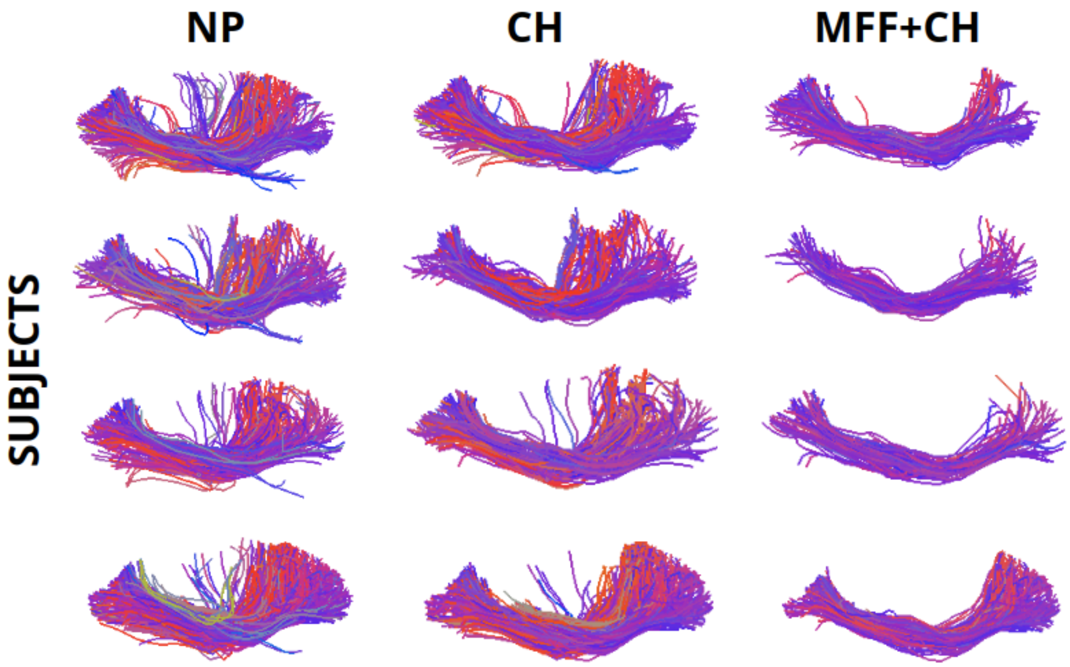

Figure S29. We show a segmented fiber bundle connecting the Precentral and Superior Frontal gyri from the brain's left hemisphere. NP: No processed, CH: fiber bundle filtered with the filter based on the Convex Hull. MFF+CH: the identification of the main fiber fascicle was applied followed by the fiber bundle filter based on the Convex Hull. Each row corresponds to the bundles of a different control subject.

S14 ADDITIONAL RESULTS OF THE ABIDE-II DATABASE

In Tables S7-S15 bundles are written with the abbreviations from Table S6. Also, the labels rh and lh indicate whether the fiber bundle belongs to the left or right brain's hemisphere.

Region (gyrus)	Abb.	Region (gyrus)	Abb.
Bankssts	B	Pars triangularis	Tr
Caudal anterior cingulate	CAC	Pericalcarine	PeCa
Caudal middle frontal	CMF	Postcentral	PoC
Cuneus	Cu	Posterior cingulate	PoCi
Entorhinal	En	Precentral	PrC
Fusiform	Fu	Precuneus	PrCu
Inferior parietal	IP	Rostral anterior cingulate	RAC
Inferior temporal	IT	Rostral middle frontal	RMF
Isthmus cingulate	IC	Superior frontal	SF
Lateral occipital	LO	Superior parietal	SP
Lateral orbitofrontal	LOF	Superior temporal	ST
Lingual	Li	Supramarginal	SM
Medial orbitofrontal	MOF	Transverse temporal	TT
Middle temporal	MT	Insula	In
Parahippocampal	PH	Frontalpole	FPol
Paracentral	PC	Temporalpole	TPol
Pars opercularis	Op		
Pars orbitalis	Or		

Table S6. Abbreviations of each region connected by a short bundle, according to the Desikan-Killiany atlas (Desikan et al., 2006).

Bundle	FA Control	FA ASD	uncorrected p-value	Cohen's d
lh_MT-TPol_0	0.32±0.02	0.31±0.02	0.024	0.71
rh_RMF-SF_1	0.29±0.02	0.28±0.02	0.042	0.44

Table S7. Bundles with statistical difference (uncorrected p-value < 0.05) between control and ASD subjects for the mean FA. For each bundle, the mean FA averaged across subjects is shown (average ± standard deviation). Neither the identification of the main fiber fascicle nor the fiber bundle filter based on the Convex Hull were applied (NP bundles).

Bundle	FA Control	FA ASD	uncorrected p-value	Cohen's d
lh_MT-TPol_0	0.31±0.03	0.30±0.02	0.015	0.81
lh_IT-In_-1	0.38±0.03	0.36±0.02	0.030	0.68
lh_ST-SM_0	0.28±0.02	0.27±0.01	0.005	0.90
lh_PoC-PrC_4	0.26±0.02	0.25±0.02	0.047	0.62

Table S8. Bundles with statistical difference (uncorrected p-value < 0.05) between control and ASD subjects for the mean FA. For each bundle, the mean FA averaged across subjects is shown (average ± standard deviation). The fiber bundle filter based on the Convex Hull was applied (CH bundles).

Bundle	FA Control	FA ASD	uncorrected p-value	Cohen's d
lh_MOF-ST_0	0.35±0.03	0.34±0.02	0.039	0.65
lh_MT-TPol_0	0.34±0.04	0.32±0.03	0.042	0.63
lh_Tr-Tr_0	0.27±0.01	0.26±0.02	0.007	0.61
rh_Op-PrC_2	0.33±0.03	0.31±0.03	0.049	0.61
lh_ST-SM_0	0.29±0.02	0.28±0.02	0.006	0.87
lh_MT-In_1	0.36±0.02	0.34±0.02	0.023	0.71
lh_IT-ST_0	0.33±0.03	0.31±0.02	0.049	0.61
lh_B-B_0	0.31±0.02	0.33±0.03	0.016	-0.76

Table S9. Bundles with statistical difference (uncorrected p-value < 0.05) between control and ASD subjects of the mean FA. For each bundle, the mean FA averaged across subjects is shown (average $\pm$ standard deviation). The identification of the main fiber fascicle and the fiber bundle filter based on the Convex Hull were applied (MFF+CH bundles).

Bundle	MD Control	MD ASD	uncorrected p-value	Cohen's d
lh_MOF-ST_0	0.89±0.02	0.91±0.03	0.018	-0.75
rh_IP-MT_1	0.80±0.03	0.82±0.03	0.038	-0.71
rh_ST-In_0	0.86±0.02	0.88±0.02	0.021	-0.72
lh_LOF-In_2	0.83±0.01	0.85±0.02	0.020	-0.73
lh_IP-MT_1	0.81±0.02	0.82±0.03	0.049	-0.61
lh_IP-IP_1	0.82±0.02	0.83±0.03	0.044	-0.63
lh_B-IP_0	0.81±0.02	0.83±0.03	0.042	-0.63
lh_MT-In_1	0.86±0.02	0.88±0.02	0.049	-0.61
lh_ST-In_0	0.87±0.02	0.88±0.02	0.043	-0.63
lh_LOF-LOF_0	0.84±0.02	0.86±0.03	0.038	-0.65
lh_Or-In_0	0.83±0.02	0.84±0.02	0.040	-0.64
lh_LO-MT_0	0.80±0.02	0.82±0.03	0.031	-0.67
lh_MT-SM_1	0.81±0.02	0.82±0.02	0.048	-0.61
rh_PeCa-SP_0	0.84±0.03	0.86±0.03	0.027	-0.48
lh_IP-SM_1	0.81±0.03	0.83±0.03	0.042	-0.63

Table S10. Bundles with statistical difference (uncorrected p-value < 0.05) between control and ASD subjects for the mean MD. For each bundle, the mean MD averaged across subjects is shown (average $\pm$ standard deviation). Neither the identification of the main fiber fascicle nor the fiber bundle filter based on the Convex Hull were applied (NP bundles). MD values are in $\times 10^{-3} mm^2/s$.

Bundle	MD Control	MD ASD	uncorrected p-value	Cohen's d
rh_Fu-IT_0	0.81±0.02	0.82±0.03	0.047	-0.44
rh_IP-MT_1	0.80±0.03	0.82±0.03	0.032	-0.68
rh_IP-IP_2	0.81±0.03	0.82±0.03	0.046	-0.62
rh_ST-In_0	0.87±0.02	0.88±0.03	0.018	-0.74
lh_LOF-In_2	0.83±0.01	0.85±0.03	0.048	-0.61
lh_IP-MT_1	0.81±0.02	0.82±0.03	0.039	-0.64
lh_IP-LO_0	0.81±0.02	0.82±0.03	0.048	-0.61
lh_IP-IP_1	0.82±0.02	0.84±0.03	0.029	-0.68
lh_IP-ST_0	0.82±0.03	0.83±0.03	0.038	-0.65
lh_ST-SM_0	0.82±0.02	0.84±0.03	0.029	-0.68
rh_IC-SP_0	0.84±0.03	0.86±0.04	0.039	-0.64
lh_IP-SM_1	0.81±0.02	0.83±0.03	0.032	-0.67
rh_IC-IC_0	0.86±0.03	0.88±0.04	0.040	-0.63

Table S11. Bundles with statistical difference (uncorrected p-value < 0.05) between control and ASD subjects for the mean MD. For each bundle, the mean MD averaged across subjects is shown (average $\pm$ standard deviation). The fiber bundle filter based on the Convex Hull was applied (CH bundles). MD values are in $\times 10^{-3} mm^2/s$.

Bundle	MD Control	MD ASD	uncorrected p-value	Cohen's d
lh_MT-TPol_0	0.85±0.02	0.87±0.03	0.030	-0.68
rh_IP-MT_1	0.80±0.03	0.82±0.03	0.032	-0.69
rh_B-MT_0	0.82±0.02	0.83±0.03	0.044	-0.62
rh_IP-IP_2	0.80±0.03	0.82±0.03	0.047	-0.62
lh_LOF-In_2	0.82±0.02	0.84±0.03	0.042	-0.66
lh_TPol-In_0	0.88±0.02	0.90±0.02	0.021	-0.78
lh_IP-IP_1	0.81±0.02	0.83±0.03	0.021	-0.72
lh_B-IP_0	0.81±0.02	0.82±0.03	0.047	-0.62
lh_IP-ST_0	0.81±0.03	0.84±0.03	0.017	-0.76
lh_ST-SM_0	0.82±0.02	0.84±0.03	0.023	-0.71
lh_MT-In_1	0.85±0.02	0.86±0.02	0.020	-0.73
lh_IP-IT_1	0.80±0.02	0.82±0.03	0.049	-0.61
rh_IT-MT_3	0.80±0.03	0.82±0.03	0.049	-0.50
lh_LO-MT_0	0.80±0.02	0.82±0.03	0.024	-0.70
lh_Cu-Li_0	0.85±0.04	0.89±0.06	0.032	-0.73
rh_IC-SP_0	0.84±0.03	0.86±0.04	0.034	-0.66
lh_LO-SP_1	0.79±0.02	0.81±0.03	0.040	-0.65
rh_B-ST_0	0.80±0.03	0.82±0.03	0.034	-0.66
rh_IC-IC_0	0.85±0.03	0.87±0.04	0.045	-0.60
lh_PoC-SM_0	0.83±0.02	0.85±0.03	0.033	-0.66

Table S12. Bundles with statistical difference (uncorrected p-value < 0.05) between control and ASD subjects for the mean MD. For each bundle, the mean MD averaged across subjects is shown (average $\pm$ standard deviation). The identification of the main fiber fascicle and the fiber bundle filter based on the Convex Hull were applied (MFF+CH bundles). MD values are in $\times 10^{-3} mm^2/s$.

Bundle	RD Control	RD ASD	uncorrected p-value	Cohen's d
lh_MOF-ST_0	0.73±0.03	0.75±0.03	0.023	-0.71
lh_MT-TPol_0	0.71±0.02	0.72±0.03	0.041	-0.64
rh_ST-In_0	0.72±0.02	0.74±0.03	0.042	-0.63
lh_MT-In_1	0.70±0.02	0.71±0.02	0.048	-0.62
rh_CAC-PrCu_0	0.67±0.03	0.69±0.04	0.038	-0.57
lh_LO-MT_0	0.67±0.02	0.69±0.03	0.036	-0.65
rh_IC-PrCu_1	0.69±0.03	0.71±0.03	0.045	-0.62

Table S13. Bundles with statistical difference (uncorrected p-value < 0.05) between control and ASD subjects for the mean RD. For each bundle, the mean RD averaged across subjects is shown (average $\pm$ standard deviation). Neither the identification of the main fiber fascicle nor the fiber bundle filter based on the Convex Hull were applied (NP bundles). RD values are in $\times 10^{-3} mm^2/s$

Bundle	RD Control	RD ASD	uncorrected p-value	Cohen's d
lh_MOF-ST_0	0.73±0.03	0.75±0.03	0.046	-0.62
lh_MT-TPol_0	0.71±0.03	0.73±0.02	0.015	-0.76
lh_Tr-Tr_0	0.70±0.02	0.72±0.03	0.049	-0.61
rh_ST-In_0	0.72±0.02	0.74±0.03	0.049	-0.61
rh_PrCu-PrCu_1	0.70±0.03	0.72±0.04	0.042	-0.58
rh_PrCu-PrCu_0	0.72±0.02	0.74±0.04	0.043	-0.63
lh_IT-In_-1	0.68±0.03	0.70±0.03	0.042	-0.63
lh_ST-SM_0	0.70±0.02	0.72±0.03	0.011	-0.80
rh_CAC-PrCu_0	0.66±0.03	0.68±0.04	0.040	-0.61
lh_IP-LO_1	0.68±0.03	0.70±0.03	0.030	-0.68
rh_IC-SP_0	0.64±0.03	0.67±0.04	0.017	-0.75
rh_IC-PrCu_1	0.69±0.03	0.71±0.04	0.028	-0.63
rh_IC-IC_0	0.69±0.04	0.71±0.05	0.035	-0.66

Table S14. Bundles with statistical difference (uncorrected p-value < 0.05) between control and ASD subjects for the mean RD . For each bundle, the mean RD averaged across subjects is shown (average $\pm$ standard deviation). The fiber bundle filter based on the Convex Hull was applied (CH bundles). RD values are in $\times 10^{-3} mm^2/s$.

Bundle	RD Control	RD ASD	uncorrected p-value	Cohen's d
lh_MOF-ST_0	0.72±0.03	0.74±0.04	0.029	-0.69
lh_MT-TPol_0	0.69±0.03	0.72±0.03	0.021	-0.72
lh_Tr-Tr_0	0.71±0.02	0.73±0.03	0.040	-0.64
rh_PrCu-PrCu_1	0.70±0.02	0.72±0.04	0.049	-0.61
rh_PrCu-PrCu_0	0.71±0.03	0.73±0.03	0.048	-0.61
lh_IP-IP_1	0.68±0.03	0.70±0.03	0.042	-0.63
lh_ST-SM_0	0.69±0.02	0.72±0.03	0.007	-0.85
lh_MT-In_1	0.68±0.02	0.70±0.02	0.005	-0.89
rh_CAC-PrCu_0	0.65±0.03	0.67±0.04	0.030	-0.54
lh_IP-LO_1	0.68±0.03	0.70±0.03	0.037	-0.65
lh_LO-MT_0	0.67±0.03	0.69±0.04	0.047	-0.62
rh_IC-SP_0	0.63±0.03	0.66±0.04	0.033	-0.66
lh_ST-In_2	0.65±0.03	0.67±0.03	0.046	-0.62
lh_Or-Or_0	0.69±0.03	0.70±0.03	0.046	-0.62
lh_Fu-IT_0	0.67±0.03	0.69±0.03	0.046	-0.62
rh_IC-IC_0	0.68±0.04	0.70±0.05	0.036	-0.57
lh_PoC-SM_0	0.72±0.02	0.73±0.04	0.039	-0.64

Table S15. Bundles with statistical difference (uncorrected p-value < 0.05) between control and ASD subjects for the mean RD. For each bundle, the mean RD averaged across subjects is shown (average $\pm$ standard deviation). The identification of the main fiber fascicle and the filter based on the Convex Hull were applied (MFF+CH bundles). RD values are in $\times 10^{-3} mm^2/s$.

		MFF	MFF+CP	MFF+SSPD	MFF+FC	MFF+CH
DSC	SRB_1	0.78±0.04	0.78±0.04	0.79±0.05	0.78±0.06	**0.81±0.06**
	SRB_2	0.66±0.05	0.67±0.05	0.68±0.06	**0.68±0.05**	**0.68±0.05**
	SRB_3	**0.56±0.20**	0.55±0.20	0.55±0.20	0.55±0.21	0.56±0.21
	SRB_4	0.76±0.07	0.76±0.06	**0.77±0.07**	0.76±0.06	**0.77±0.07**
AFD	SRB_1	2.24±0.05	2.26±0.06	2.26±0.08	2.26±0.08	**2.29±0.07**
	SRB_2	2.05±0.14	2.08±0.13	2.10±0.14	2.11±0.14	**2.13±0.13**
	SRB_3	1.83±0.38	1.82±0.38	1.83±0.38	1.84±0.37	**1.85±0.39**
	SRB_4	2.24±0.10	2.24±0.09	2.23±0.09	2.25±0.08	**2.28±0.10**
AMD	SRB_1	3.65±0.67	3.57±0.69	3.60±0.70	**3.53±0.75**	3.55±0.72
	SRB_2	4.38±0.29	4.26±0.29	4.29±0.33	**4.23±0.30**	4.24±0.34
	SRB_3	5.32±1.33	5.26±1.31	5.27±1.33	5.07±1.30	**5.07±1.23**
	SRB_4	3.13±0.30	2.96±0.23	3.03±0.24	**2.96±0.22**	3.02±0.25
AD	SRB_1	11.40±0.60	10.72±0.73	10.99±0.72	**10.69±0.77**	10.88±0.69
	SRB_2	12.14±0.68	11.51±0.80	11.44±0.90	11.34±0.88	**11.16±0.85**
	SRB_3	14.02±0.59	12.93±0.74	13.17±0.70	12.90±0.85	**12.55±1.17**
	SRB_4	10.58±0.49	**9.00±0.50**	10.12±0.57	9.63±0.62	9.94±0.57

Table S16. Reproducibility indices from 5 control subjects from the ABIDE-II database (mean$\pm$standard deviation). The fiber bundle filter based on the Convex Hull had the best improvement compared to MFF scores in most indices. The bold values indicate the best score.

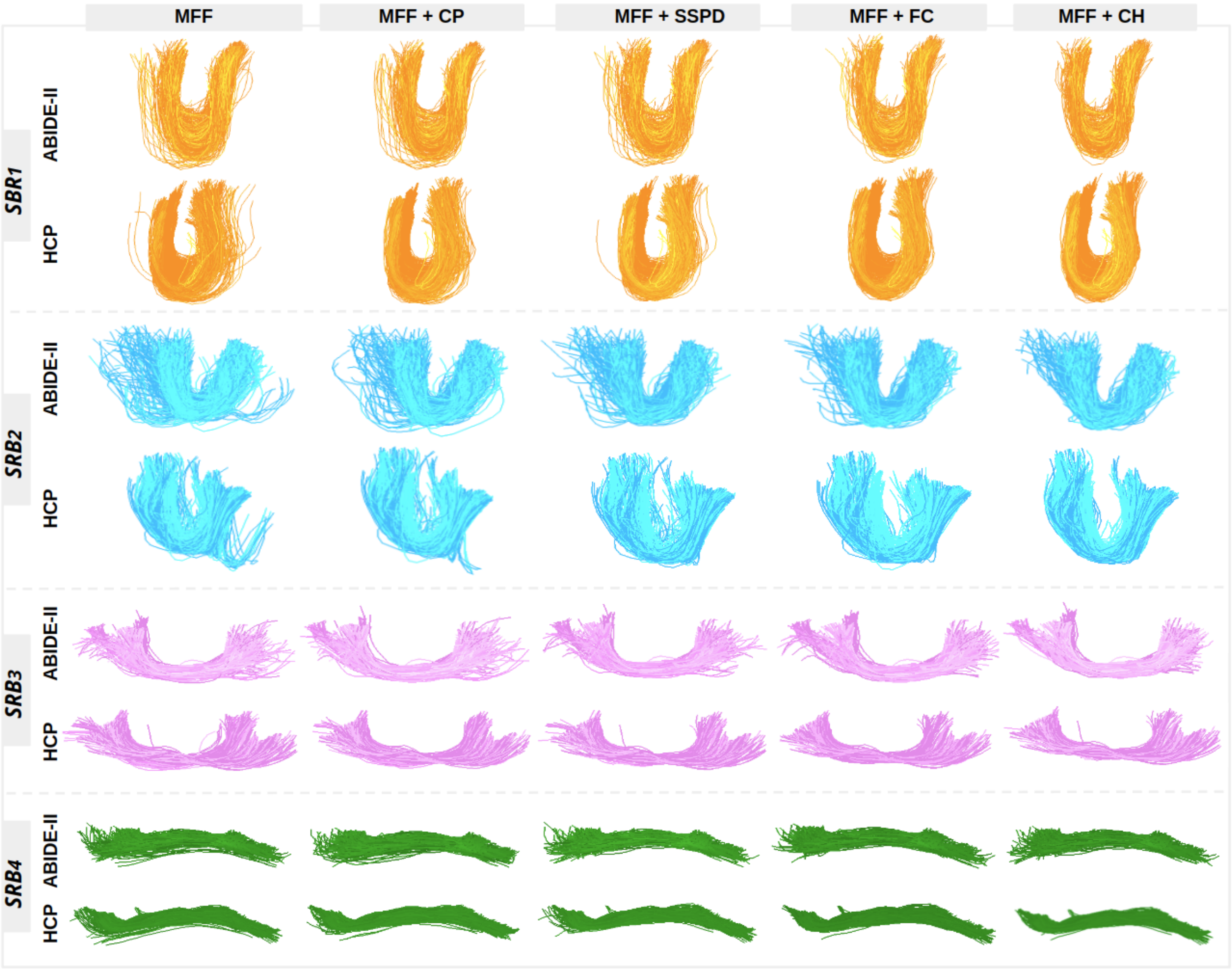

Figure S30. Processed bundles from a subject of the HCP database (ID 125525) and one subject from the ABIDE-II database (ID 29227), using the different filters after applying the MFF identification. In general, the fiber bundle filter based on the MFF + Convex Hull generated smoother bundles with less spurious fibers.

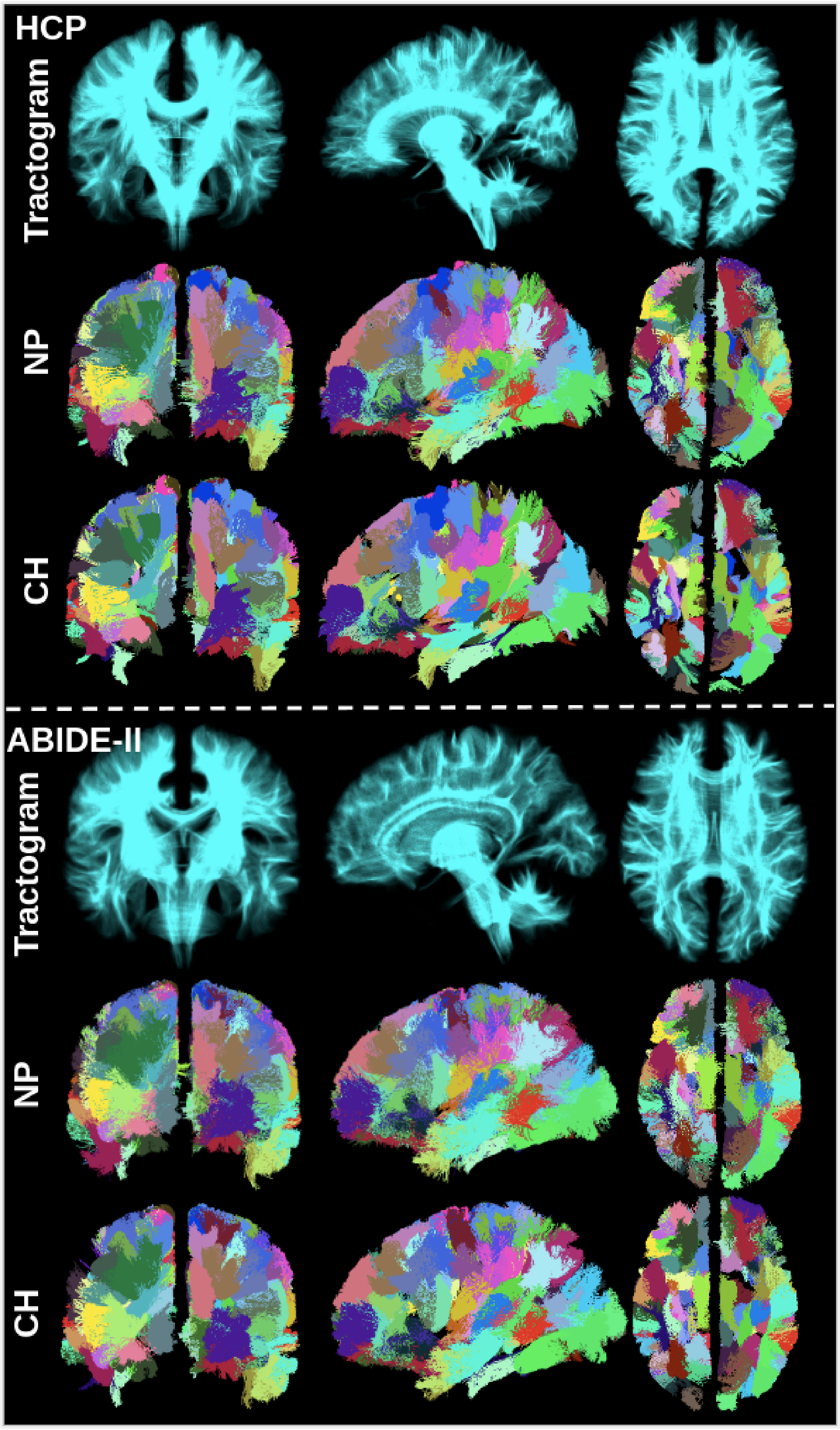

Figure S31. Whole-brain tractogram and 100 randomly selected bundles from a subject of the HCP database and one subject from the ABIDE-II database. NP: bundles without filtering. CH: bundles processed with the fiber bundle filter based on the Convex Hull. The same bundles are shown for both subjects. Tractograms were down-sampled to 500k fibers for visualization purposes.

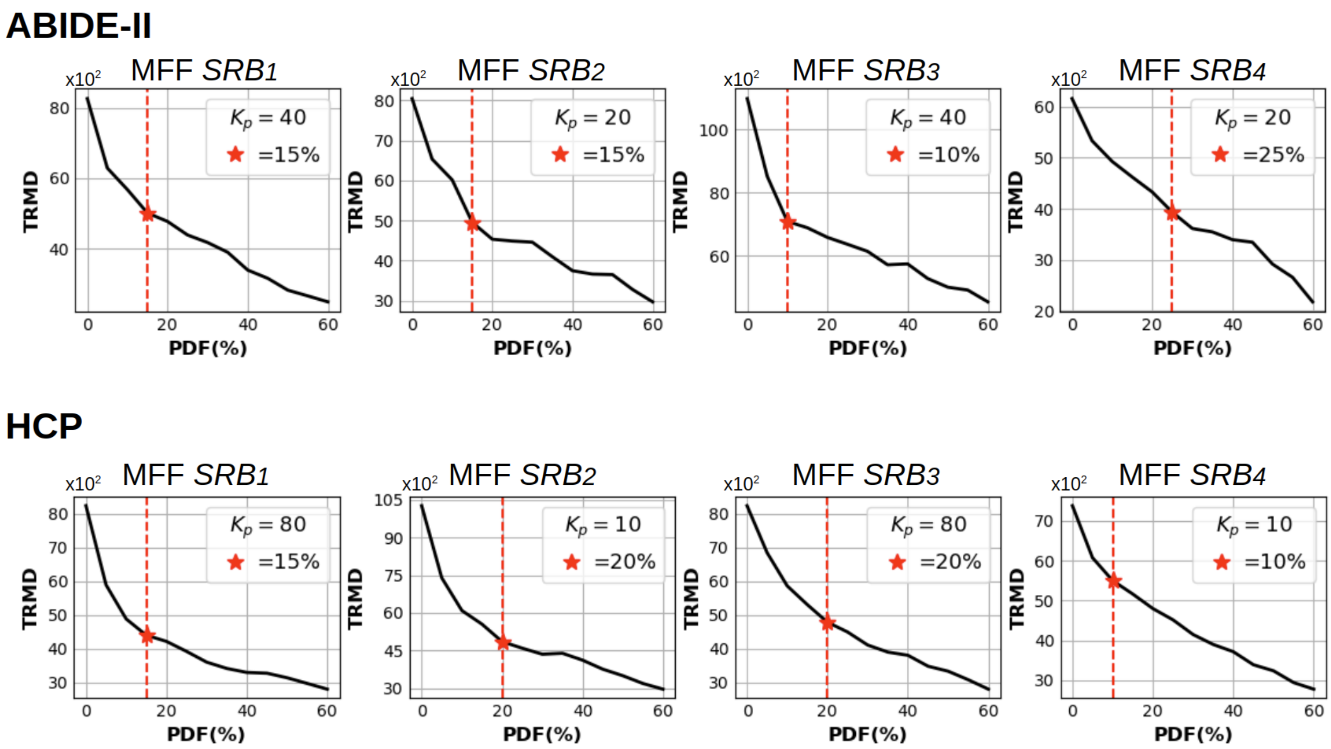

Figure S32. Comparison of the tuning parameters between the HCP database and a subsample of the ABIDE-II database.

	FA		MD		RD	
	HCP	ABIDE-II	HCP	ABIDE-II	HCP	ABIDE-II
SRB_1	0.35±0.03	0.35±0.03	0.85±0.03	0.85±0.03	0.69±0.04	0.69±0.03
SRB_2	0.29±0.01	0.29±0.01	0.81±0.02	0.81±0.02	0.68±0.02	0.68±0.02
SRB_3	0.32±0.02	0.32±0.02	0.80±0.03	0.80±0.02	0.65±0.02	0.66±0.02
SRB_4	0.32±0.04	0.31±0.04	0.85±0.02	0.85±0.02	0.70±0.04	0.70±0.04

Table S17. Mean FA, MD and RD from 5 control subjects of the ABIDE-II database. Bundles were processed with the filter based on the Convex Hull using the HCP and ABIDE-II optimal parameters. The results for the segmented representative bundles are shown. MD and RD values are in $\times 10^{-3} mm^2/s$.

	FA		MD		RD	
	HCP	ABIDE-II	HCP	ABIDE-II	HCP	ABIDE-II
SRB_1	0.36±0.01	0.36±0.02	0.86±0.03	0.86±0.03	0.69±0.03	0.69±0.03
SRB_2	0.29±0.01	0.29±0.01	0.81±0.02	0.81±0.02	0.68±0.02	0.68±0.01
SRB_3	0.34±0.02	0.34±0.02	0.80±0.02	0.80±0.03	0.65±0.02	0.65±0.02
SRB_4	0.32±0.04	0.31±0.05	0.85±0.02	0.85±0.02	0.69±0.04	0.69±0.04

Table S18. Mean FA, MD and RD from 5 control subjects of the ABIDE-II database. Bundles were processed with the identification of the main fiber fascicle and filter based on the Convex Hull using the HCP and ABIDE-II optimal parameters. The results for the segmented representative bundles are shown. MD and RD values are in $\times 10^{-3} mm^2/s$.

	FA Control			FA ASD			FA Control - FA ASD		
Bundle	NP	CH	MFF+CH	NP	CH	MFF+CH	NP	CH	MFF+CH
lh_MOF-ST_0	0.32±0.02	0.33±0.02	0.35±0.03	0.31±0.02	0.32±0.02	0.34±0.02	0.01	0.01	0.01
lh_MT-TPol_0	0.32±0.02	0.31±0.03	0.34±0.04	0.31±0.02	0.30±0.02	0.32±0.03	0.01	0.01	0.02
rh_RMF-SF_1	0.29±0.02	0.28±0.02	0.29±0.02	0.28±0.02	0.27±0.02	0.27±0.02	0.01	0.01	0.02
rh_Op-PrC_2	0.32±0.03	0.32±0.03	0.33±0.03	0.31±0.03	0.30±0.03	0.31±0.03	0.01	0.02	0.02
lh_B-B_0	0.31±0.02	0.31±0.02	0.31±0.02	0.32±0.02	0.32±0.02	0.33±0.03	-0.01	-0.01	-0.02
lh_Tr-Tr_0	0.29±0.01	0.28±0.01	0.27±0.01	0.28±0.02	0.27±0.02	0.26±0.02	0.01	0.01	0.01
lh_PoC-PrC_4	0.27±0.02	0.26±0.02	0.27±0.02	0.27±0.02	0.25±0.02	0.26±0.02	0.00	0.01	0.01
lh_ST-SM_0	0.29±0.01	0.28±0.02	0.29±0.02	0.29±0.02	0.27±0.01	0.28±0.02	0.00	0.01	0.01
lh_IT-ST_0	0.34±0.02	0.34±0.03	0.33±0.03	0.33±0.02	0.32±0.02	0.31±0.02	0.01	0.02	0.02
lh_IT-In_-1	0.37±0.03	0.38±0.03	0.39±0.03	0.36±0.02	0.36±0.02	0.38±0.02	0.01	0.02	0.01
lh_MT-In_1	0.34±0.02	0.34±0.02	0.36±0.02	0.33±0.02	0.33±0.02	0.34±0.02	0.01	0.01	0.02
						Average	0.006	0.011	0.012

Table S19. Mean and standard deviation of FA values for bundles before and after filtering with CH and MFF+CH. Bundles listed are those with significant differences between control and ASD subjects from Tables S7, S8, and S9.

	MD Control			MD ASD			MD Control - MD ASD		
Bundle	NP	CH	MFF+CH	NP	CH	MFF+CH	NP	CH	MFF+CH
lh_LO-SP_1	0.81±0.02	0.81±0.02	0.79±0.02	0.82±0.03	0.82±0.03	0.81±0.03	-0.01	-0.01	-0.02
lh_IP-IP_1	0.82±0.02	0.82±0.02	0.81±0.02	0.83±0.03	0.84±0.03	0.83±0.03	-0.01	-0.02	-0.02
rh_IC-SP_0	0.84±0.03	0.84±0.03	0.84±0.03	0.86±0.04	0.86±0.04	0.86±0.04	-0.02	-0.02	-0.02
lh_B-IP_0	0.81±0.02	0.81±0.02	0.81±0.02	0.83±0.03	0.82±0.03	0.82±0.03	-0.02	-0.01	-0.01
lh_Or-In_0	0.83±0.02	0.82±0.01	0.82±0.02	0.84±0.02	0.83±0.02	0.83±0.03	-0.01	-0.01	-0.01
rh_Fu-IT_0	0.81±0.03	0.81±0.02	0.81±0.02	0.83±0.03	0.82±0.03	0.83±0.03	-0.02	-0.01	-0.02
rh_B-ST_0	0.82±0.02	0.82±0.03	0.80±0.03	0.83±0.03	0.83±0.03	0.82±0.03	-0.01	-0.01	-0.02
lh_IP-LO_0	0.81±0.02	0.81±0.02	0.80±0.02	0.82±0.03	0.82±0.03	0.82±0.03	-0.01	-0.01	-0.02
lh_IP-SM_1	0.81±0.03	0.81±0.02	0.81±0.02	0.83±0.03	0.83±0.03	0.82±0.03	-0.02	-0.02	-0.01
lh_MT-SM_1	0.81±0.02	0.81±0.02	0.80±0.03	0.82±0.02	0.82±0.03	0.82±0.03	-0.01	-0.01	-0.02
rh_ST-In_0	0.86±0.02	0.87±0.02	0.86±0.02	0.88±0.02	0.88±0.03	0.88±0.03	-0.02	-0.01	-0.02
rh_PeCa-SP_0	0.84±0.03	0.85±0.04	0.84±0.05	0.86±0.03	0.86±0.03	0.86±0.04	-0.02	-0.01	-0.02
rh_IC-IC_0	0.87±0.03	0.86±0.03	0.85±0.03	0.88±0.04	0.88±0.04	0.87±0.04	-0.01	-0.02	-0.02
rh_B-MT_0	0.82±0.02	0.82±0.02	0.82±0.02	0.83±0.03	0.83±0.03	0.83±0.03	-0.01	-0.01	-0.01
lh_IP-MT_1	0.81±0.02	0.81±0.02	0.81±0.02	0.82±0.03	0.82±0.03	0.82±0.03	-0.01	-0.01	-0.01
lh_ST-SM_0	0.82±0.02	0.82±0.02	0.82±0.02	0.84±0.03	0.84±0.03	0.84±0.03	-0.02	-0.02	-0.02
lh_IP-ST_0	0.81±0.02	0.82±0.03	0.81±0.03	0.83±0.03	0.83±0.03	0.84±0.03	-0.02	-0.01	-0.03
lh_MT-TPol_0	0.86±0.02	0.86±0.02	0.85±0.02	0.87±0.02	0.87±0.02	0.87±0.03	-0.01	-0.01	-0.02
rh_IT-MT_3	0.81±0.02	0.81±0.02	0.80±0.03	0.82±0.03	0.82±0.03	0.82±0.03	-0.01	-0.01	-0.02
lh_LOF-In_2	0.83±0.01	0.83±0.01	0.82±0.02	0.85±0.02	0.85±0.03	0.84±0.03	-0.02	-0.02	-0.02
lh_IP-IT_1	0.80±0.02	0.80±0.02	0.80±0.02	0.82±0.03	0.82±0.03	0.82±0.03	-0.02	-0.02	-0.02
lh_Cu-Li_0	0.89±0.04	0.89±0.04	0.85±0.04	0.90±0.05	0.91±0.05	0.89±0.06	-0.01	-0.02	-0.04
lh_MOF-ST_0	0.89±0.02	0.90±0.03	0.90±0.03	0.91±0.03	0.91±0.03	0.92±0.04	-0.02	-0.01	-0.02
lh_LOF-LOF_0	0.84±0.02	0.84±0.02	0.85±0.02	0.86±0.03	0.85±0.02	0.86±0.03	-0.02	-0.01	-0.01
lh_LO-MT_0	0.80±0.02	0.80±0.02	0.80±0.02	0.82±0.03	0.82±0.04	0.82±0.03	-0.02	-0.02	-0.02
lh_TPol-In_0	0.89±0.02	0.89±0.02	0.88±0.02	0.90±0.03	0.90±0.02	0.90±0.02	-0.01	-0.01	-0.02
lh_ST-In_0	0.87±0.02	0.87±0.02	0.87±0.02	0.88±0.02	0.88±0.03	0.88±0.02	-0.01	-0.01	-0.01
rh_IP-MT_1	0.80±0.03	0.80±0.03	0.80±0.03	0.82±0.03	0.82±0.03	0.82±0.03	-0.02	-0.02	-0.02
rh_IP-IP_2	0.80±0.03	0.81±0.03	0.80±0.03	0.82±0.03	0.82±0.03	0.82±0.03	-0.02	-0.01	-0.02
lh_PoC-SM_0	0.83±0.02	0.83±0.02	0.83±0.02	0.84±0.03	0.85±0.03	0.85±0.03	-0.01	-0.02	-0.02
lh_MT-In_1	0.86±0.02	0.86±0.02	0.85±0.02	0.88±0.02	0.88±0.02	0.86±0.02	-0.02	-0.02	-0.01
						Average	-0.015	-0.014	-0.018

Table S20. Mean and standard deviation of MD values for bundles before and after filtering with CH and MFF+CH. Bundles listed are those with significant differences between control and ASD subjects from Tables S10, S11, and S12.

	RD Control			RD ASD			RD Control - RD ASD		
Bundle	NP	CH	MFF+CH	NP	CH	MFF+CH	NP	CH	MFF+CH
lh_IP-LO_1	0.68±0.02	0.68±0.03	0.68±0.03	0.69±0.03	0.70±0.03	0.70±0.03	-0.01	-0.02	-0.02
lh_IP-IP_1	0.68±0.02	0.69±0.02	0.68±0.03	0.70±0.03	0.70±0.03	0.70±0.03	-0.02	-0.01	-0.02
rh_IC-SP_0	0.65±0.03	0.64±0.03	0.63±0.03	0.67±0.04	0.67±0.04	0.66±0.04	-0.02	-0.03	-0.03
rh_PrCu-PrCu_1	0.70±0.03	0.70±0.03	0.70±0.02	0.71±0.03	0.72±0.04	0.72±0.04	-0.01	-0.02	-0.02
lh_Or-Or_0	0.69±0.02	0.70±0.03	0.69±0.03	0.71±0.03	0.71±0.03	0.70±0.03	-0.02	-0.01	-0.01
rh_ST-In_0	0.72±0.02	0.72±0.02	0.72±0.02	0.74±0.03	0.74±0.03	0.74±0.04	-0.02	-0.02	-0.02
rh_IC-IC_0	0.68±0.03	0.69±0.04	0.68±0.04	0.71±0.05	0.71±0.05	0.70±0.05	-0.03	-0.02	-0.02
rh_IC-PrCu_1	0.69±0.03	0.69±0.03	0.69±0.03	0.71±0.03	0.71±0.04	0.71±0.04	-0.02	-0.02	-0.02
lh_ST-SM_0	0.69±0.02	0.70±0.02	0.69±0.02	0.71±0.03	0.72±0.03	0.72±0.03	-0.02	-0.02	-0.03
lh_MT-TPol_0	0.71±0.02	0.71±0.03	0.69±0.03	0.72±0.03	0.73±0.02	0.72±0.03	-0.01	-0.02	-0.03
rh_PrCu-PrCu_0	0.71±0.02	0.72±0.02	0.71±0.03	0.73±0.05	0.74±0.04	0.73±0.03	-0.02	-0.02	-0.02
lh_Fu-IT_0	0.67±0.02	0.67±0.02	0.67±0.03	0.68±0.03	0.69±0.03	0.69±0.03	-0.01	-0.02	-0.02
lh_Tr-Tr_0	0.70±0.02	0.70±0.02	0.71±0.02	0.71±0.03	0.72±0.03	0.73±0.03	-0.01	-0.02	-0.02
lh_ST-In_2	0.66±0.02	0.66±0.03	0.65±0.03	0.67±0.04	0.67±0.04	0.67±0.03	-0.01	-0.01	-0.02
lh_MOF-ST_0	0.73±0.03	0.73±0.03	0.72±0.03	0.75±0.03	0.75±0.03	0.74±0.04	-0.02	-0.02	-0.02
lh_LO-MT_0	0.67±0.02	0.68±0.03	0.67±0.03	0.69±0.03	0.69±0.04	0.69±0.04	-0.02	-0.01	-0.02
rh_CAC-PrCu_0	0.67±0.03	0.66±0.03	0.65±0.03	0.69±0.04	0.68±0.04	0.67±0.04	-0.02	-0.02	-0.02
lh_IT-In_-1	0.69±0.03	0.68±0.03	0.66±0.03	0.70±0.03	0.70±0.03	0.68±0.03	-0.01	-0.02	-0.02
lh_PoC-SM_0	0.71±0.02	0.72±0.02	0.72±0.02	0.72±0.03	0.73±0.04	0.73±0.04	-0.01	-0.01	-0.01
lh_MT-In_1	0.70±0.02	0.70±0.02	0.68±0.02	0.71±0.02	0.71±0.03	0.70±0.02	-0.01	-0.01	-0.02
						Average	-0.016	-0.017	-0.020

Table S21. Mean and standard deviation of RD values for bundles before and after filtering with CH and MFF+CH. Bundles listed are those with significant differences between control and ASD subjects from Tables S13, S14, and S15.

REFERENCES

Aggarwal, C. C. and Reddy, C. K. (2018). *Data Clustering Algorithms and Applications* (Taylor Francis Group)

Colby, J. B., Soderberg, L., Lebel, C., Dinov, I. D., Thompson, P. M., and Sowell, E. R. (2012). Along-tract statistics allow for enhanced tractography analysis. *NeuroImage* 59, 3227–3242. doi:10.1016/j.neuroimage.2011.11.004

Desikan, R. S., Ségonne, F., Fischl, B., Quinn, B. T., Dickerson, B. C., Blacker, D., et al. (2006). An automated labeling system for subdividing the human cerebral cortex on MRI scans into gyral based regions of interest. *NeuroImage* 31, 968–980. doi:10.1016/j.neuroimage.2006.01.021

Garyfallidis, E., Côté, M.-A., Rheault, F., , and Descoteaux, M. (2012). QuickBundles, a method for tractography simplification. *Frontiers in Neuroscience* 6. doi:10.3389/fnins.2012.00175

Garyfallidis, E., Ocegueda, O., Wassermann, D., and Descoteaux, M. (2015). Robust and efficient linear registration of white-matter fascicles in the space of streamlines. *NeuroImage* 117, 124–140. doi:10.1016/j.neuroimage.2015.05.016

Guevara, M., Román, C., Houenou, J., Duclap, D., Poupon, C., Mangin, J. F., et al. (2017). Reproducibility of superficial white matter tracts using diffusion-weighted imaging tractography. *NeuroImage* 147, 703–725. doi:10.1016/j.neuroimage.2016.11.066

Horbruegger, M., Loewe, K., Kaufmann, J., Wagner, M., Schippling, S., Pawlitzki, M., et al. (2019). Anatomically constrained tractography facilitates biologically plausible fiber reconstruction of the optic radiation in multiple sclerosis. *NeuroImage: Clinical* 22, 101740. doi:10.1016/j.nicl.2019.101740

Morales, J. L. and Nocedal, J. (2011). Remark on “algorithm 778: L-BFGS-B: Fortran subroutines for large-scale bound constrained optimization”. *ACM Transactions on Mathematical Software* 38, 1–4. doi:10.1145/2049662.2049669

Román, C., Hernández, C., Figueroa, M., Houenou, J., Poupon, C., Mangin, J.-F., et al. (2022). Superficial white matter bundle atlas based on hierarchical fiber clustering over probabilistic tractography data. *NeuroImage* 262, 119550. doi:10.1016/j.neuroimage.2022.119550

Satopaa, V., Albrecht, J., Irwin, D., and Raghavan, B. (2011). Finding a ”kneedle” in a haystack: Detecting knee points in system behavior. In *2011 31st International Conference on Distributed Computing Systems Workshops* (IEEE). doi:10.1109/icdcsw.2011.20

Yeatman, J. D., Dougherty, R. F., Myall, N. J., Wandell, B. A., and Feldman, H. M. (2012). Tract profiles of white matter properties: Automating fiber-tract quantification. *PLoS ONE* 7, e49790. doi:10.1371/journal.pone.0049790
